# Supplementary material for: Diffusionless transformation of soft cubic superstructure from amorphous to simple cubic and body-centered cubic phases
Source: Nat Commun. 2021 Jun 9;12:3477. doi: 10.1038/s41467-021-23631-w (PMC8190294; doi:10.1038/s41467-021-23631-w)
Supplement: Supplementary file 1 — supplementary information [file 41467_2021_23631_MOESM1_ESM.pdf]

Supplementary Information

Diffusionless Transformation of Soft Cubic Superstructure from Amorphous to Simple Cubic and Body-Centered Cubic Phases

Jie Liu[1,2],Wenzhe Liu[3], Bo Guan[4], Bo Wang[3]， Lei Shi[3], Feng Jin[1], Zhigang Zheng[5], Jingxia Wang*[1,2], Tomiki Ikeda[1] and Lei Jiang[1,2]*

[1] Key Laboratory of Bio-inspired Materials and Interfaces Sciences, Technical Institute of Physics and Chemistry, Chinese Academy of Sciences, Beijing, 100190, China

[2] Center of Material Science and Optoelectronics Engineering, School of Future Technology, University of Chinese Academy of Sciences, Beijing 101407, China

[3] Department of Physics, Key Laboratory of Micro-and Nano-Photonic Structures, and State Key Laboratory of Surface Physics, Fudan University, Shanghai 200433, China

[4] Institute of Chemistry, Chinese Academy of Sciences, Beijing, 100190, China

[5] Department of Physics, East China University of Science and Technology, Shanghai 200237, China

Corresponding Author

*E-mail: jingxiawang@mail.ipc.ac.cn (Jingxia Wang).

*E-mail: zgzheng@ecust.edu.cn (Zhigang Zheng).

Supplementary Note 1 Chemicals and corresponding structures

Supplementary Table 1 Abbreviation used in this work.

DLPT	Diffusionless phase transformation
LC	Liquid crystal
BPLCs	Blue phase liquid crystals
PS-BPLCs	Polymer-stabilized blue phase liquid crystals
N*	Cholesteric phase
POM	Polarized optical microscopy
TEM	Transmission electron microscopy
FWHM	Full width at half maxima
DTCs	Double-twisted cylinders
SAXS	Small angle X-ray scattering
Syn-SAXS	Synchrotron small angle X-ray diffraction
ARM	micro-region angle resolved spectra system
FFT	fast Fourier transformation
BPI	Blue phase I
BPI$_{\{100\}}$	BPI with {100} crystal plane parallel to the substrates
BPI$_{\{110\}}$	BPI with {110} crystal plane parallel to the substrates
BPI$_{\{211\}}$	BPI with {211} crystal plane parallel to the substrates
BPII	Blue phase II
BPII$_{\{100\}}$	BPII with {100} crystal plane parallel to the substrates
BPII$_{\{110\}}$	BPII with {110} crystal plane parallel to the substrates
BPIII	Blue phase III
BZ	Brillouin zone
BCC	Body-center cubic
SC	Simple cubic
C6	Coumarin 6
C6-BPLCs	Coumarin 6 doped blue phase liquid crystals
UV	Ultraviolet light
PET	Polyester
PI	Polyimide
1D	One-dimensional
2D	Two-dimensional
3D	Three-dimensional

Supplementary Table 2 Chemical structures of host LCs, reactive monomers, laser dye, and photoinitiator

Component Name	Chemical Structure	Synonyms
**HTG135200**	**Commercial Nematic Liquid Crystal hybrid**	
**C6M**	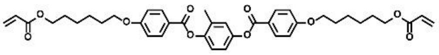	**2-methyl-1,4-phenylene bis(4-((6-(acryloyloxy)hexyl)oxy)benzoate))**
**R5011**	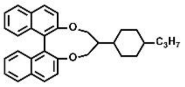	**(13bR)-5,6-Dihydro-5-(trans-4-propylcyclohexyl)-4H-dinaphtho[2,1-f:1',2'-h][1,5]dioxonin**
**TMPTA**	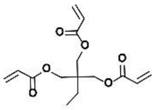	**1,1,1-Trimethylolpropane Triacrylate**
**I-651**	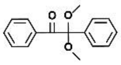	**2,2-Dimethoxy-2-phenylacetonphenone**
**Coumarin 6 (C6)**	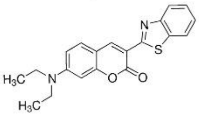	**3-(2-Benzothiazolyl)-N,N-diethylumbelliferylamine**

Supplementary Note 2 Basic optical properties of the phase transition process of BPLCs using POM

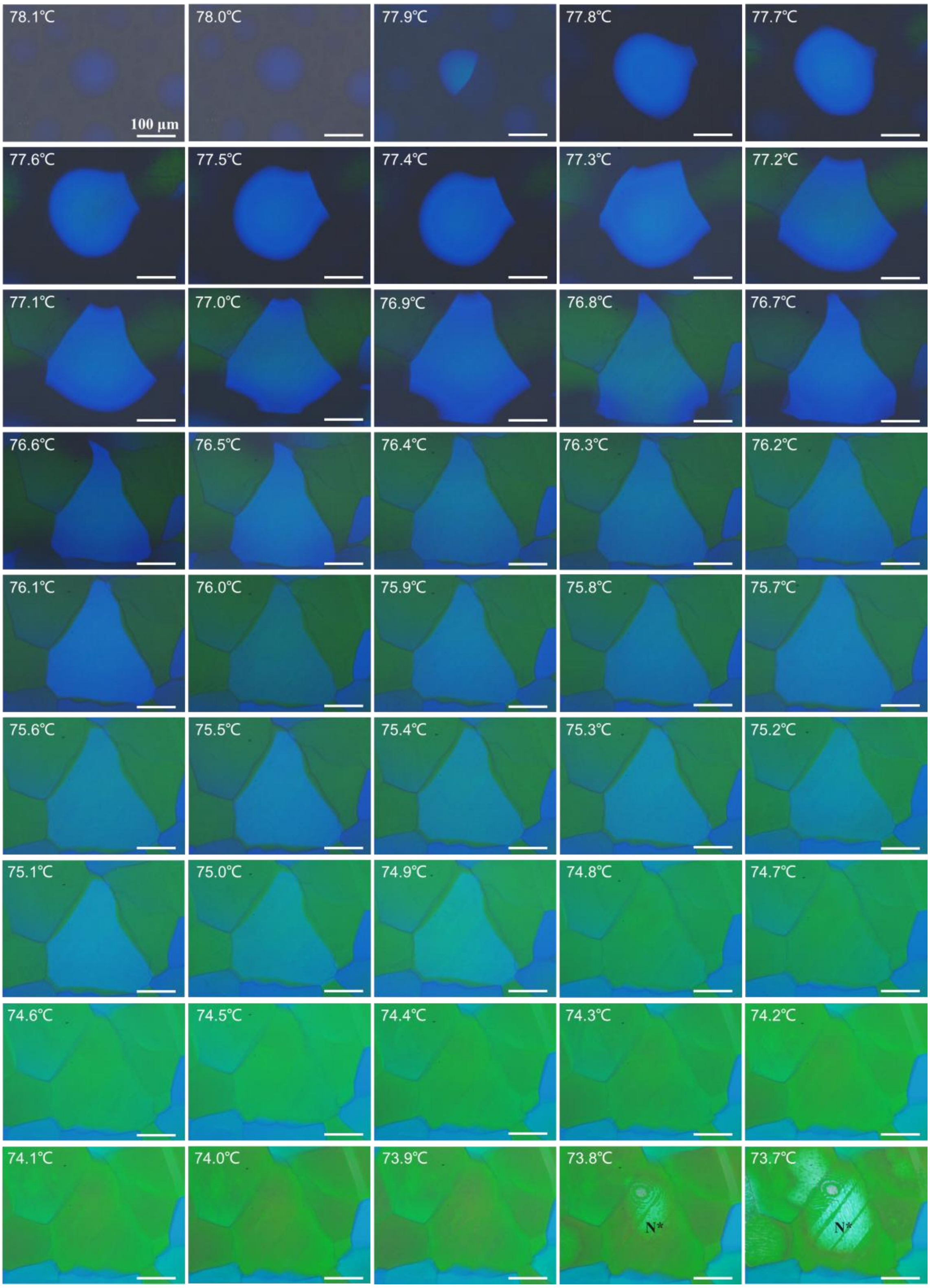

Supplementary Fig. 1 Temperature dependence of optical textures focused on one domain. Typical textures captured using POM for BPLCs during phase transformation from 78.1 to 73.7 ℃ (from Stage I to Stage IV). The temperature noted is from the bottom of the LC cell.

To gain additional insights into the phase transition process of BPLCs, detailed information was captured using POM focused on one domain of poly-domain BPLCs when cooling from 78.1 to 73.7 ℃ (from Stage I to IV) at a rate of 0.05 ℃/min. At 74.8 ℃, BPII disappears suddenly, and the transformation from BPI to the chiral nematic phase occurs at 73.7 ℃. The BPI temperature window T_{BPI} is 3.9 ℃ from 77.7 ℃ to 73.7 ℃ (without considering the coexistence of BPI/N*).

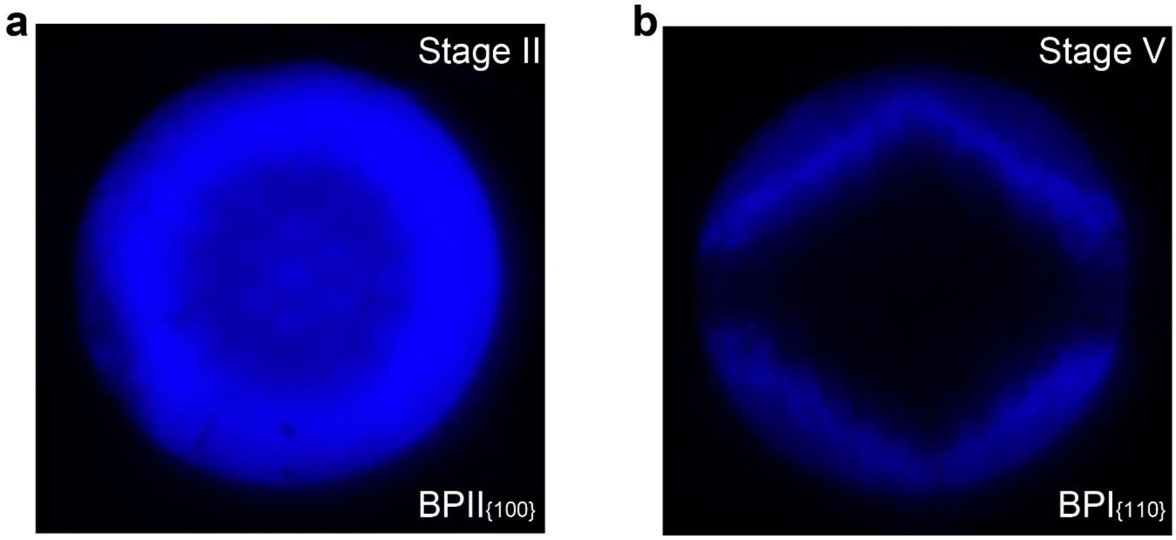

Supplementary Fig. 2 Kossel diagram of $BPII_{\{100\}}$ (the label of $BPII_{\{100\}}$ denotes that the BPII domain with {100} crystal planes parallel to the substrates) at Stage II and $BPI_{\{110\}}$ at Stage V.

In Stage II, BPII nucleates in BPIII forming BPIII/BPII core-shell configurations. The Kossel diagram shows a circle pattern along [100] direction of $BPII_{\{100\}}$ in BPIII/BPII core-shell configurations (a). In Stage V, all BPII and BPIII transfer to BPI. The Kossel diagram shows a diamond-shaped pattern along [110] direction of BPI (b).

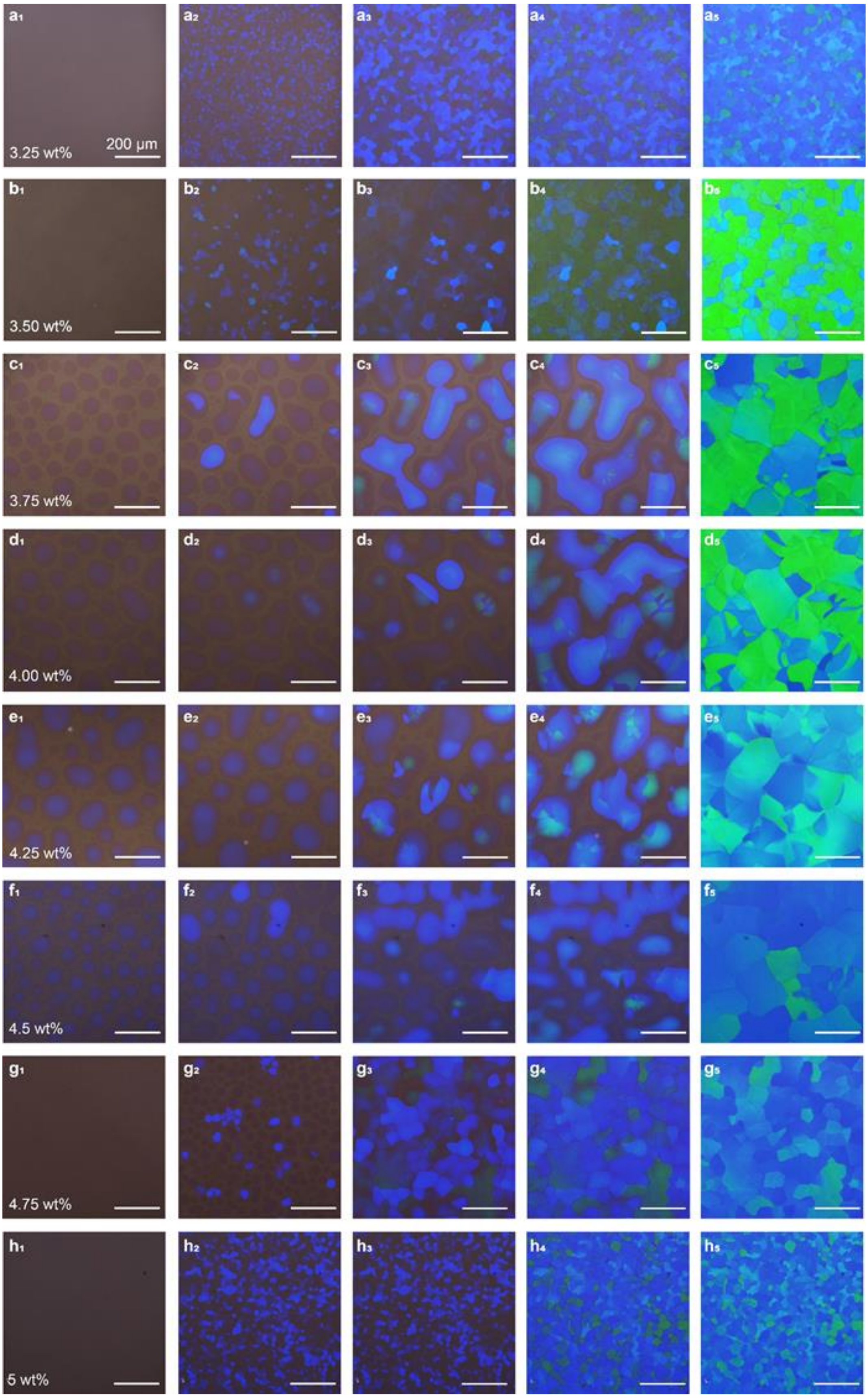

Supplementary Fig. 3 POM observation of phase transition behavior of BPLCs with a varyin g dosage of cross-linker: TMPTA. The dosage of TMPTA varies from 3.25 to 5.00 wt% where (**a_1-a_5**) 3.25 wt%, (**b_1-b_5**) 3.50 wt%, (**c_1-c_5**) 3.75 wt%, (**d_1-d_5**) 4.00 wt%, (**e_1-e_5**) 4.25 wt%, (**f_1-f_5**) 4.50 wt%, (**g_1-g_5**) 4.75 wt% and (**h_1-h_5**) 5.00 wt%.

Large-scale POM observation of phase transformation from BPIII to BPI during the cooling process from the isotropic state at 0.05℃/min is presented when varying the dosage of TMPTA. BPLCs with large domain sizes are obtained when the dosage of TMPTA is between 3.75 and 4.5 wt% which tends to form typical five stages (see Figure 1 in the main text). When the dosage of TMPTA is over 4.75 wt% or below 3.50 wt%, the size of the core-shell configuration is greatly reduced, resulting in the connection of BPIII/BPII core-shell configurations (a, g and h). In this case, no BPI can directly nucleate in the BPIII domain, resulting in the vanishing of BPIII/BPI core-shell configuration of Stage III.

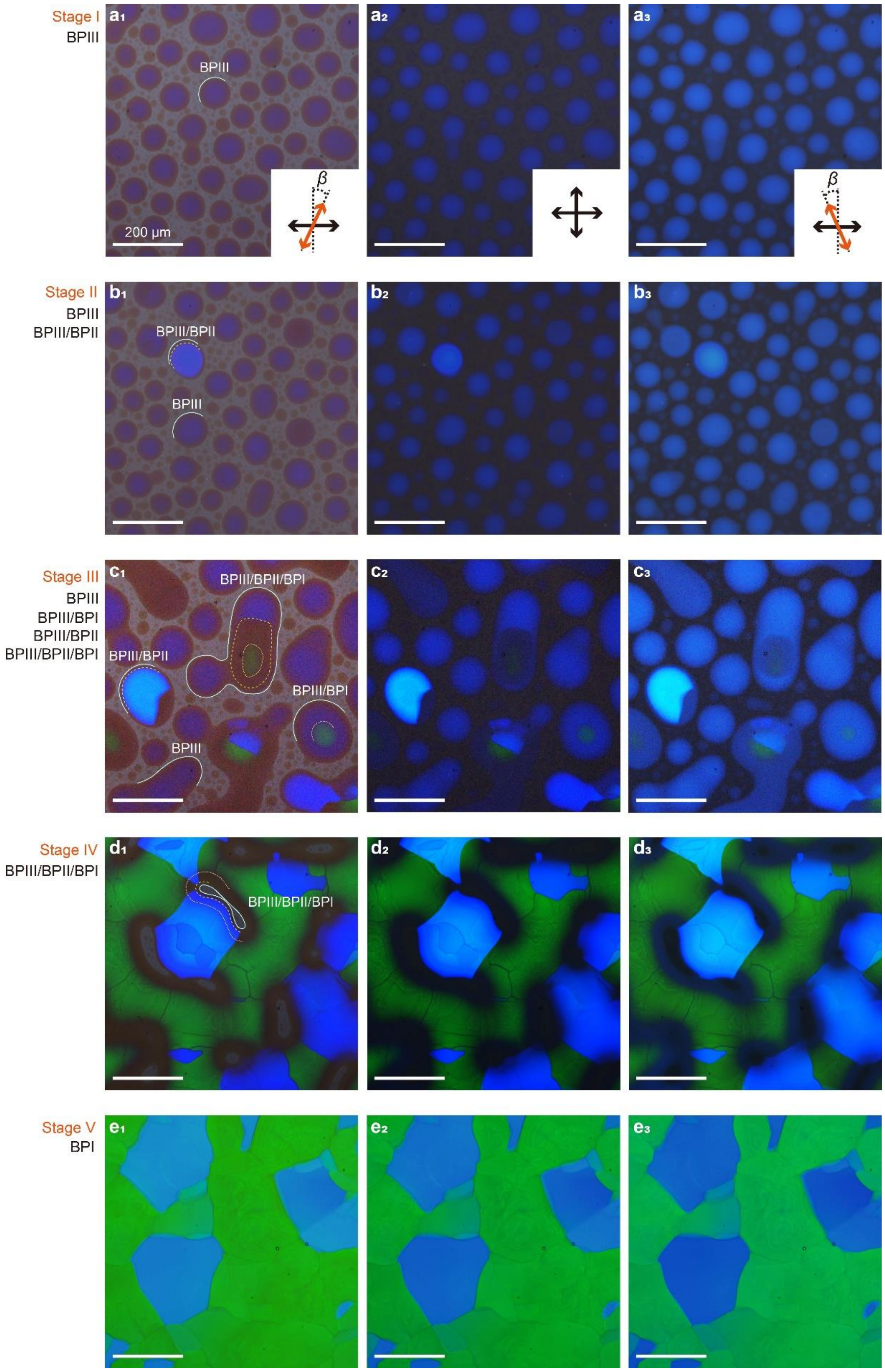

Supplementary Fig. 4 POM observation of core-shell configuration from each stage. POM images of ($\mathbf{a}_1$-$\mathbf{a}_3$) Stage I, ($\mathbf{b}_1$-$\mathbf{b}_3$) Stage II, ($\mathbf{c}_1$-$\mathbf{c}_3$) Stage III, ($\mathbf{d}_1$-$\mathbf{d}_3$) Stage IV, and ($\mathbf{e}_1$-$\mathbf{e}_3$) Stage V before polymerization. Arrows inserted in POM images of ($\mathbf{a}_1$-$\mathbf{a}_3$) represent the optic axes of polarizers and the deviation angle $\beta=\pm 4°$. The solid bright blue circle, the yellow dotted circle, and the orange dotted line in ($\mathbf{a}_1$-$\mathbf{d}_1$) highlight BPII, BPII, and BPI domains respectively.

According to previous works, BPIII could be conveniently distinguished from the isotropic state due to its optical activity which renders different colors (yellowish-brown and dark blue) when the analyzer of a POM is rotated slightly (deviation angle (β) of 4° is adopted here) to the left or right[1-3]. As shown in (a_1), the BPIII domain with dark blue color nucleates in the isotropic background during the cooling process. Dark blue is the typical color of BPIII in POM images with an analyzer rotated slightly left (a_1, b_1). In (b_1), the domain with dark blue is BPIII where the boundary of BPIII is labeled by a solid bright blue line. The domain of BPII is highlighted by a dotted yellow circle, which is covered by the dark brown shell of BPIII, forming a BPIII/BPII core-shell configuration. In (c_1), three kinds of core-shell configurations of BPIII/BPI, BPIII/BPII, and BPIII/BPII/BPI, as well as monophasic BPIII domain, are labeled. It is noteworthy that the brown domain circled by the yellow dash line in the BPIII/BPII/BPI core-shell configuration refers to the BPII with non {100} crystal plane parallel to substrates, while the bright blue domain highlighted by the yellow dash line in BPIII/BPII domain is BPII$_{\{100\}}$. It is convinced not only BPIII could be distinguished from the isotropic state but also BPII with non {100} crystal plane parallel to substrates could be distinguished from core-shell configurations by rotating analyzer of POM slightly to the left or right. In (d_1), All the core-shell configurations of BPIII/BPI and BPIII/BPII transfer to BPIII/BPII/BPI (Stage IV). In (e_1), all the brown and bright blue areas vanish, proving that BPIII and BPII completely transfer to BPI (Stage V).

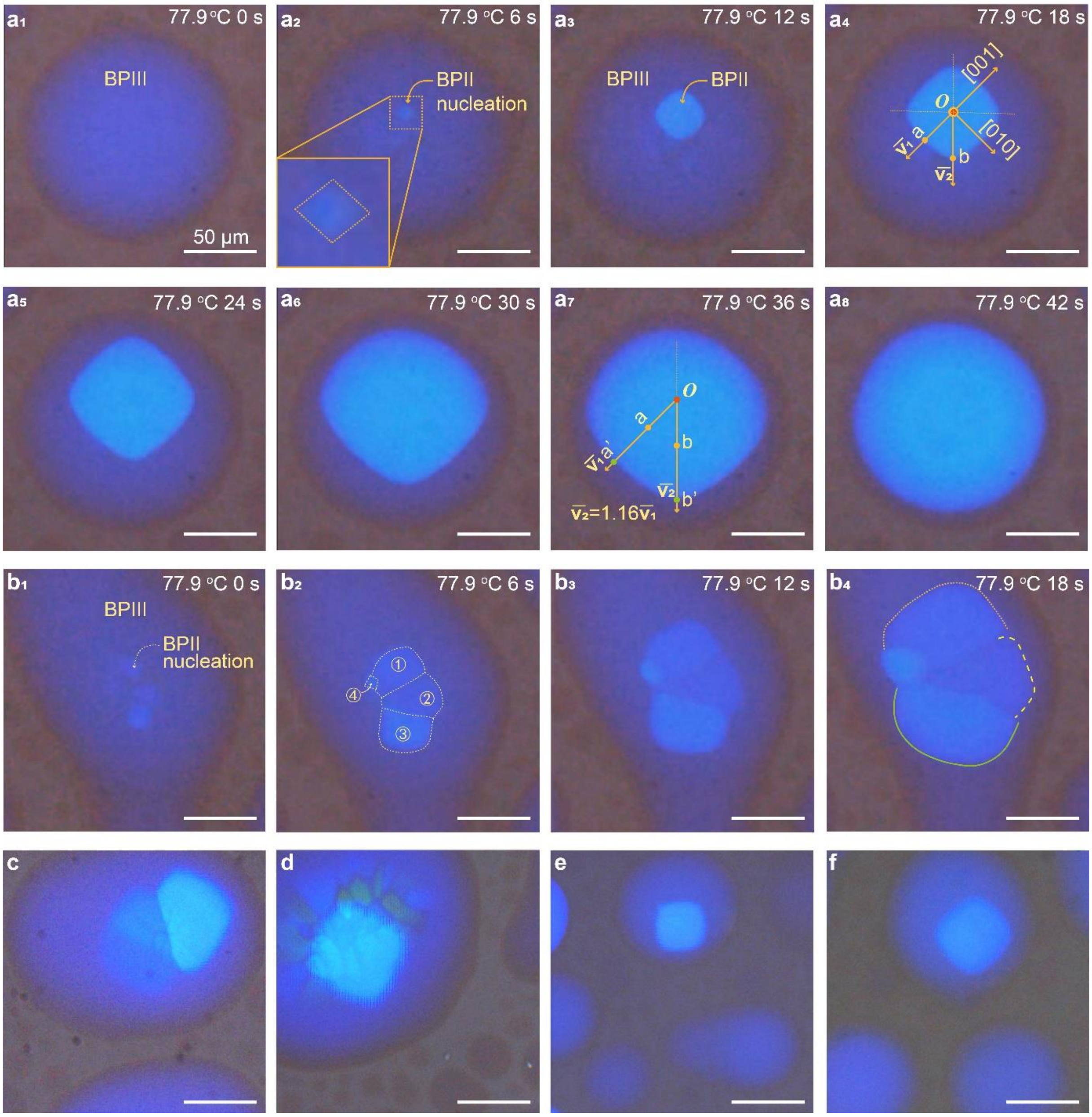

Supplementary Fig. 5 Nuclei and anisotropy growth of BPII in BPIII. a_1-a_8 One nuclei site of BPII in the BPIII domain. **b_1-b_4** Four nuclei sites of BPII in the BPIII domain. **c-f** Other examples of anisotropy growth of BPII in BPIII. The lines in B_2 and B_4 highlight the BPII grows from the four nuclei..

During the BPIII→BPII phase transition process, BPII nucleates in the center of BPIII as shown in (a_2) and (b_1). The nuclei of $BPII_{(100)}$ have a rounded square shape (inserted image of a_2). Then, $BPII_{\{100\}}$ grows up adiabatically and the growth rate is anisotropy where $\overrightarrow{\mathbf{v_2}}$ along [011] crystal direction (2.25 μm/s) is faster than $\overrightarrow{\mathbf{v_1}}$ along [001] crystal direction (1.93 μm/s), giving the relationship of $\overrightarrow{\mathbf{v_2}}=1.16\overrightarrow{\mathbf{v_1}}$. This relationship slightly derives from theoretical $\overrightarrow{\mathbf{v_2}}=\sqrt{2}\overrightarrow{\mathbf{v_1}}$ owing to the shape of nuclei is a rounded square. It is noticeable that the growth of

$BPII_{\{100\}}$ is not isotropy with a spherical shape which is predicted by simulation[4]. When there are more than one nuclei of $BPII_{\{100\}}$ in a BPIII domain (b_1-b_4), each nucleus grows anisotropically with a rounded square shape. More experiments further support that the anisotropy growth of $BPII_{\{100\}}$ in the BPIII domains (c-f). Besides, there is no BPII nucleus directly in the isotropic state without the pre-formed BPIII domain.

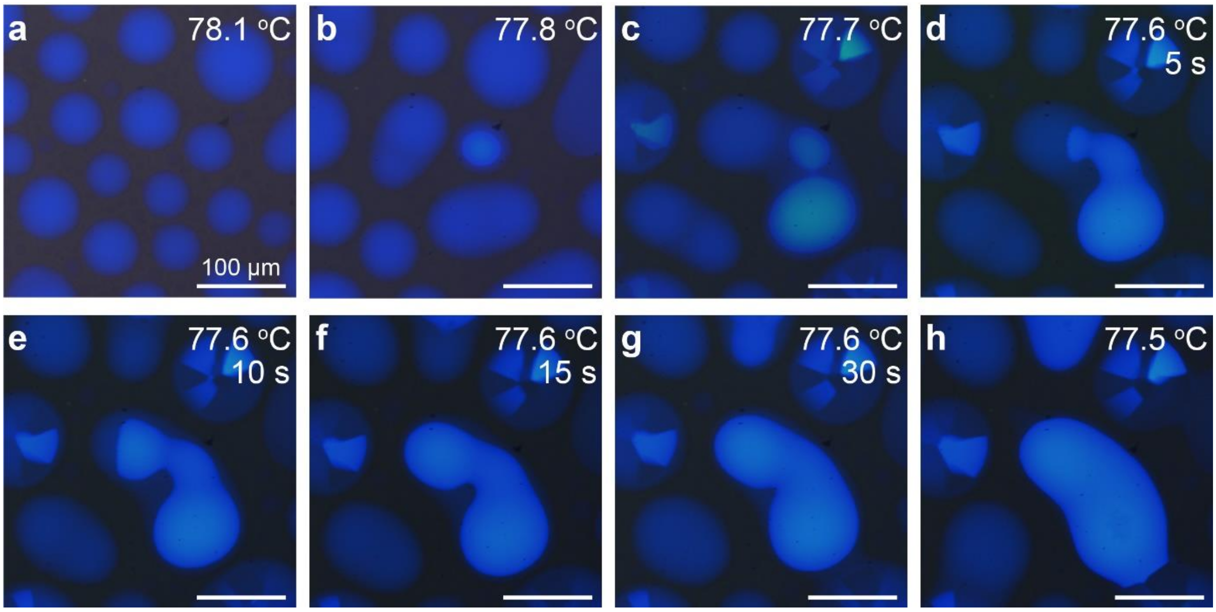

Supplementary Fig. 6 POM characterization of diffusionless phase transition (DLPT) from BPIII to BPII. a Typical textures of Stage I. **b, c** BPII nucleates in the center of the BPIII domain during the temperature reduction process. **d-g** Rapid growth with spreading behavior when the temperature is kept at 77.6 ℃ for 30 s. **h** BPII core and BPIII shell grow up spontaneously during the decreasing temperature process.

The phase transition from BPIII to BPII (equally to Stage I to II) is considered as a DLPT process where clear boundaries and spreading behavior are observed by POM. The textures were captured during temperature reduction from 78.1 to 77.5 ℃ at a cooling rate of 0.01 ℃/min, in which the rapid growth of BPII is observed with spreading behavior when the temperature is kept at 77.6 ℃ for 30 s. BPII starts to nucleate in the center of BPIII at 77.8 ℃ (b) and the number of BPII crystal nucleus increases during the temperature reduction process (c). When the temperature is kept at 77.6 ℃ for 30 s, the as-formed nuclei of BPII grow from the right-bottom part toward the left-up part (d-g), and the two nuclei merge into one BPII domain (c-d). The full size of the BPII domain is limited by the size of BPIII.

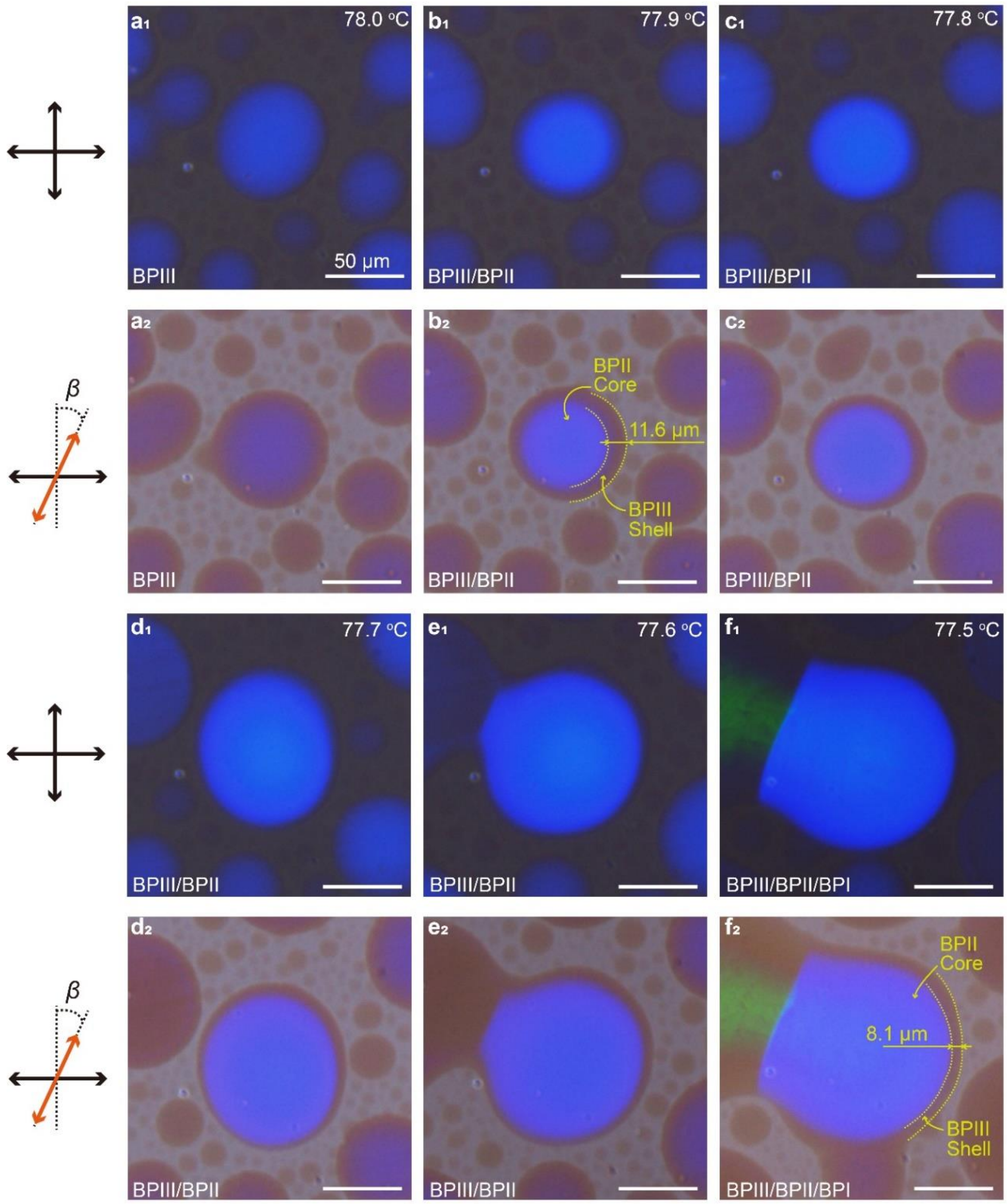

Supplementary Fig. 7 POM characterization of formation and evolution of BPIII/BPII core-shell configuration. POM images of (**a_1-a_2**) BPIII domain, (**b-e**) BPIII/BPII core-shell configuration, (**f_1-f_2**) Transformation from BPIII/BPII core-shell configuration to BPIII/BPII/BPI core-shell configuration. Red arrows on the left of POM images represent the optic axes of polarizers and the deviation angle $\beta = \pm 4°$.

After the formation of BPIII/BPII core-shell configuration through nuclei and anisotropy growth process (Supplementary Fig. 5), the domain with BPIII/BPII core-shell configuration grows up spontaneously during the cooling process (b-e). Notably, the ratio between BPIII and BPII has changed, that is BPIII shell gradually becomes thinner from 11.6 μm (b) to 8.1 μm (f).

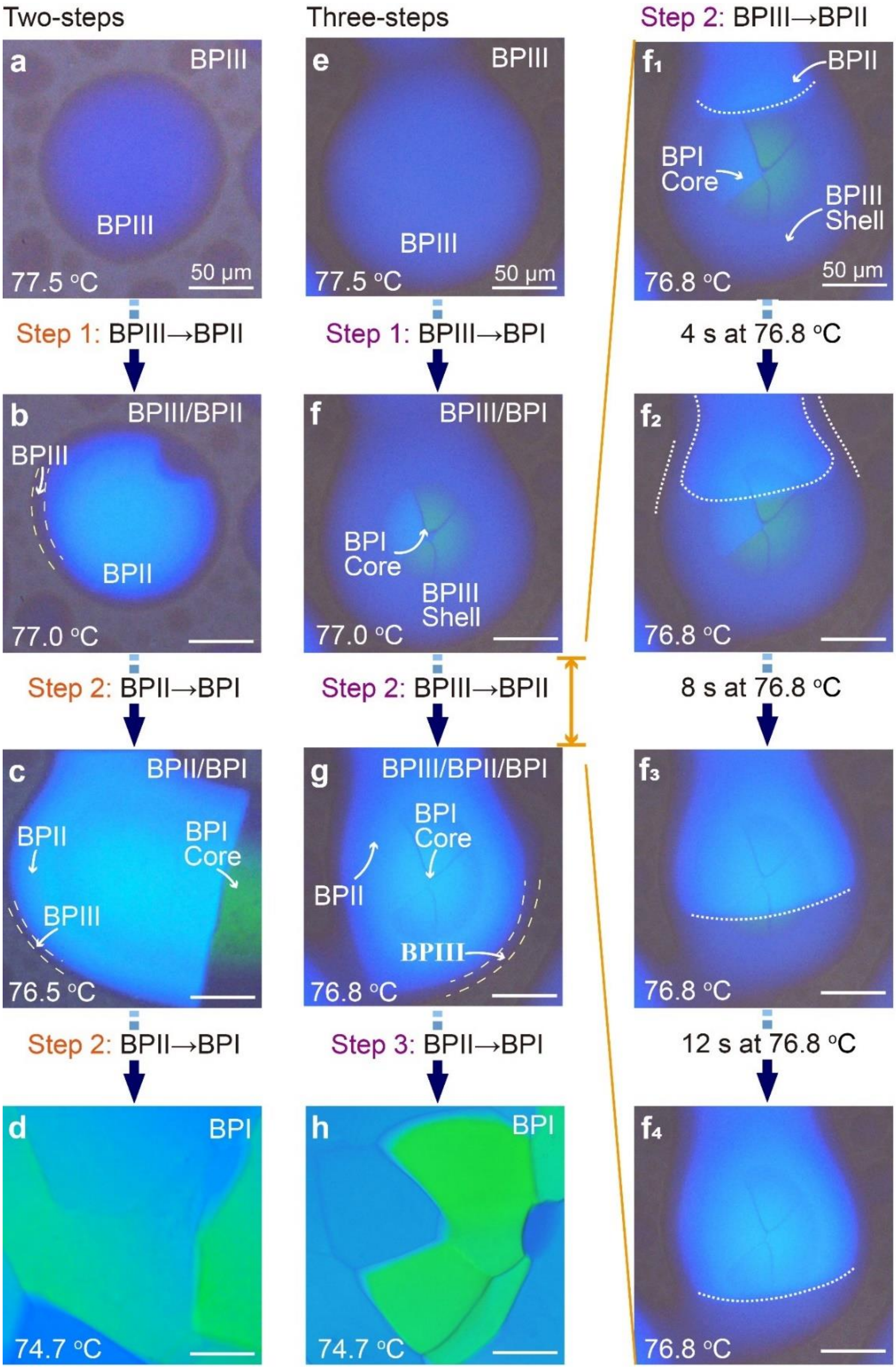

Supplementary Fig. 8 POM characterization of the two-, three-step successive DLPT of BPLCs. a-d Two-step process: (**a**) BPIII domain in Stage I, (**b**) BPIII/BPII core-shell configuration in Stage II & III obtained by DLPT from BPIII to BPII, (**c**) BPIII/BPII/BPI core-shell configuration in Stage III & IV, and (**d**) polydomain BPI in Stage V obtained by thermoelastic martensitic transformation from BPII to BPI. e-h Three-step process: (**e**) BPIII domain in Stage I, (**f**) BPIII/BPI core-shell configuration in Stage III obtained by DLPT from BPIII to BPI, (**g**) BPIII/BPII/BPI core-shell configuration in Stage III & IV obtained by DLPT from BPIII to BPII and (**h**) polydomain BPI in Stage V obtained by thermoelastic martensitic transformation from BPII to BPI. **f_1-f_2** Detailed characterization of BPIII→BPII transformation from BPIII/BPI to BPIII/BPII/BPI core-shell configuration.

During phase transition from Stage I to V, there exist three kinds of core-shell configurations: (i) BPIII/BPII configurations (BPII covered by a thin shell of BPIII) (b); (ii) BPIII/BPII/BPI core-shell configurations (BPI covered by BPII and BPIII sequentially) (c, g); (iii) BPIII/BPI core-shell configurations (BPI covered by BPIII) (f).

In BPLCs, BPIII-to-BPII, BPIII-to-BPI, and BPII-to-BPI are DLPT processes, in which two- or three-step successive phase transformation is observed. In a two-step process (a-d), BPLCs firstly form a BPIII/BPII core-shell configuration in Stage II (Step 1: DLPT of BPIII→BPII), and then BPI nucleates and grows in BPII in Stage III forming a BPIII/BPII/BPI core-shell configuration (Step 2: the thermoelastic martensitic transformation of BPII-to-BPI).

In a three-step process, BPI firstly nucleates in the center of the BPIII domain in Stage III (Step 1: DLPT of BPIII-to-BPI), then part of BPIII shell transfers to BPII (Step 2: DLPT of BPIII-to-BPII). BPI core grows with the decreasing temperature until BPII completely transfers to BPI (Step 3: the thermoelastic martensitic transformation of BPII-to-BPI).

This two-step successive DLPT is rarely investigated in soft matter or atomic crystals.

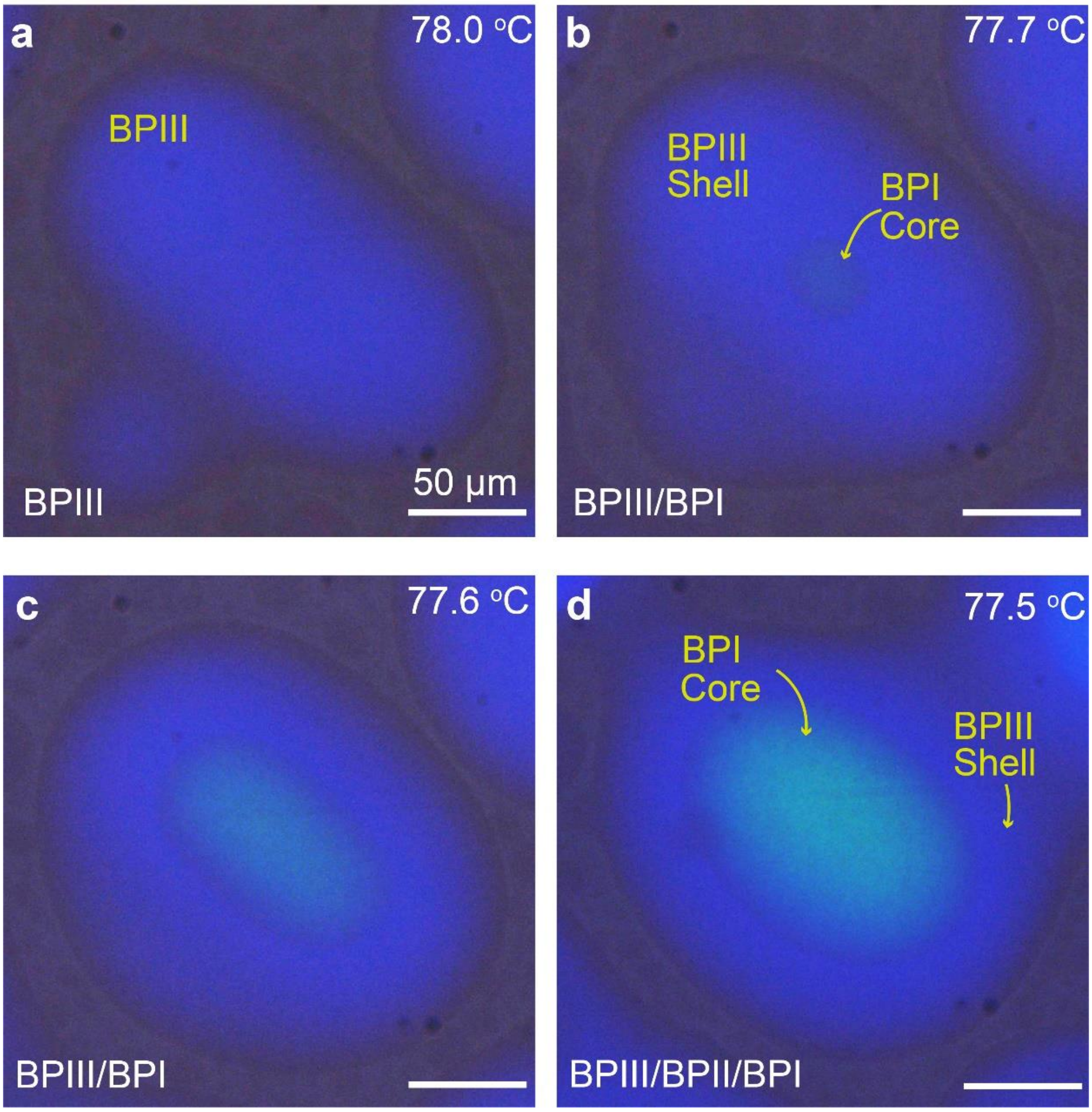

Supplementary Fig. 9 POM characterization of the formation process of BPIII/BPI core-shell configuration. a BPIII domain. **b-d** Growth of BPI in BPIII toward the formation of BPIII/BPI core-shell configuration.

In this work, BPI can transfer from BPIII directly without the pre-formed BPII, forming a BPIII/BPI core-shell configuration (b-d). It is found that this process is widely observed in polydomain BPLCs with a large domain size of over 150 μm. The direct phase transformation from BPIII to BPI is observed earlier than the transformation from BPIII to BPII.

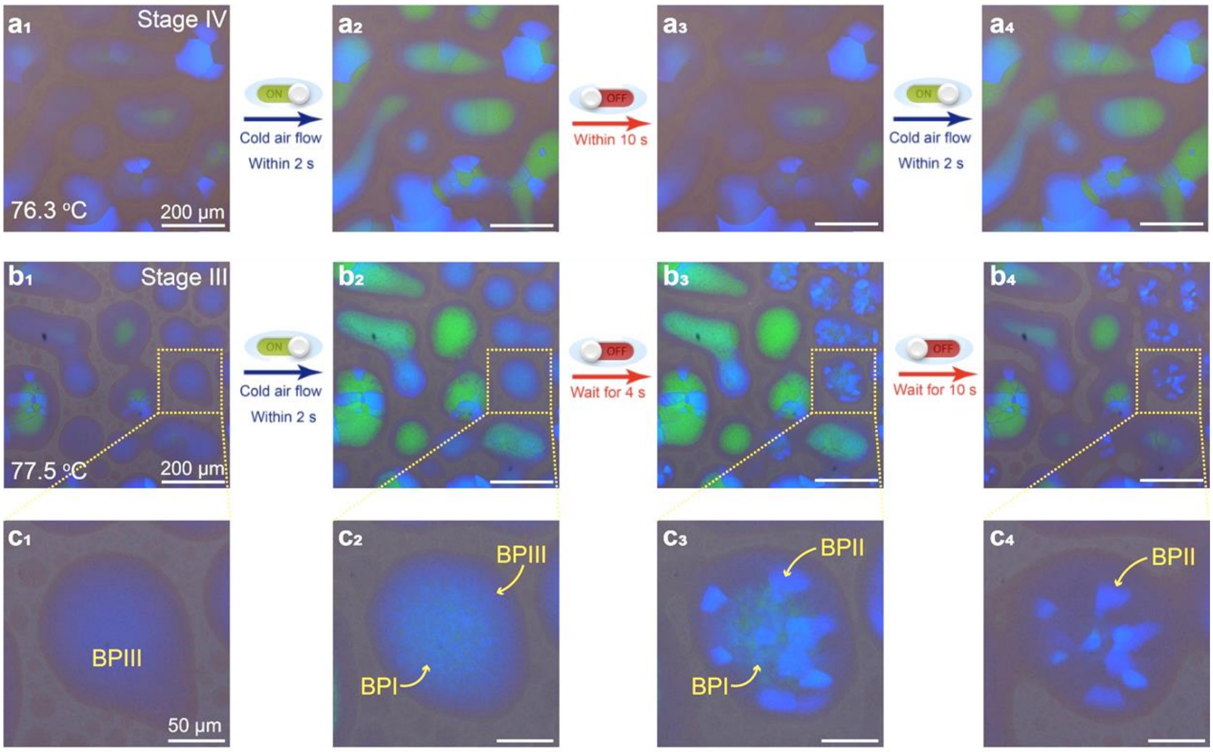

Supplementary Fig. 10 POM observation of speed and reversibility of DLPT between BPI and BPII at Stage III or IV. a_1-a_4 Textures of Stage IV with the temperature of the hot stage at 76.3 °C upon flowing or stop flowing color air for the same region. **a_1** The original state of Stage IV. **a_2** A phase transition occurs within 2 s from BPII to BPI after flowing cold air. **a_3** The BPI in BPIII/BPII/BPI core-shell configuration transfer back to BPII after stopping flowing cold air for 10 s. **a_4** The phase transition from BPII in BPIII/BPII/BPI core-shell configuration to BPI within 2 s upon flowing cold air. **b_1-b_4** Textures of Stage III when the temperature of the hot stage is kept at 77.5 °C with or without flowing or stop flowing cold air. **b_1** The original state of Stage IV. **b_2** The phase transition from BPIII domain to BPIII/BPII core-shell configurations or from BPII in BPIII/BPII/BPI core-shell configurations to BPI after flowing cold air within 2 s. **b_3** The BPII appears between the BPIII and BPI in BPIII/BPI core-shell configurations in **b_2** after stopping flowing cold air for 4 s. **b_4** The BPI in BPIII/BPII/BPI core-shell configuration transfer back to BPII after stopping flowing cold air for 10 s. **c_1-c_4** Enlarged images of the area highlighted by yellow dotted squares in **b_1-b_4**.

The DLPT between BPII to BPI is rapid and completely reversible which are the two significant characteristics of thermoelastic martensitic transformation (a_1-a_4). Herein, the transformation from BPII in BPIII/BPII/BPI core-shell configurations to BPI takes place within 2 s when the temperature of the upper surface of the LC cell is slightly reduced by flowing cold air. Reversibly, the transformation from BPI in BPIII/BPII/BPI core-shell configurations to BPII takes place within 10 s when the temperature of the upper surface of the LC cell recovers to the original state by stopping flowing cold air. It demonstrated high reversibility with more than 50 conversion-reversion cycles (Movie 2), indicating little temperature hysteresis for the DLPT between BPII and BPI. In (b_1-b_2), the framed part of the POM image is BPIII domains,

and transformation from BPIII domains to BPIII/BPI core-shell configurations take place within 2 s when the temperature of the upper surface of the LC cell is slightly reduced by flowing cold air (b_2), BPI appears in the center of BPIII. After stopped cold air for 4 s, the BPII appears between the BPIII shell and BPI core, resulting in all the BPIII/BPI core-shell configurations convert to BPIII/BPII/BPI core-shell configurations (b_3). Finally, BPI cores in BPIII/BPII/BPI core-shell configurations transfer to BPII (b_4) after stopping flowing cold air for 10 s. It is confirmed that the phase transformation between BPIII and BPI is not completely reversible with thermal hysteresis and the transformation between BPII and BPII is completely reversible with little hysteresis.

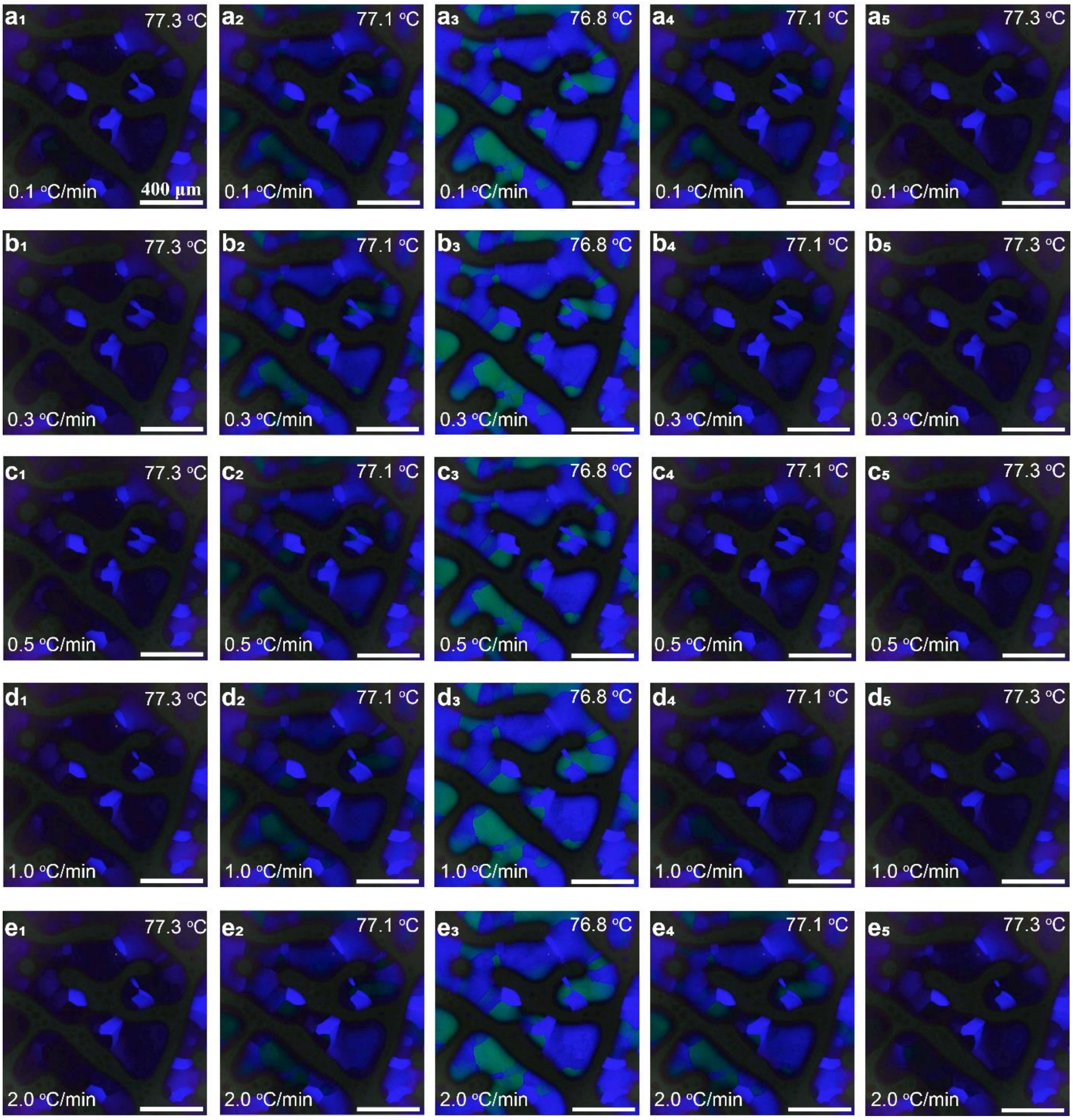

Supplementary Fig. 11 Reversible thermoelastic martensitic transformation between BPII and BPI in BPIII/BPII/BPI core-shell configurations (Stage IV) with distinct temperature change rates of (**a**) 0.1, (**b**) 0.3, (**c**) 0.5, (**d**) 1.0, and (**e**) 2.0 °C/min

Except for flowing the cold air over the sample, several detailed heating and cooling cycles are performed on the BPLCs at the rates of 0.1, 0.3, 0.5, 1.0 to 2.0 °C/min to investigate the phenomenon of hysteresis as clear as possible. The temperature is controlled by a heating stage equipped with a liquid nitrogen cooling system. The phase transition between BPI and BPII is proved without obvious temperature hysteresis, providing evidence of thermoelastic martensitic transformation.

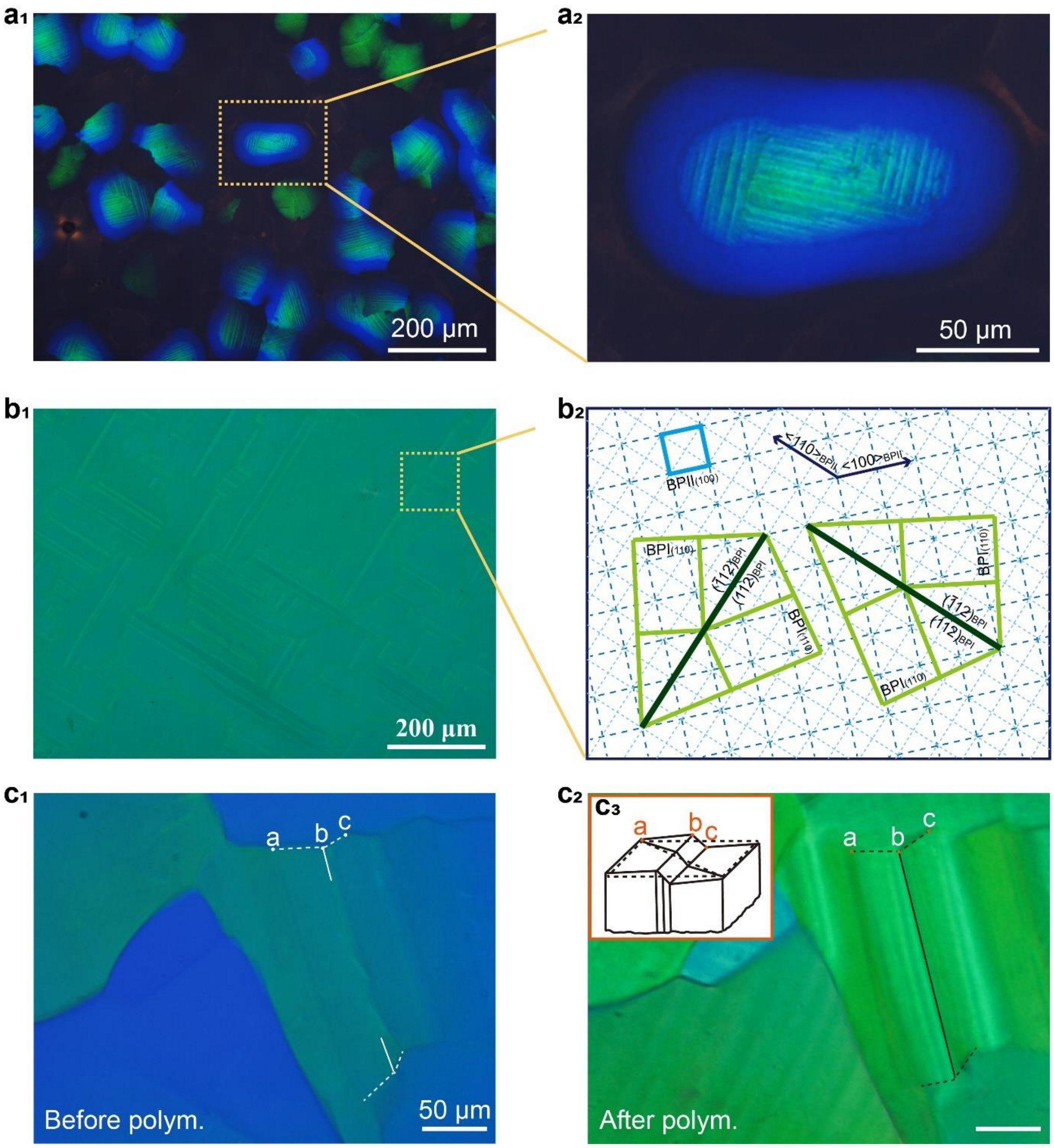

Supplementary Fig. 12 Twins (**a-b**) and surface reliefs (**c**) observed during the phase transformation. **a_1** POM image taken in reflection mode. The phase transition from polydomain BPII to BPI occurs in an LC cell composed of two bare glass substrates without any surface treatment. Cross-hatched textures, namely twin textures, were observed in the center of the BPIII/BPII/BPI core-shell configurations, where $BPI_{\{110\}}$ was covered by $BPII_{\{100\}}$ and then BPIII. **a_2** Magnified POM image exhibiting the twin textures in polydomain BPLCs. **b_1** Twin textures observed in a monodomain $BPI_{\{110\}}$ transferred from monodomain $BPII_{\{100\}}$. **b_2** Schematic of the $BPI_{\{110\}}$ lattice after martensitic transformation from monodomain $BPII_{\{100\}}$. **c_1-c_2** Surface reliefs of $BPI_{\{110\}}$ after phase transformation from $BPII_{\{100\}}$. **c_1** before and **c_2** after polymerization. **c_3** Schematic of the surface reliefs. Before polym.' and 'after polym.' refers to before polymerization and after polymerization

It is observed that the cross-hatched textures of BPI exhibit in both poly- (a_1-a_2) and single-domain BPLCs (b_1-b_2), originating from a thermoelastic martensitic transformation from the $BPII_{\{100\}}$. As shown in (a_1-a_2), the cross-hatched textures appear in a BPIII/BPII/BPI core-shell

configuration, in which $BPI_{\{110\}}$ (green) is covered by a $BPII_{(100)}$ (blue). The cross-hatched textures are observed in polydomain $BPI_{\{110\}}$ (a_2) which transfer from $BPII_{\{100\}}$ (a_1). These textures are the results of strain release via twinning which is anticipated for martensitic transformation. A similar texture is observed in monodomain $BPI_{\{110\}}$ (b_1), in which the bands are larger than their polydomain counterparts (a_1-a_2). The alternating lamellar of the two possible configurations (b_1) is caused by the twinning corresponding to the monodomain $BPI_{\{110\}}$ with equal proportions. The idealized arrangement of the two twin sets framed in (b_1) is shown in (b_2), and $\{112\}_{BPI}$ is parallel to $\{110\}_{BPII}$ which is further confirmed by syn-SAXS (Supplemental Fig. 52). The cross-hatched textures appear due to the reduction of the average macroscopic strain of the unit cell, and the shear strain cannot be resolved through the formation of a single twin, and each twin exhibits a shear strain of the opposite direction. These neighboring bands are considered to be two variants that together relax the transformation strains (b).

In addition, the surface reliefs (c_1-c_3) in $BPI_{\{110\}}$ before (c_1) and after (c_2) polymerization, as characteristic properties of martensitic transformation, are observed due to the shear strain during the phase transformation from $BPI_{\{110\}}$ to $BPII_{\{100\}}$. The grain consists of parallel bands and the adjacent bands are parallel to $\{112\}_{BPI}$ (c_1-c_3).

The results of twinning and surface reliefs further confirm the argument that the phase transformation between BPII and BPI takes place in a diffusionless manner.

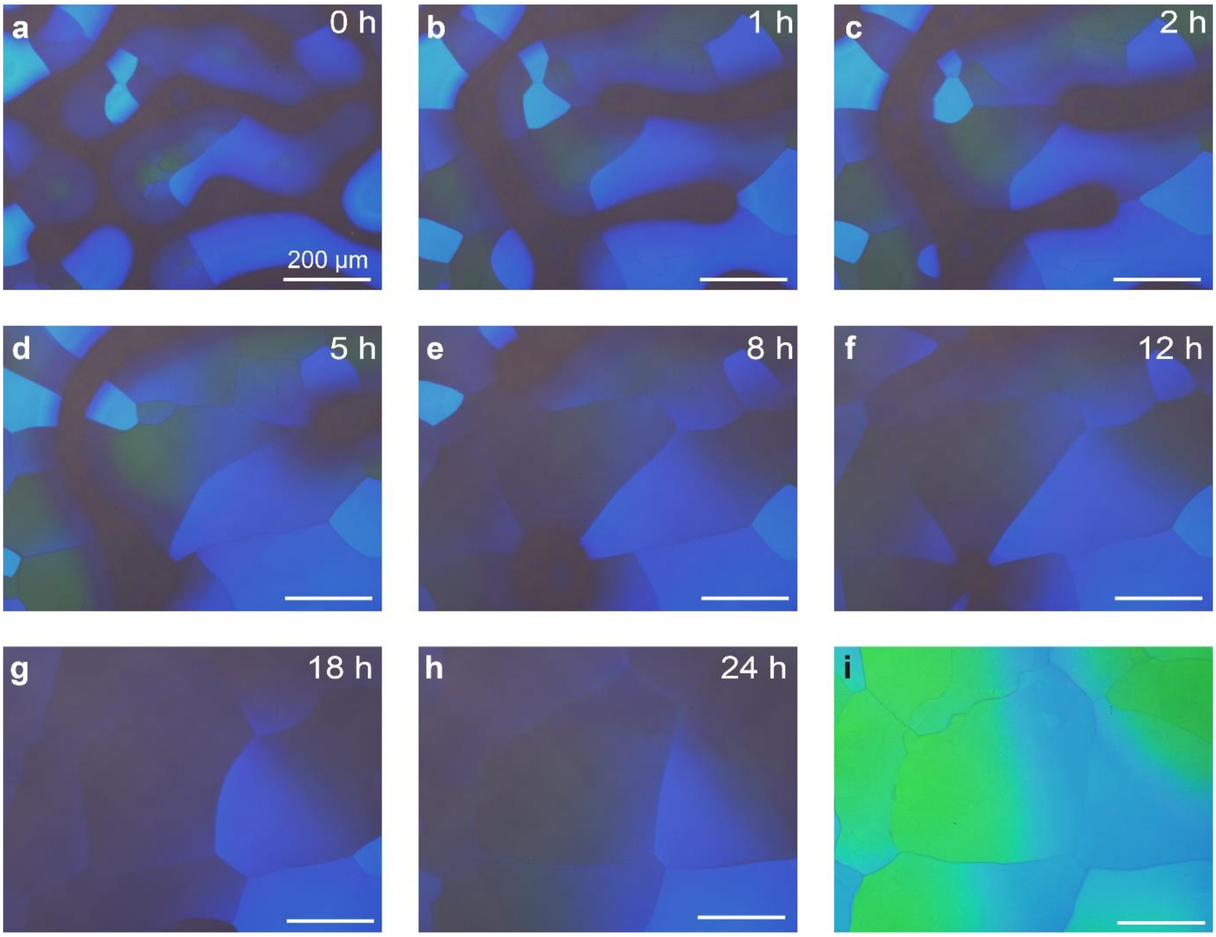

Supplementary Fig. 13 The enlargement of domain size of BPLCs at Stage IV annealed with increasing time. a Optical textures of fresh-formed BPLCs by direct cooling from the isotropic state to 76.2 ℃ (Stage IV) at 0.05 ℃/min. **b-h** Optical textures with the temperature maintained at 76.2 ℃ after being preserved for 1-24 h. **i** BPI obtained by cooling from (**h**) at a cooling rate of 0.05 ℃/min to 74.7 ℃.

It is observed that the number of nuclei both BPI and BPII in BPIII/BPII/BPI core-shell configurations is gradually reduced (a-h) after annealing for 1-24 h at 76.2 ℃. providing the possibility for enlarging the domain size of BPLCs which is considered as a significant issue to improve the performance of the devices fabricated based on BPLCs.

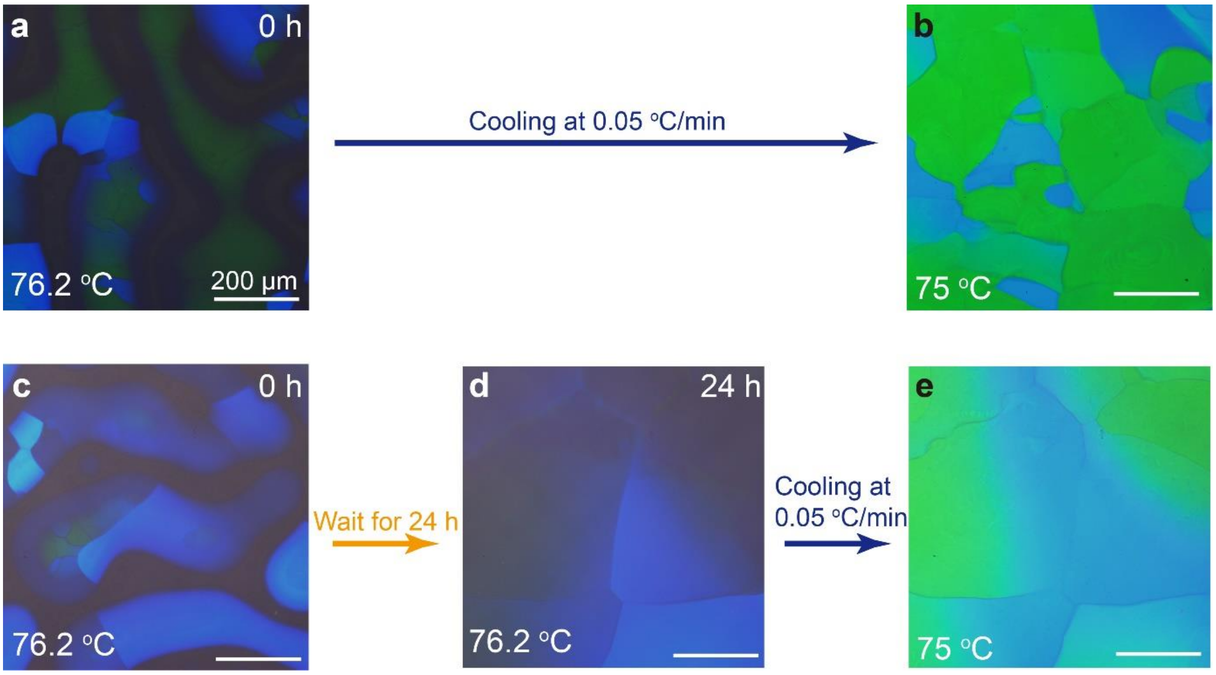

Supplementary Fig. 14 Enlargement of domain size by annealing. a,c BPLCs in Stage IV (BPIII/BPII/BPI) cooled from the isotropic phase to 76.2 ℃. **b** BPI obtained by further cooling the sample from 76.2 ℃ (a) to 74.7 ℃ at 0.05 ℃/min. **d** Texture mixed with BPI and BPII obtained after maintaining the temperature for 24 h. **e** BPI with domain-size-enlarged BPLCs obtained by cooling from (**d**) at 0.05 ℃/min.

It is found that the ultra-large domain size of polydomain BPI (d-e) can be obtained by reduction of the number of nuclei (Supplementary Fig. 13) which is achieved by annealing for 24 h at 76.2 ℃ (c-d). Polydomain BPI with a large domain size over 1 mm (e) is obtained after slowly cooling from the enlarged BPIII/BPII/BPI core-shell configurations (d) at 0.05 ℃/min. The BPI domain is nearly 3 times (e) larger than that without annealing (b).

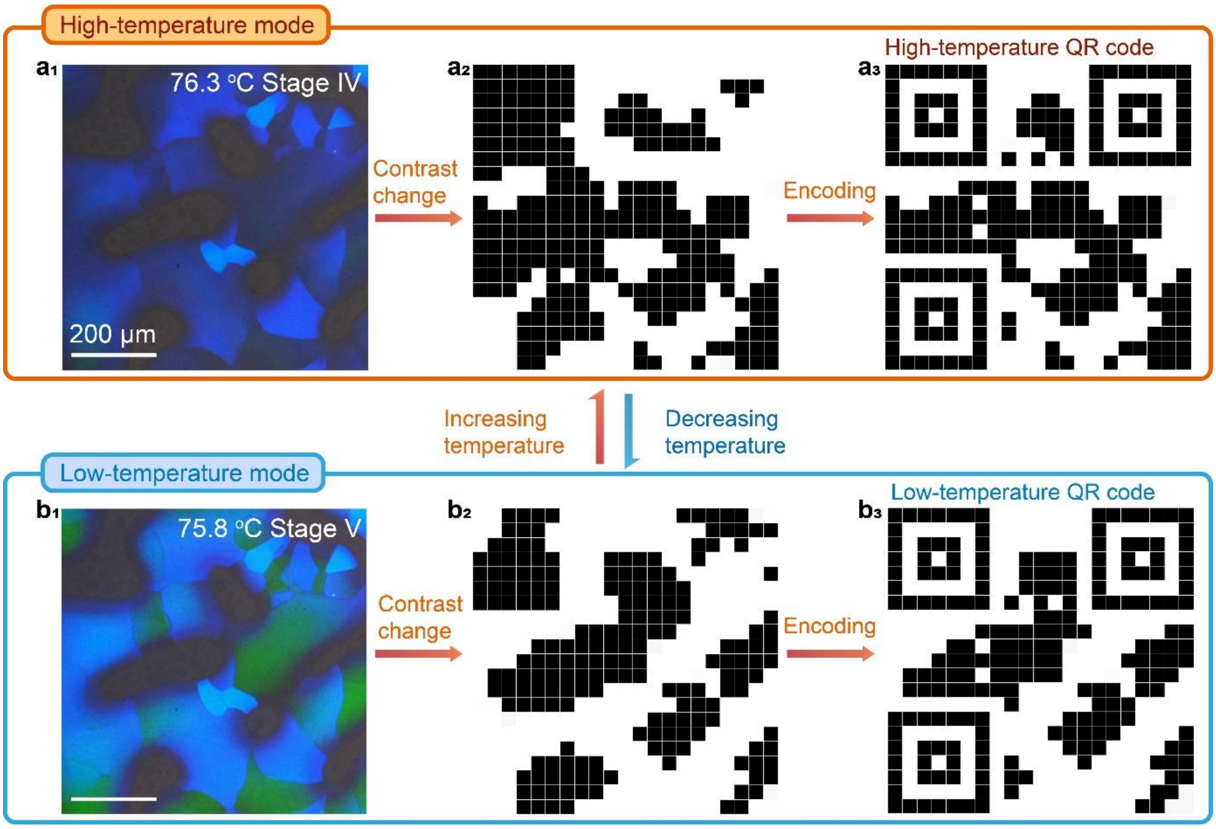

Supplementary Fig. 15 Temperature-switchable QR codes. a_1-a_3 High-temperature QR code. **a_1** The POM image of BPLCs at Stage IV was captured at 76.3 °C. **a_2** Image obtained after gray-level conversion, contrast change, and pixelation processes from (**a_1**) according to the image brightness. **a_3** A QR code with a size of 21×21 is encoded based on (**a_2**). **b_1-b_3** Low-temperature QR code. **b_1** POM image of BPLCs Stage IV was captured at 75.8 °C. **b_2** Image obtained from (**b_1**) according to the image brightness. **b_3** A QR code with a size of 21×21 is encoded based on (**b_2**).

Due to the rapid and completely reversible properties of thermoelastic martensitic transformation between BPII and BPI, the reversible switching of POM images are achieved at high and low temperatures (a_1, b_1). This reversible storage of patterns in soft BPLCs can be considered as an analogy to the shape-memory in solid-solid crystal transformation. The temperature-switchable QR codes undergo the processes (Supplementary Figs. 4a-b) of gray-level conversion, contrast change, pixelation (a_2, b_2), and encoding (a_3, b_3). Thus, the POM and corresponding QR codes obtained at high-temperature mode (76.3 °C) with particular information are unique in different samples or cooling circles. During the fast cooling process from 76.3 °C to 75.8 °C at 1 °C/min, the BPI in BPIII/BPII/BPI core-shell configurations grows up fast and leads to an increase of brightness and changing of colors of the POM image (b_1). A coupled QR code is obtained (b_2-b_3) at low-temperature. When the sample is heated from 75.8 °C

to 76.3 ℃, a completely reverse transformation from BPI in BPIII/BPII/BPI core-shell configurations to BPII occurs by gradual reduction of the existing BPI. Finally, almost all BPI are converted into BPII and the distribution of core-shell configurations is recovered to the original high-temperature mode, resulting in the recovered QR code is the same as the initial one obtained before the first cooling circle. Thus, a pair of QR codes achieved at high/low temperatures can be switched reversibly with high duration stability (Movie 2). The switchable QR code has higher security than static QR code.

Supplementary Note 3 Thermal stability of PS-BPLCs

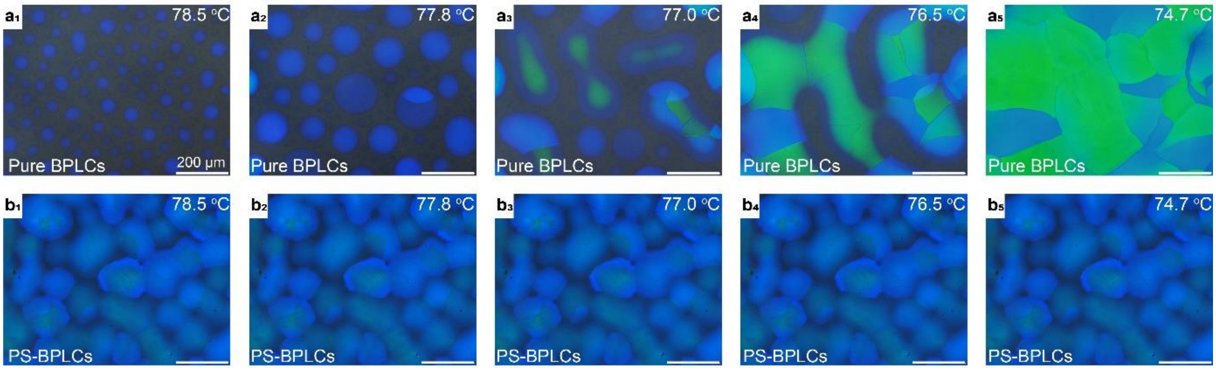

Supplementary Fig. 16 Textures varied with temperature when cooled from 80.0 °C (the isotropic state for BPLCs) to 74.7 °C (BPI, StageV) at a rate of 0.05 °C/min. a_1-a_5 BPLCs exhibit DLPT properties. **b_1-b_5** PS-BPLCs exhibit no changes.

The BPLCs that have DLPT properties are not photopolymerized (a_1-a_5) which sequentially show the typical five stages. However, the mixed liquid crystal component contains reactive monomers and initiators, which can be photopolymerized to stabilize the structures of coexisted BPLCs for detailed investigation of DLPT. Once the BPLCs are photopolymerized, the PS-BPLCs in obtained which the phase transition process cannot occur (b_1-b_5).

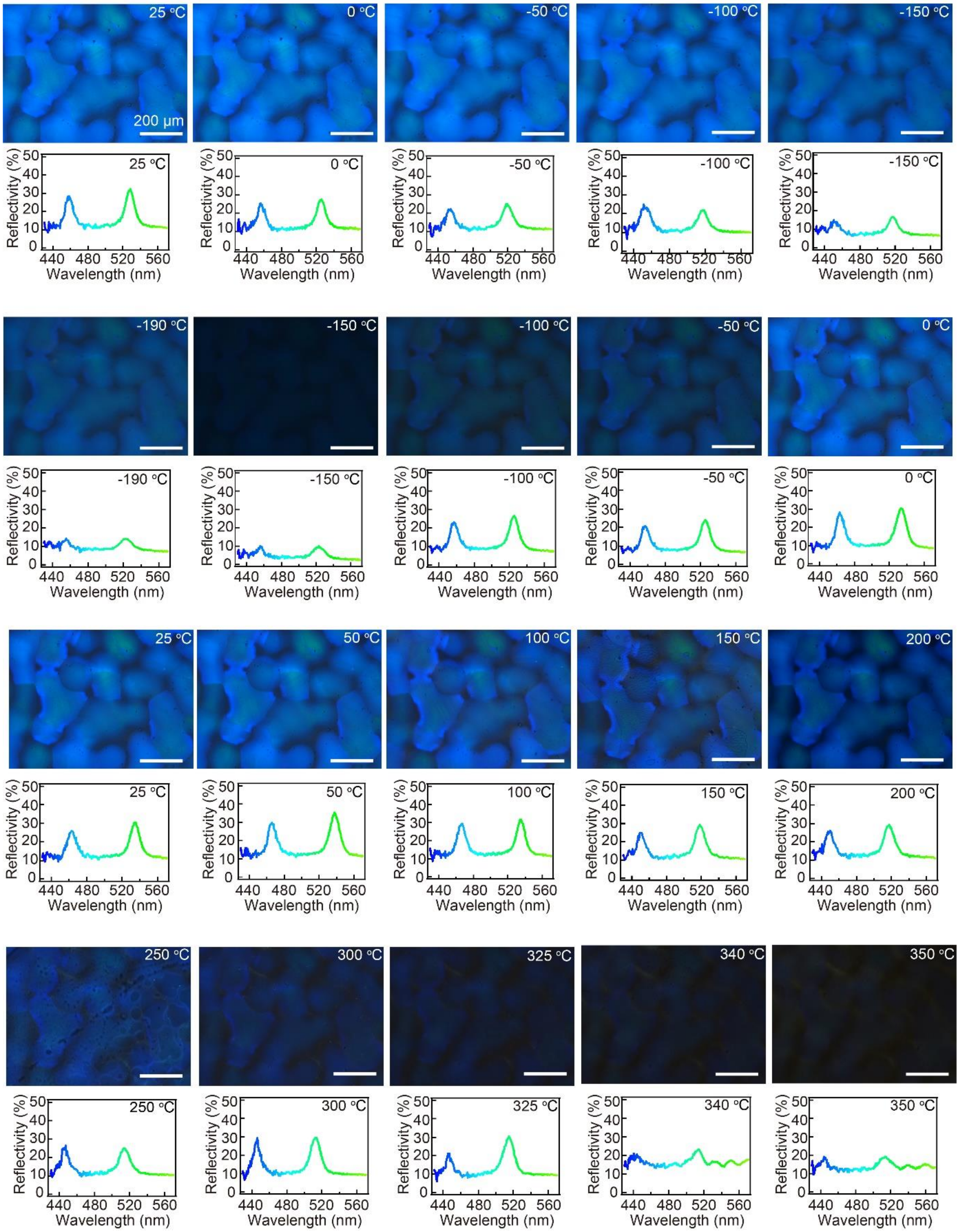

Supplementary Fig. 17 Thermal stability of PS-BPLCs in Stage IV (BPIII/BPII/BPI core-shell configuration). POM images indicate the range of thermal stability of PS-BPLCs in Stage IV, from -190 to 350 °C. The corresponding reflection spectra are shown behind the POM images.

In this work, the temperature window of PS-BPLCs is identified by firstly cooling the samples from 25 to -190 °C at a cooling rate of 5 °C/min and then heating them to 350 °C at a heating rate of 5 °C/min. Temperature-dependent POM images and reflection spectra of the

sample in Stage IV are shown. The reflectivity is reduced at the lower (close to -190 °C) or higher ends (close to 350 °C) of this range, suggesting a gradual transition to the isotropic state at high temperatures, and a possible glass transition at low temperature. A small percentage of reflectance reduction is caused by the increase of scattering from distinct polymer networks. Little change of texture brightness is observed by POM.

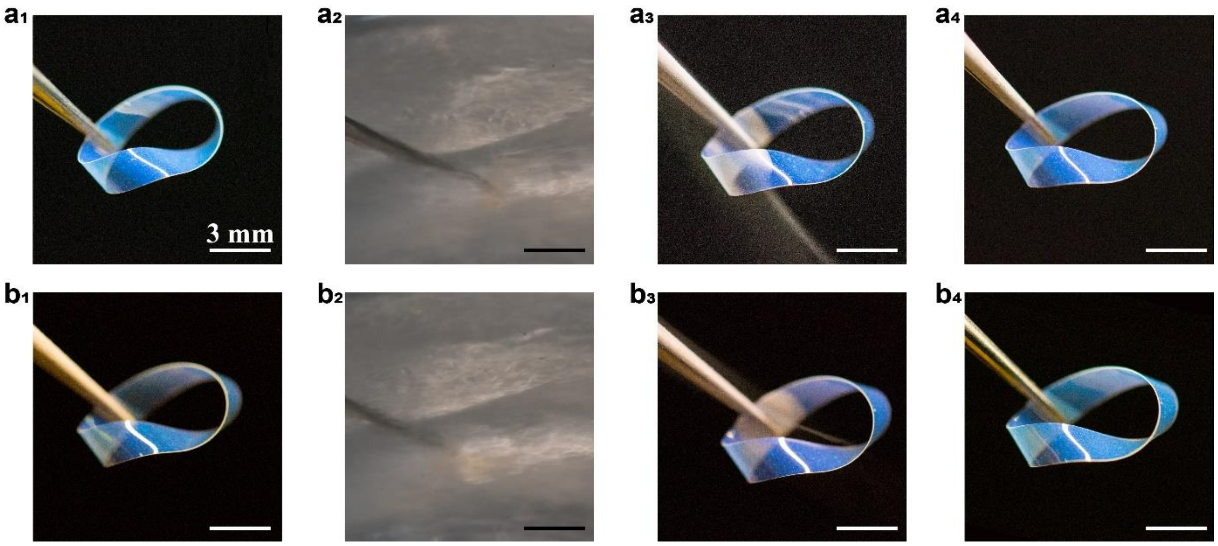

Supplementary Fig. 18 Low-temperature stability of PS-BPLCs at Stage IV during 10 temperature circles. a_1-a_4 Photographs of the Mobius strip-like sample for the first circle: (**a_1**) before immersed in, (**a_2**) being immersed in, (**a_3**) just taken out from liquid nitrogen, (**a_4**) after recovering to room temperature. **b_1-b_4** Optic photos of the Mobius strip-like sample for the tenth circle: (**b_1**) before immersed in, (**b_2**) being immersed in, (**b_3**) just taken out from liquid nitrogen, (**b_4**) after recovering to room temperature.

In order to investigate the low-temperature stability of PS-BPLCs, BPLCs film in Stage IV (with the size of 2 mm × 20 mm) is rolled up to form a Mobius strip (a_1). (a_1-a_4) present the photographs of the Mobius strip for the test of the first circle. The Mobius strip is put in liquid nitrogen for 1 min and then is taken out to recover to room temperature. (b_1-b_4) present the photos of the Mobius strip after being immersed in liquid nitrogen and taken out for 10 circles, the shape and color of the Mobius strip keep unchanged. Especially, iridescent color can be seen on the strip (green speckles on the strip) before and after 10 circles of immersing in or taking out from liquid nitrogen.

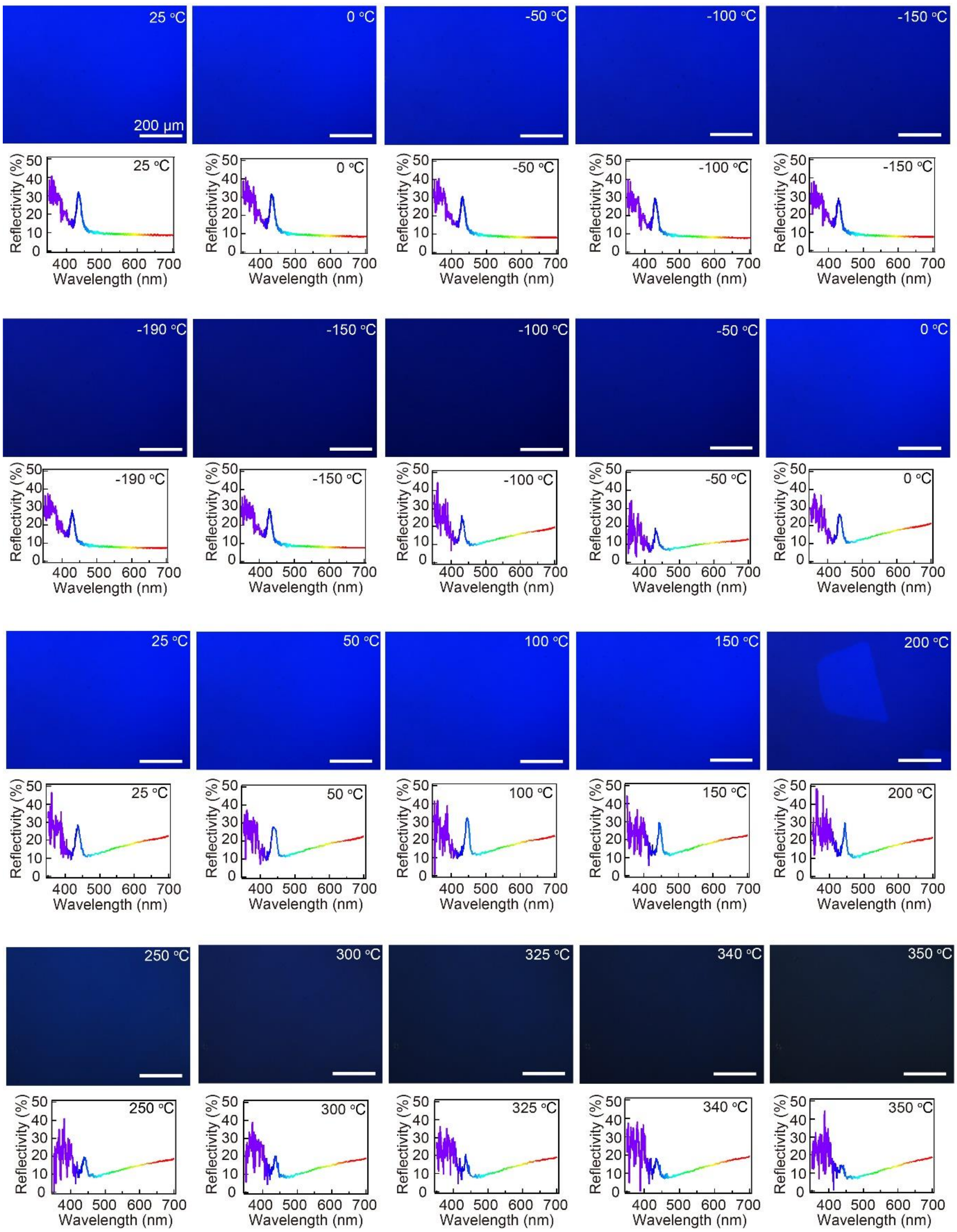

Supplementary Fig. 19 Thermal stability of monodomain polymer-stabilized BPII. POM images and *in situ* reflection spectra are captured at a certain temperature when the cell was cooled from 25 to -190 ℃ and heated up from -190 to 340 ℃.

To the best of our knowledge, the broadest temperature window reported by Choi et al. [6] based on a polymer-stabilized BPII is 50 ℃. Both polymer-stabilized BPIII/BPII/BPI core-shell configurations in Stage IV have a temperature window of over -190 to 340 ℃ (Supplementary

Fig. 17). To gain additional insights into the thermal stability of BPII, herein, detailed information is obtained by focusing on monodomain BPII at the same conditions.

The as-prepared monodomain polymer-stabilized $BPII_{\{100\}}$ is cooled at 5 ℃/min from 25 to -190 ℃ and then heated at 5 ℃/min from -190 to 340 ℃. The corresponding change of textures and reflection spectra are shown in Supplementary Fig. **19**. During the temperature reduction process, the maximum reflectivity slightly decreases when the temperature is close to -190 ℃, suggesting a possible glass transition[7]. When BPII is heated from -190 to 0 ℃， maximum reflectivity shows a tendency of decreasing first and increasing afterward. The maximum reflectivity of BPII gradually decreases when the temperature is further heated to 350 ℃.

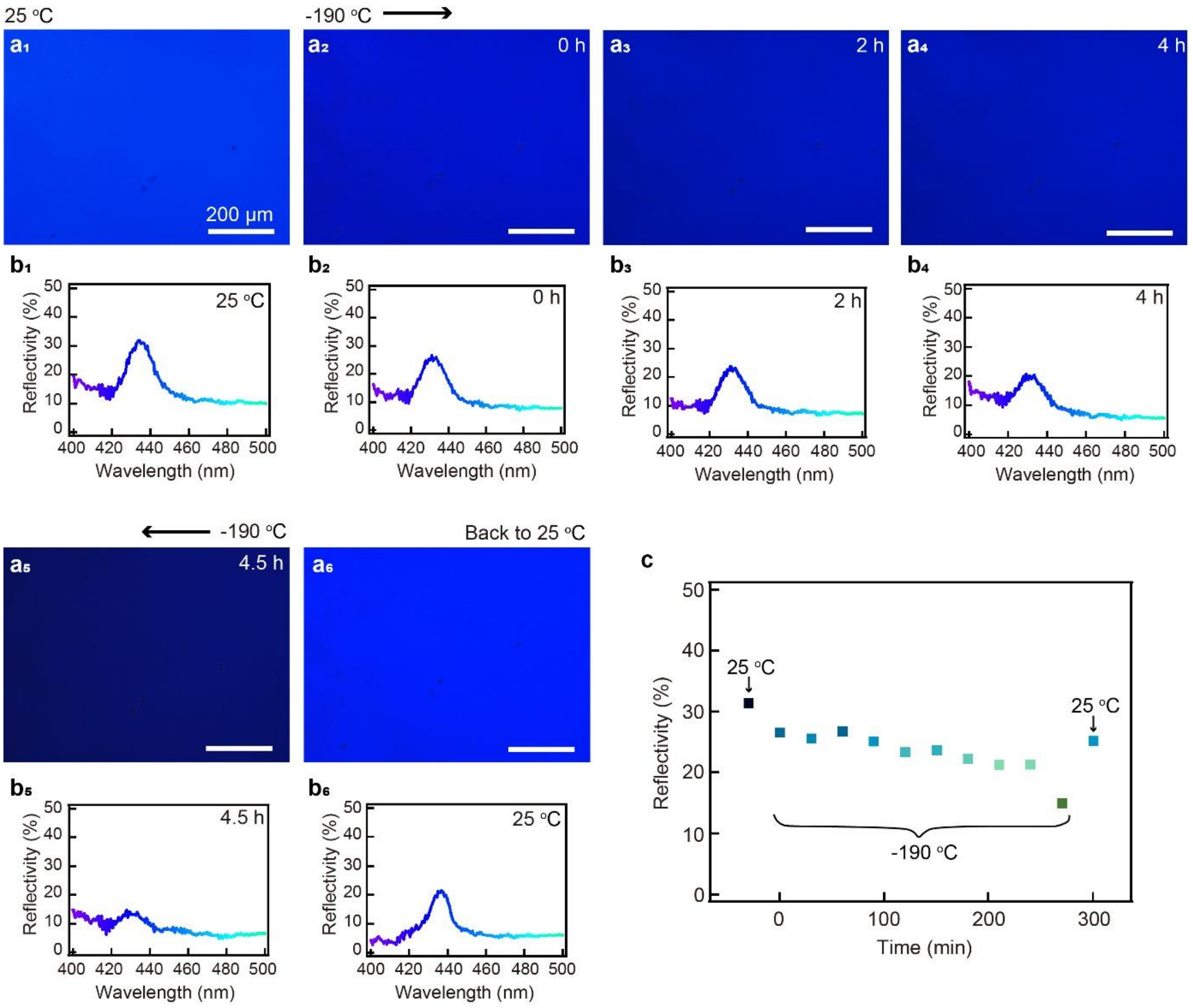

Supplementary Fig. 20 Thermal stability of monodomain polymer-stabilized BPII kept at -190 °C as a function of time. a$_1$, b$_1$ POM image (**a$_1$**) and the corresponding reflection spectrum (**b$_1$**) of the sample at 25 °C. **a$_2$-a$_5$** POM images of samples kept at -190 °C for 0-4.5 h. **b$_2$-b$_5$** Corresponding reflection spectra of (**a$_2$-a$_5$**). **a$_6$, b$_6$** POM image (**a$_6$**) and corresponding reflection spectrum (**b$_6$**) of the sample when temperature recovers to 25 °C. **c** Variation in reflectivity of the sample with a function of time.

The low-temperature stabilities of polymer-stabilized BPII are investigated. Monodomain BPII is firstly cooled from 25 to -190 °C at 5 °C/min and then is kept at -190 °C with a function of time. The textures and reflectivity of BPII can be well maintained within 4 h with only a slight decrease of reflectivity. After 4 h, the reflectivity falls to 14.644%, supposing to be a possible glass transition at such low temperatures. Then, optical properties are recovered after the sample is heated to 25 °C at 10 °C/min (c), except for a bit reduction of reflectively compared with the original sample (a$_1$).

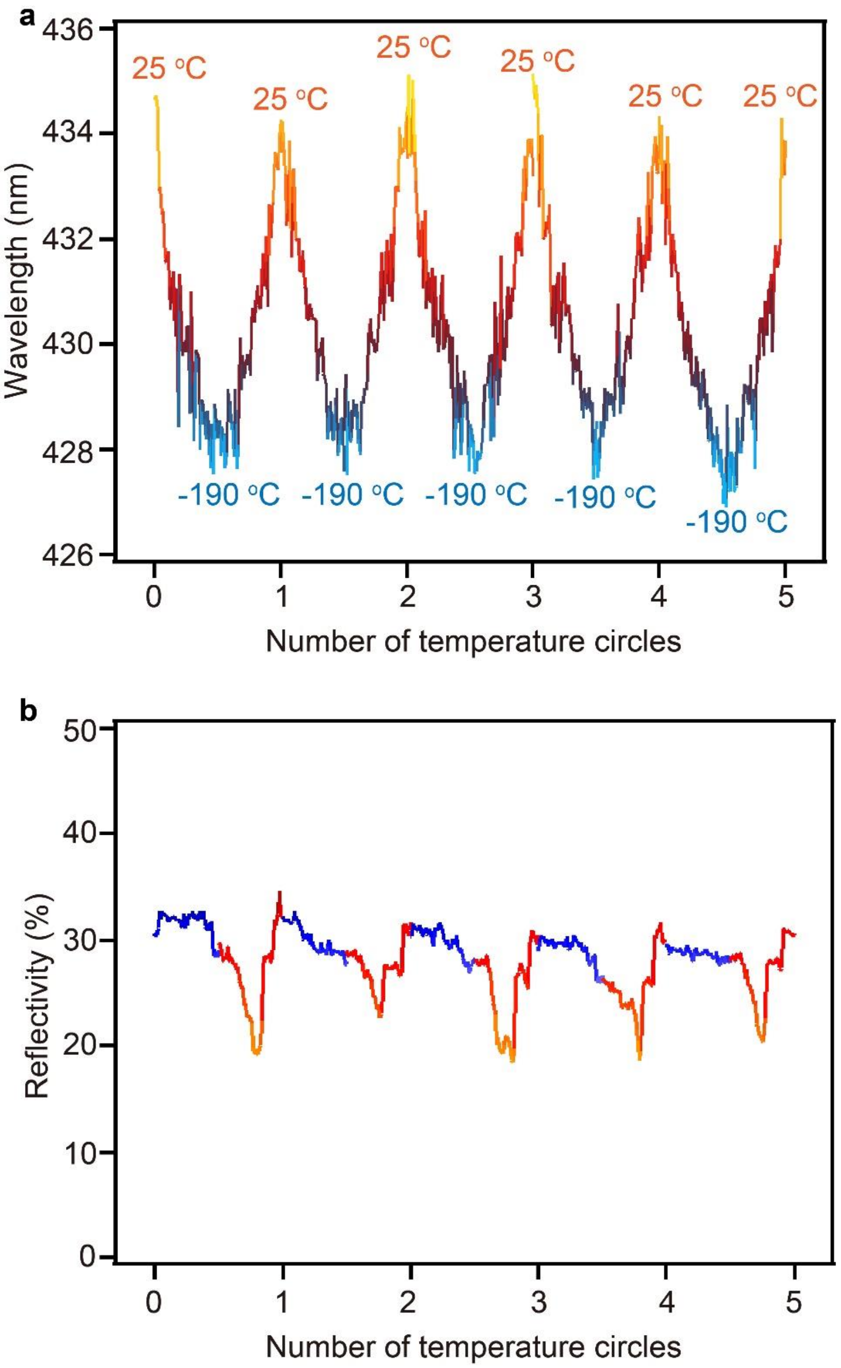

Supplementary Fig. 21 Optical stability of polymer-stabilized BPII during five temperature change circles from 25 to -190 ℃ and then recovers to 25 ℃. a Blue and red-shift of the center of reflection peak. **b** Reflectance changes of reflection peak.

The thermal stability of polymer-stabilized BPII in low temperatures is investigated. The optical properties of monodomain $BPII_{\{100\}}$ are investigated during 5 cycles within 25 to -190 ℃. With decreasing of temperature, the center of the stopband (λ_c) blue-shift from 435 to 427 nm. Then, the λ_c gradually red-shift to 435 nm with the rise of temperature. The reflectivity of polymer-stabilized $BPII_{\{100\}}$ is slightly reduced from approx. 33% to approx. 20% when the

temperature is lowered to -190 °C. Then, BPII$_{\{100\}}$ is heated from -190 to 25 °C and the maximum reflectivity shows a tendency of reducing first and increasing afterward. The maximum reflectivity recovers to the original state after the temperature recovers to 25 °C.

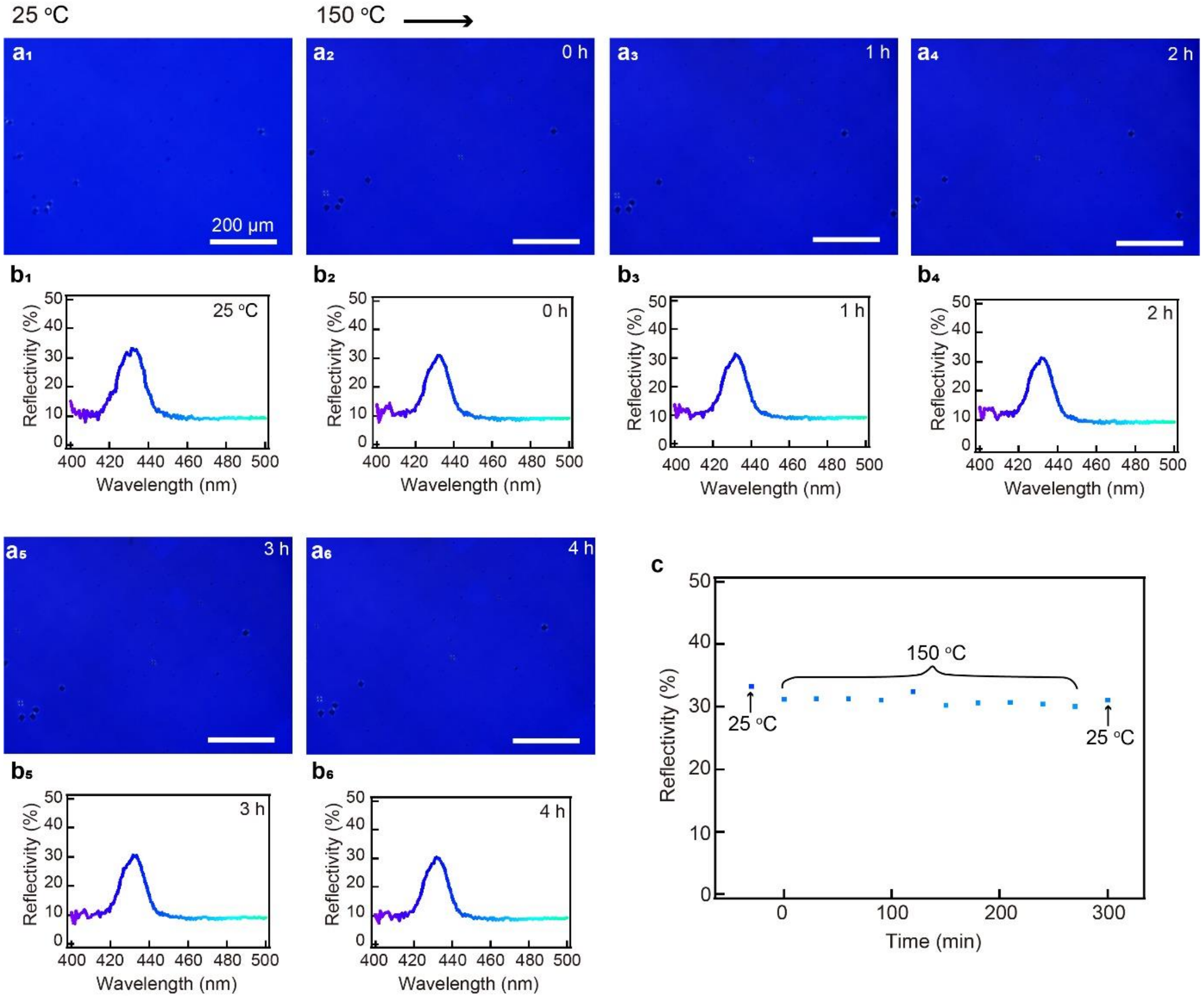

Supplementary Fig. 22 High-temperature stability of monodomain polymer-stabilized BPII kept at 150 °C as a function of time. $\mathbf{a_1}$, $\mathbf{b_1}$ POM image ($\mathbf{a_1}$) and the corresponding reflection spectrum ($\mathbf{b_1}$) of original monodomain polymer-stabilized BPII$_{\{100\}}$ captured at 25 °C. $\mathbf{a_2}$-$\mathbf{a_6}$, $\mathbf{b_2}$-$\mathbf{b_6}$ POM images ($\mathbf{a_2}$-$\mathbf{a_6}$) and the corresponding reflection spectra ($\mathbf{b_2}$-$\mathbf{b_6}$) of samples kept at 150 °C for 0-4.5 h. (c) Variation in reflectivity of the sample with a function of time.

The high-temperature stability of polymer-stabilized BPII is investigated. Monodomain BPII$_{\{100\}}$ is firstly cooled from 25 to -190 °C at 5 °C/min and then is heated to 150 °C. The temperature is kept at 150 °C with a function of time. There is no obvious degradation of texture and reflectivity. After 4.5 h, the sample is cooled to 25 °C at 5 °C/min.

Supplementary Note 4 Real-space TEM observation and crystal analysis of PS-BPLCs

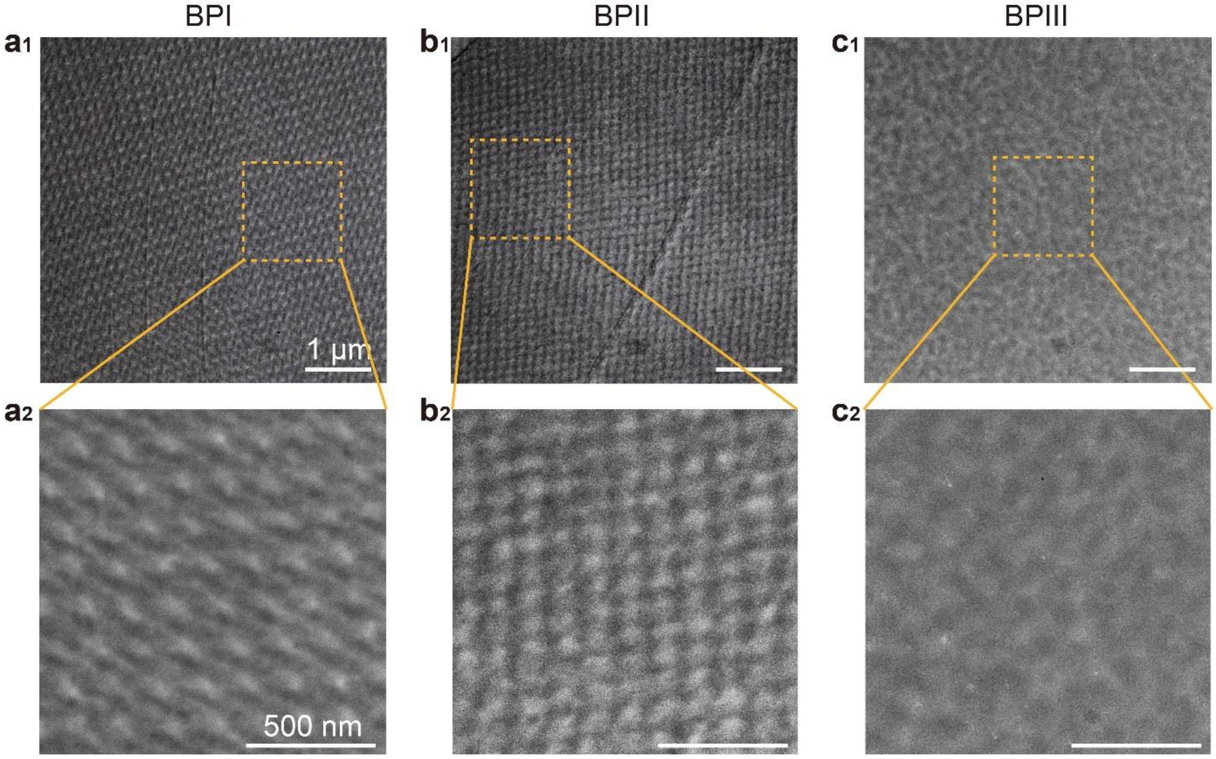

Supplementary Fig. 23 TEM images of (**a_1, a_2**) $BPI_{\{100\}}$, (**b_1, b_2**) $BPII_{\{100\}}$ and (**c_1, c_2**) BPIII. (**a_2-c_2**) Magnified TEM images of the squares highlighted in (**a_1-c_1**).

TEM observations are performed on ultrathin sections of PS-BPLCs with a thickness of 50 nm. The cuts are made parallel to $\{110\}_{BPI}$ and $\{100\}_{BPII}$ crystal plane of BPII. The arrangement of DTCs is observed in (a_1, a_2) $BPI_{\{100\}}$, (b_1, b_2) $BPII_{\{100\}}$ and (c_1, c_2) BPIII. Both $BPI_{\{100\}}$ and $BPII_{\{100\}}$ are highly ordered while BPIII has a random structure for its amorphous structures.

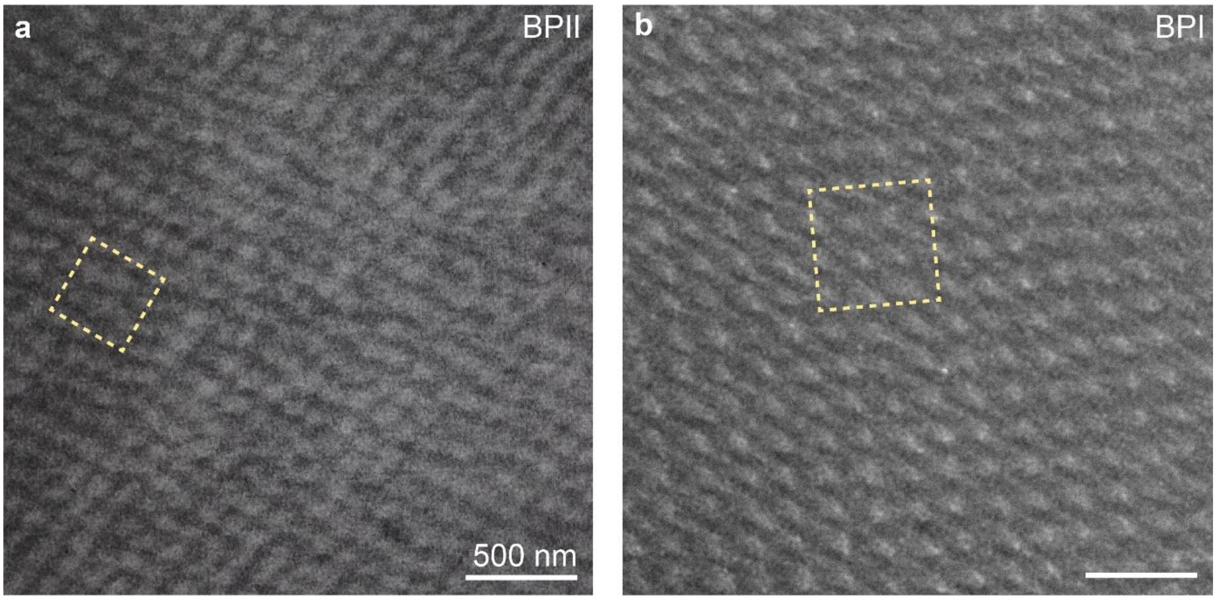

Supplementary Fig. 24 TEM images of BPII$_{\{100\}}$ (**a**) and BPI$_{\{100\}}$ (**b**). The yellow squares highlight the area with 3×3 unit cells of BPII$_{\{100\}}$ and BPI$_{\{100\}}$.

A closer inspection of BPII$_{\{100\}}$ (a) and BPI$_{\{100\}}$ (b) structures revealing that the lattice constant of BPI is larger than BPII.

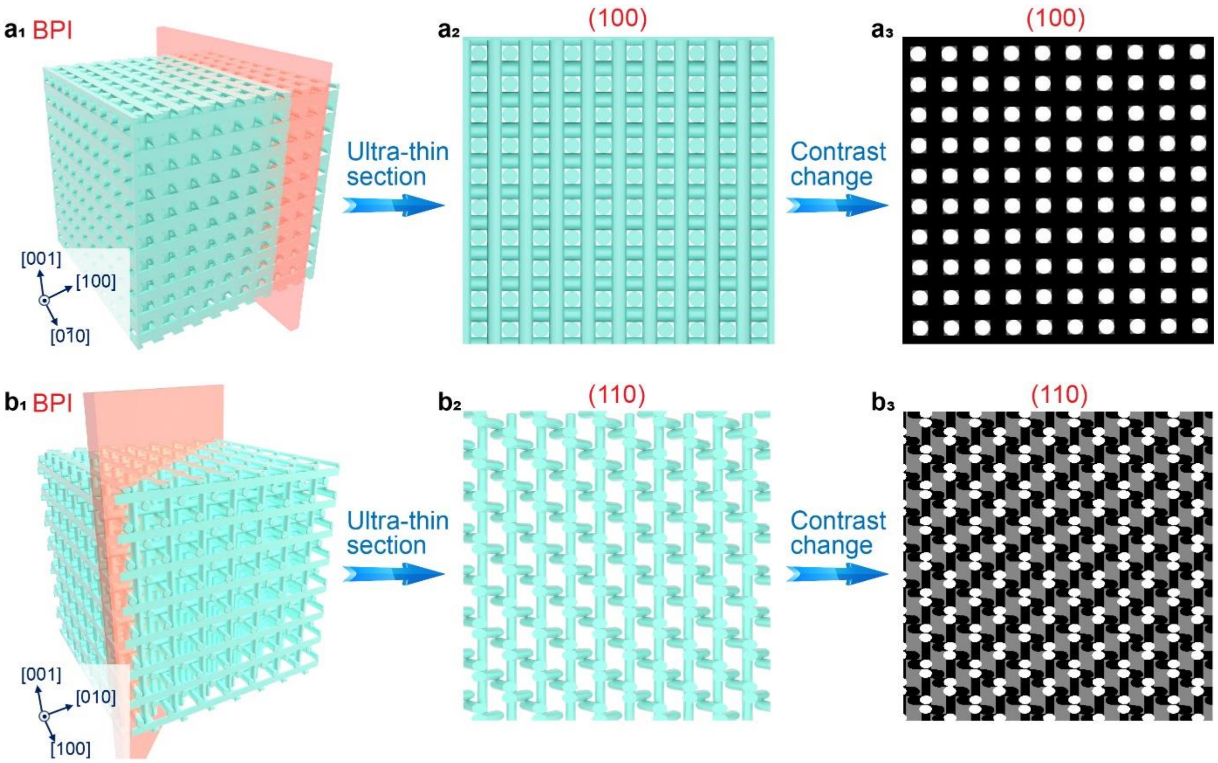

Supplementary Fig. 25 Theoretically predicted cross-sectional structure of (a) BPII or (b) BPI for BPLCs. a_1, b_1 Theoretically predicted arrangement of the DTCs in the 3D model. **a_2, b_2** Ultra-thin section of the sample with a thickness of 50 nm corresponding to the region shaded in red (**a_1, b_1**), viewed along the [100] ([110]) direction for BPII (BPI). **a_3, b_3** Contrast changed the image of (**a_2, b_2**) depending on the orientation of DTCs.

The predicted patterns with contrast change (a_3, b_3) are obtained from the top view of the slice (a_2, b_2) cut from the red slice in the 3D BPII or BPI model (a_1, b_1). Considering that the LC director is oriented parallel to the cylinder axis within a DTC. DTCs should appear alternated bright or dark at the sites where they are vertical or parallel to the slice plane. The contrast change of the intermediate brightness is used in the region without DTCs[8]. Theoretically predicted TEM slices (a_3, b_3) are used as representations of the DTCs arrangement of experiment results.

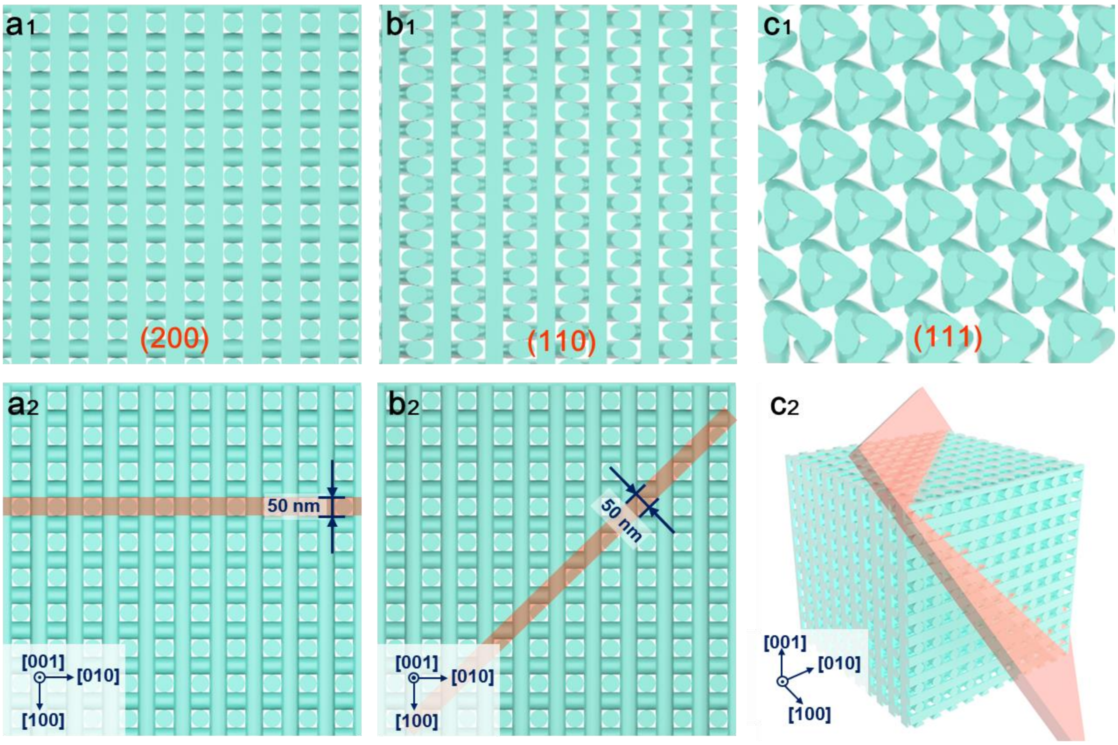

Supplementary Fig. 26 Schematic representation of the DTCs arrangement of BPII with distinct orientation. $\mathbf{a_1}$-$\mathbf{c_1}$ Ultra-thin section models are relative to those shaded in red of ($\mathbf{a_2}$-$\mathbf{c_2}$). $\mathbf{a_2}$-$\mathbf{c_2}$ Models of DTCs arrangement of BPII with distinct crystal orientations.

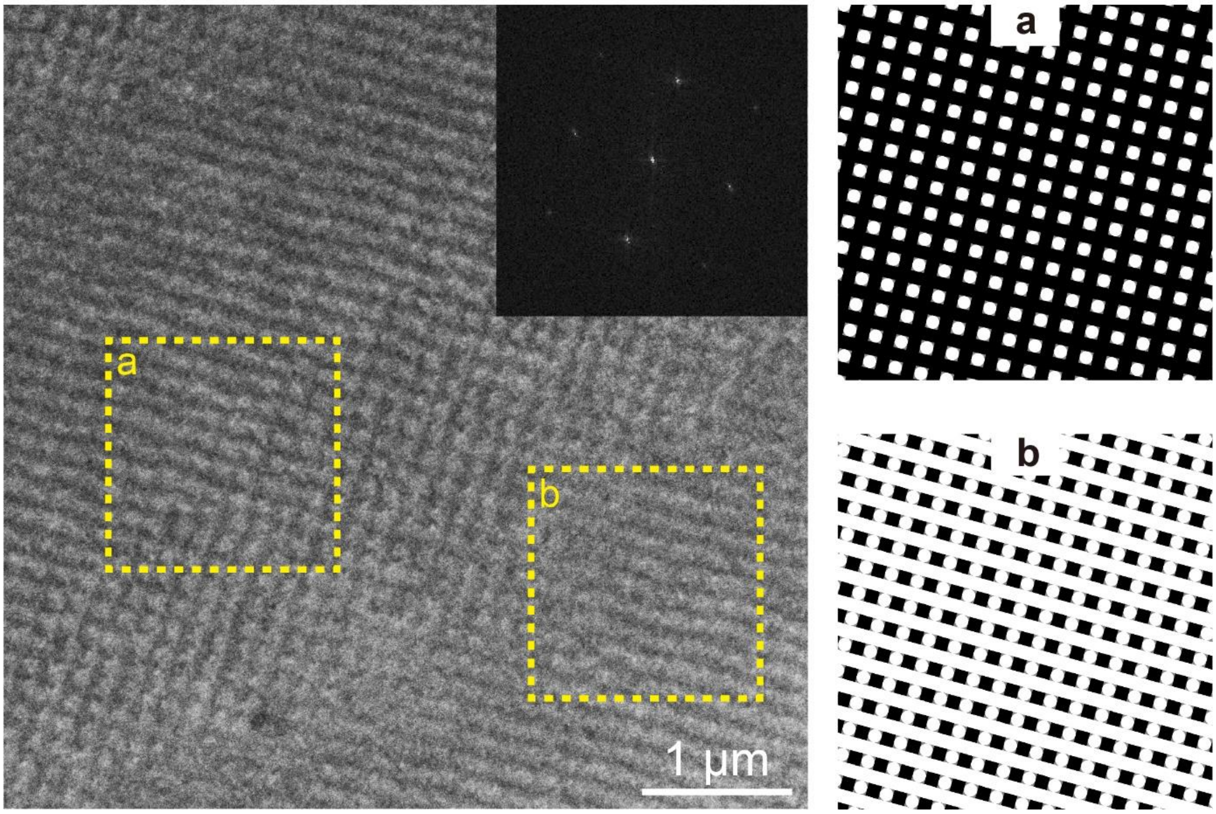

Supplementary Fig. 27 Two types of structures observed in a TEM image corresponding to BPII$_{\{100\}}$ after polymer-stabilization. The inserted image is an FFT pattern transferred from the TEM image. **a** The structure obtained when the upper part of the unit cell is cut and (**b**) is obtained when the bottom part of the unit cell is cut.

The lattice constant of BPII of 171.82 nm is measured from the FFT pattern in the inserted image. Two types of TEM structures belonging to BPII$_{\{100\}}$ can be observed both in experimental and predicted results. Owing to the thickness of slices (approx. 50 μm) obtained from the ultra-thin section process are much smaller than the lattice constant of BPII. the structures shown in (a) are observed when the upper part of the unit cell is cut during the ultra-thin section process. And the structures shown in (b) are observed once the bottom part of the unit cell is cut.

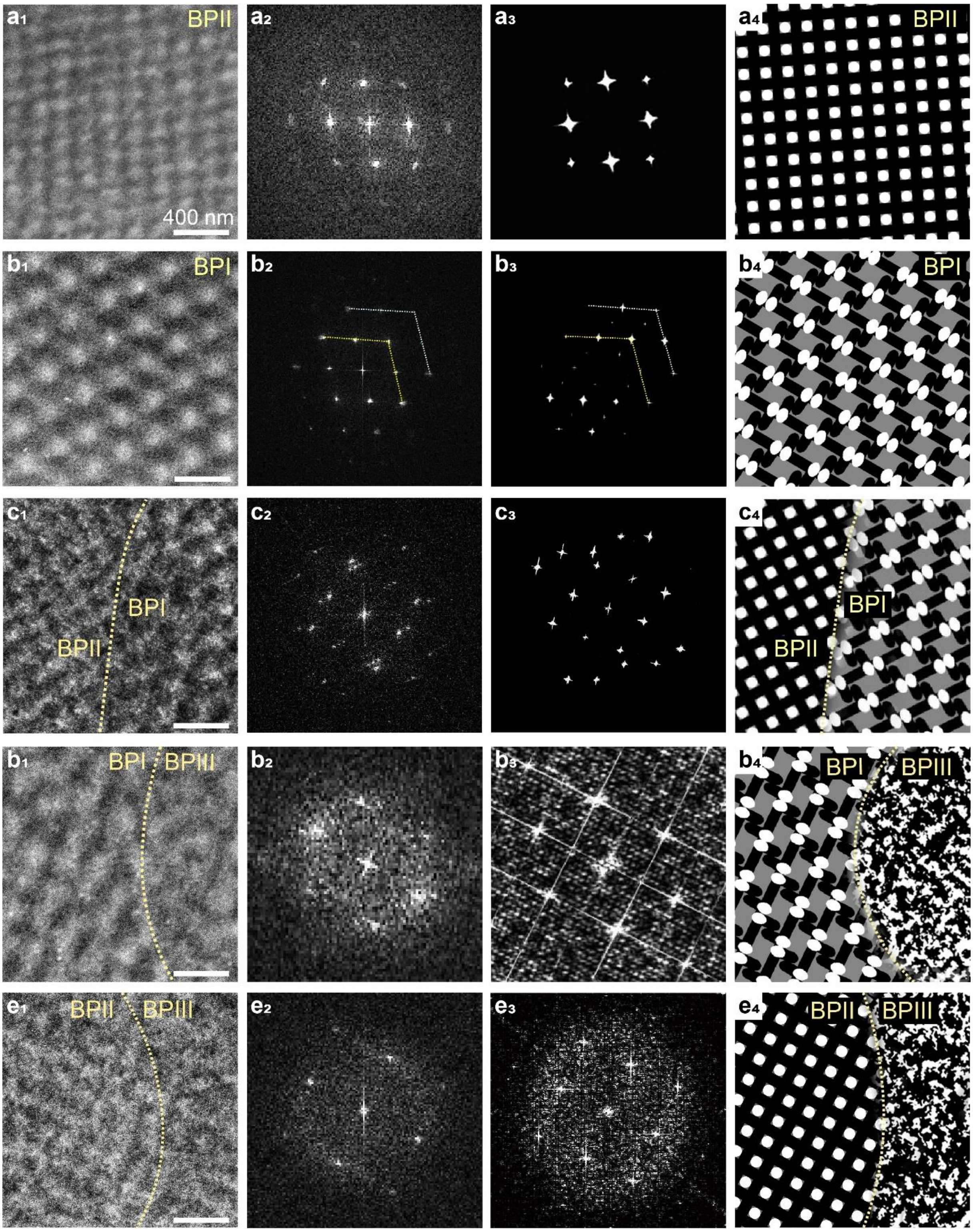

Supplementary Fig. 28 TEM images (**a_1-e_1**), corresponding fast Fourier transform (FFT) patterns (**a_2-e_2**), theoretically predicted FFT patterns (**a_3-e_3**) of BPLCs, and theoretical models for the arrangement of DTCs (**a_4-e_4**). (**a**) BPII$_{\{100\}}$, (**b**) BPI$_{\{110\}}$, (**c**) interface between BPII$_{\{100\}}$ and BPI$_{\{110\}}$, (**d**) interface between BPI$_{\{110\}}$ and BPIII, and (**e**) interface between BPII$_{\{100\}}$ and BPIII. The dotted yellow lines highlight the interfaces between BPI, BPII, and BPIII.

Detailed TEM images and corresponding theoretically predicted DTCs arrangement of phase interfaces are presented here. (a_1-a_4) are monophasic BPII$_{\{100\}}$. A highly ordered

microscopic periodic structure with a lattice constant of 171.82 nm in TEM image is observed (a_1) which clearly indicates a four-fold symmetry in the observed contrasts, which agrees with the theoretically predicted symmetry along [100] direction (a_4). (a_4) is theoretically predicted arrangement of DTCs and the structures are obtained by cutting from the 3D model and change the contrast based on the method shown in (Supplementary Fig. 27). (a_3) is the FFT pattern transferred from (a_4), in which the speckles have the same position as (a_2), indicating the real-space structures of (a_1) and (a_4) have the same arrangement of DTCs and size. Thus, the predicted DTCs arrangement in (a_4) can represent the structures in (a_1). Similarly, (b_1-b_4) are monophasic BPI. In the BPI lattice, the lattice constant a is measured as 252.57 nm. FFT analysis (b_2) shows a sharp diffraction pattern of periodic $BPI_{\{110\}}$ which is similar to the theoretically predicted result (b_3), indicating predicted DTCs arrangement (b_4) can represent the results in TEM images (b_1). (c_1-c_4) show the interface between $BPII_{\{100\}}$ and $BPI_{\{110\}}$. A clear interface can be observed from the TEM image (c_1). Both the FFT pattern (c_2) transferred from the TEM image and the similar theoretically predicted result (c_3) yield a combined sharp diffraction speckles of $BPI_{\{110\}}$ and four-fold symmetry of $BPII_{\{100\}}$. All speckles of $BPI_{\{110\}}$ and $BPII_{\{100\}}$ indicate the highly ordered structures near the interface and phase transition occurs in submicron scales without transitional region. (d_1-d_4) depict the sample with the interface between BPIII and $BPI_{\{110\}}$ at Stage III. TEM image of a clear interface between BPIII and $BPI_{\{110\}}$ (d_1) is observed. FFT analysis (d_2) shows combined diffraction of $BPI_{\{110\}}$ and BPIII: the diffraction pattern from the periodic $BPI_{\{110\}}$ and a scatting background from the amorphous structure of BPIII. TEM slice derived from the theoretical model is obtained in (d_4). FFT pattern in (d_3) is transferred from a theoretically predicted arrangement of DTCs near the boundary region (d_4). Comparing the FFT pattern transferred from the experimental and predicted result, the diffraction patterns are the same. Therefore, the DTCs arrangement in TEM result (d_1) can be represented by predicted results (d_4), indicating BPIII-to-BPI transformation is diffusionless without a transitional region near the interface. (e_1-e_4) demonstrate the interface

between BPIII and $BPII_{\{100\}}$. A clear interface can be observed from the TEM image (e_1). FFT pattern (e_2) shows the combination of the periodic diffraction pattern of $BPII_{\{100\}}$ and the adjacent amorphous structure of BPIII. Theoretically predicted FFT pattern (e_3) is similar to that obtained from experimental results, indicating the arrangement of DTCs in TEM (e_1) is the same as a predicted result (e_4).

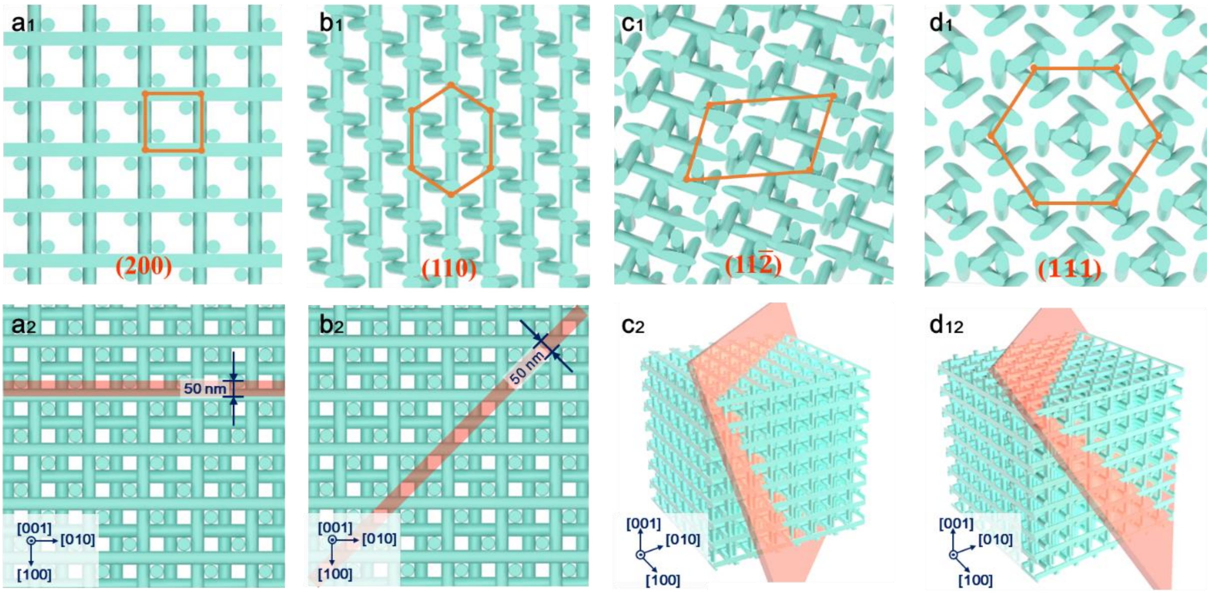

Supplementary Fig. 29 Schematic representation of DTCs arrangement of BPI with distinct orientation. **a_1-d_1** Section models correspond to those shaded in red (**a_2-d_2**). The geometric figures in orange represent the typical arrangement that may be observed in the TEM images. **a_1-d_1** DTCs distributed within a thin section of BPI with distinct crystal orientation.

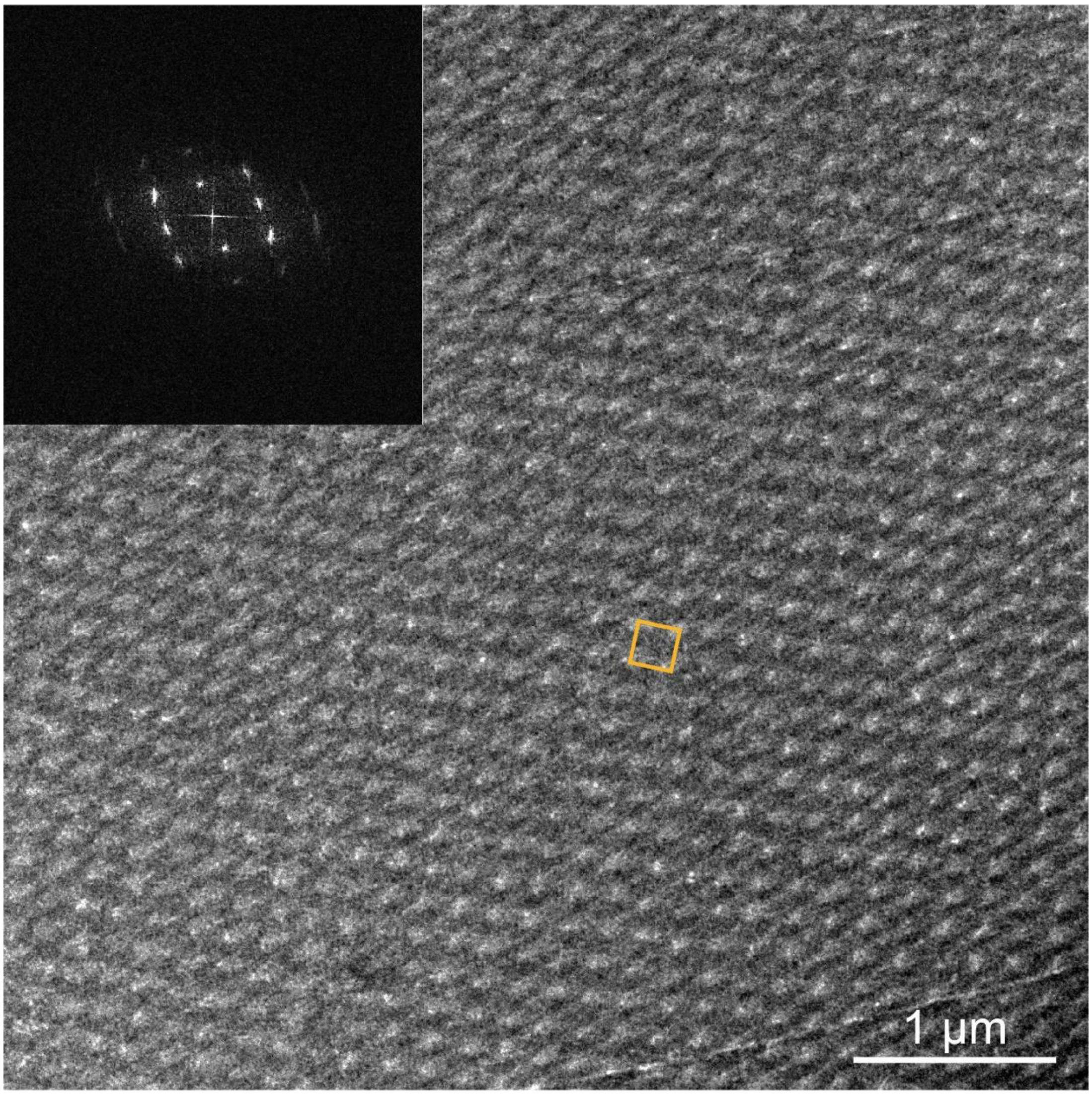

Supplementary Fig. 30 TEM image of BPI$_{\{100\}}$ after polymer-stabilization. The inserted image is an FFT pattern transferred from the TEM image. A rectangle highlights a unit cell of BPI which in accordance with the predicted arrangement of DTCs in Supplementary Fig. 29a$_1$

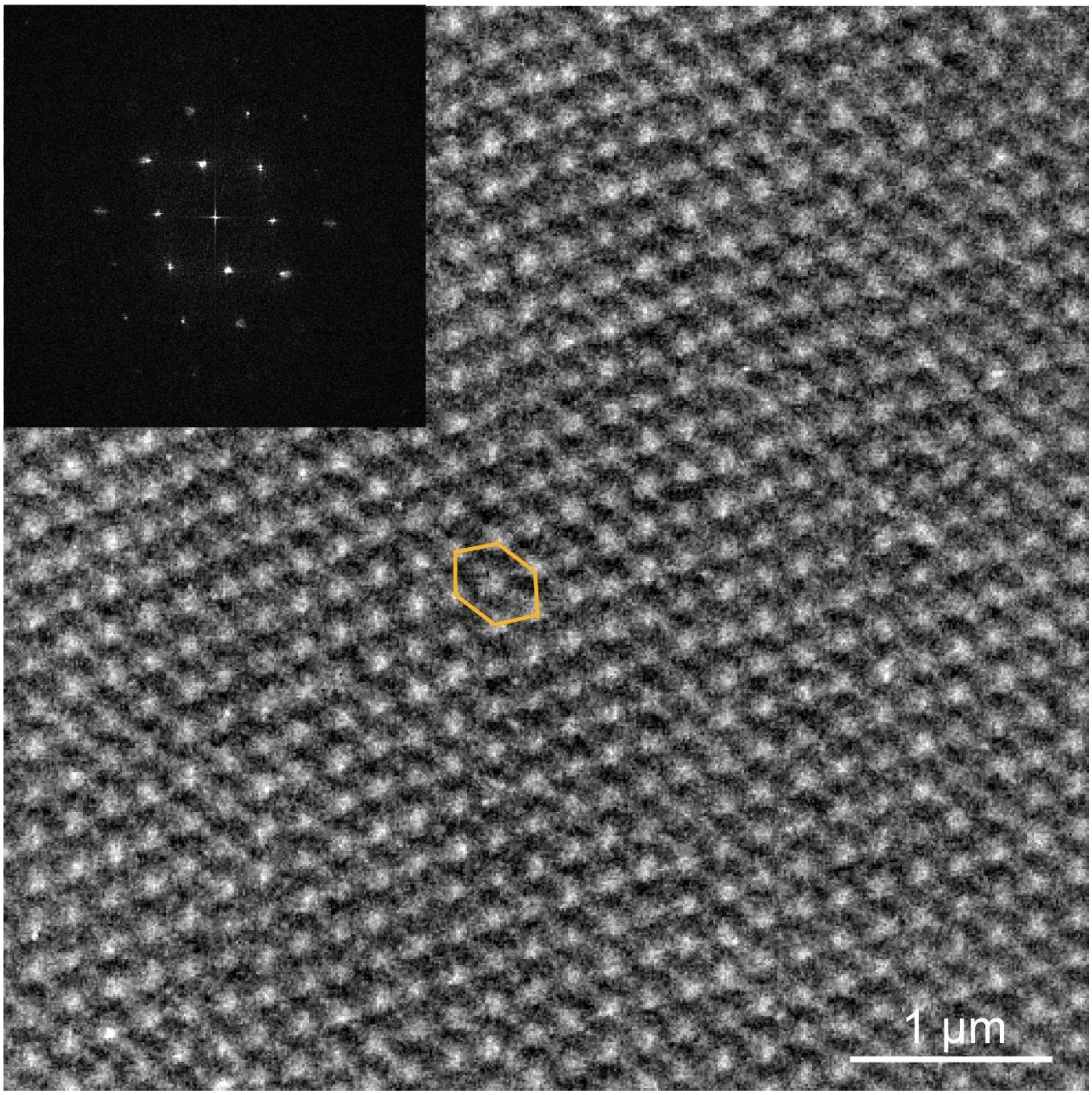

Supplementary Fig. 31 TEM image of $BPI_{\{110\}}$ after polymer-stabilization. The inserted image is an FFT pattern transferred from the TEM image.

The lattice constant of BPI (252.57 nm) is measured from the FFT pattern in the inserted image. The flat hexagons can be found in a periodic structure which is in accordance with the predicted arrangement of DTCs in Supplementary Fig. $29b_1$

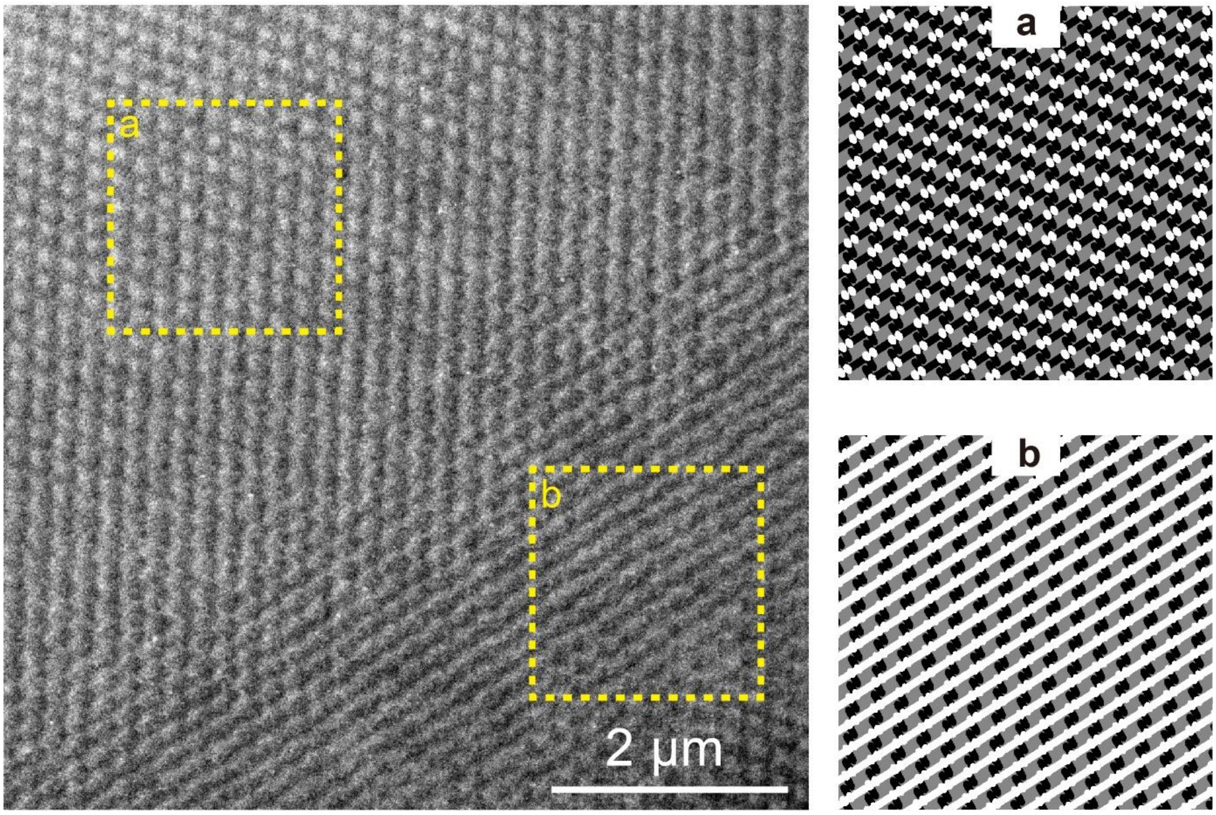

Supplementary Fig. 32 Two types TEM images of $BPI_{\{110\}}$ after polymer-stabilization. a The structure obtained when the upper part of the unit cell is cut and (**b**) the bottom part of the unit cell is cut.

Two types of TEM structures of $BPI_{\{110\}}$ are observed both in experimental results and predicted results. Owing to the thickness of slices (approx. 50 μm) obtained from the ultra-thin section process is much smaller than the lattice constant (252.57 nm). The structures shown in (a) are observed when the upper part of the unit cell is cut. And the structures shown in (b) are observed once the bottom part of the unit cell is cut.

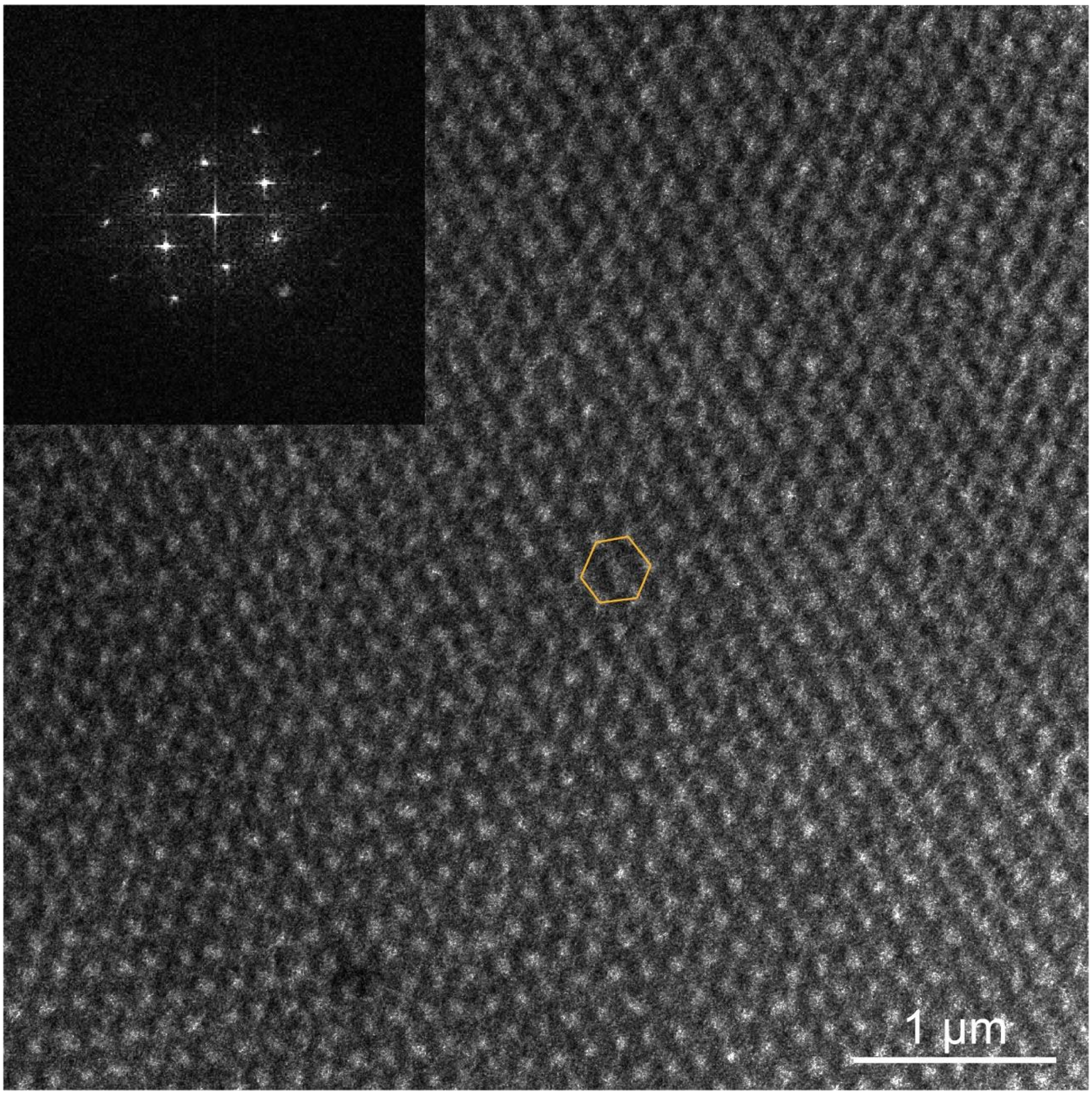

Supplementary Fig. 33 TEM image of $BPI_{\{111\}}$ after polymer-stabilization. The inserted image is an FFT pattern transferred from the TEM image.

The regular hexagons can be found in a periodic structure which is in accordance with the predicted arrangement of DTCs in Supplementary Fig. $29d_1$

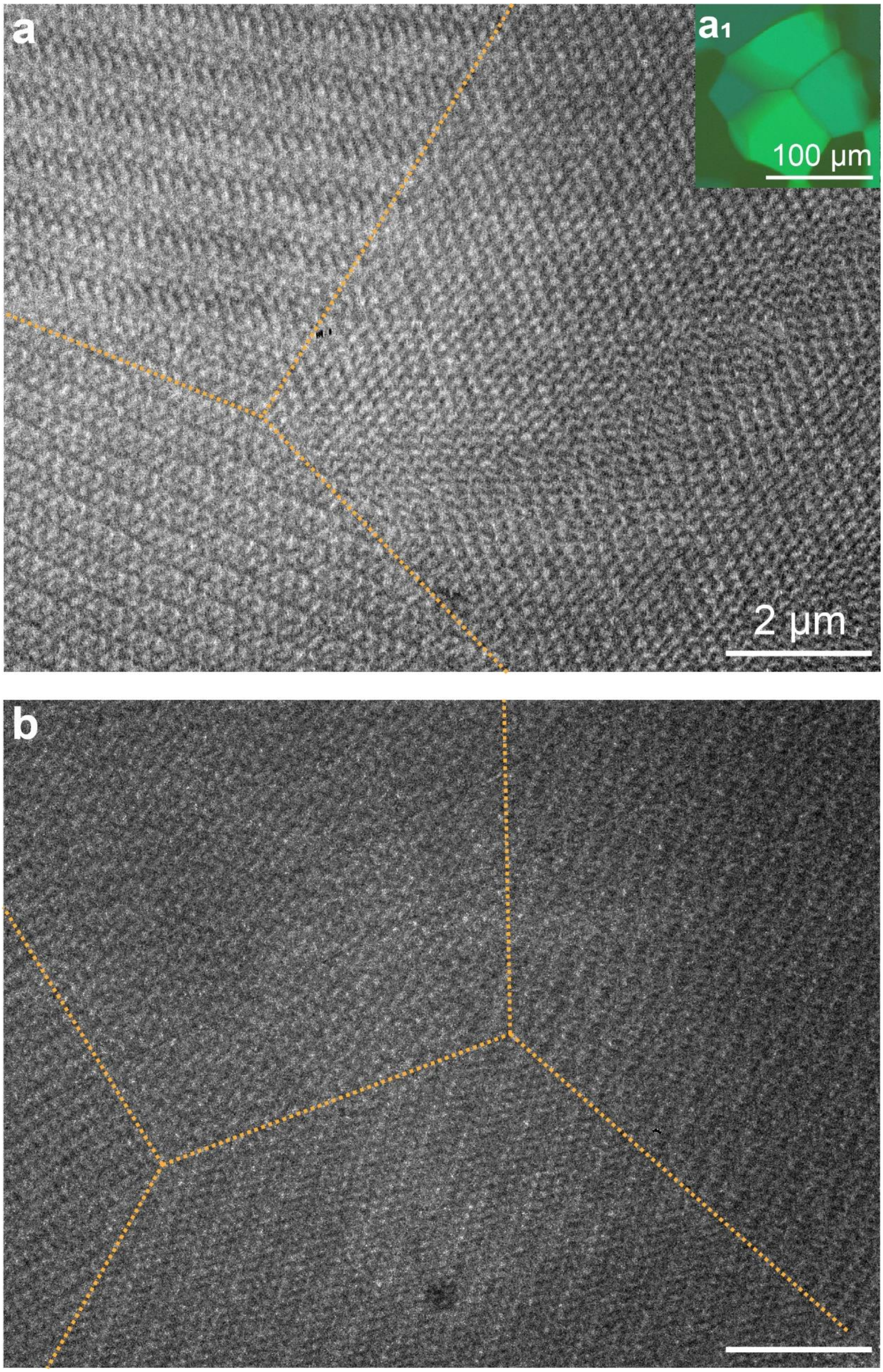

Supplementary Fig. 34 TEM images of the interfaces among domains with distinct orientations of polydomain BPI. a Interfaces among three domains in polydomain BPI. **b** Interfaces among four domains in polydomain BPI.

Excepting for the interface among BPI, BPII, and BPIII, TEM images of the interfaces corresponding to three and four domains of polydomain BPI are shown. The inserted image of (a_1) shows the POM images of grains of polydomain BPI.

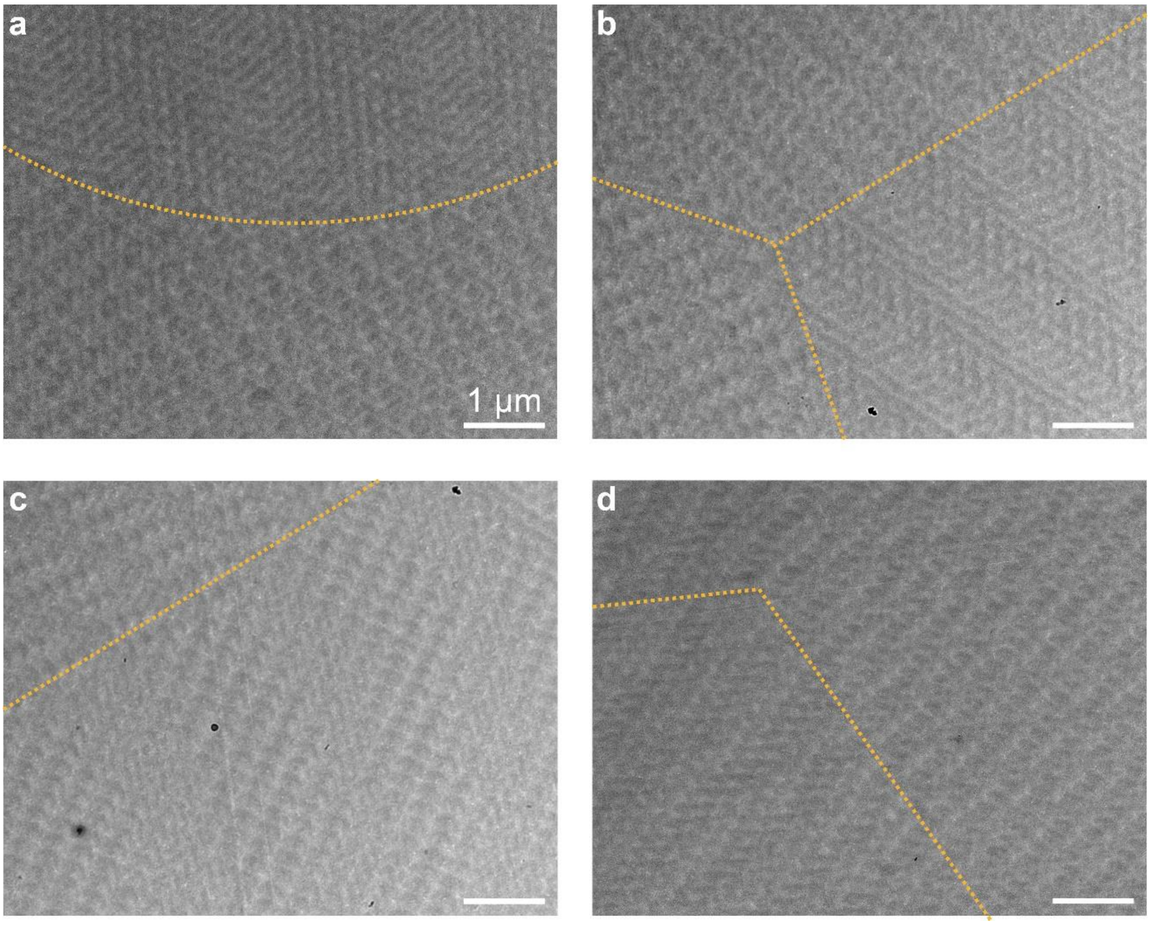

Supplementary Fig. 35 a-d Amplified TEM images of the interfaces among domains with distinct orientations of polydomain BPI. The orange dotted line highlights the interfaces.

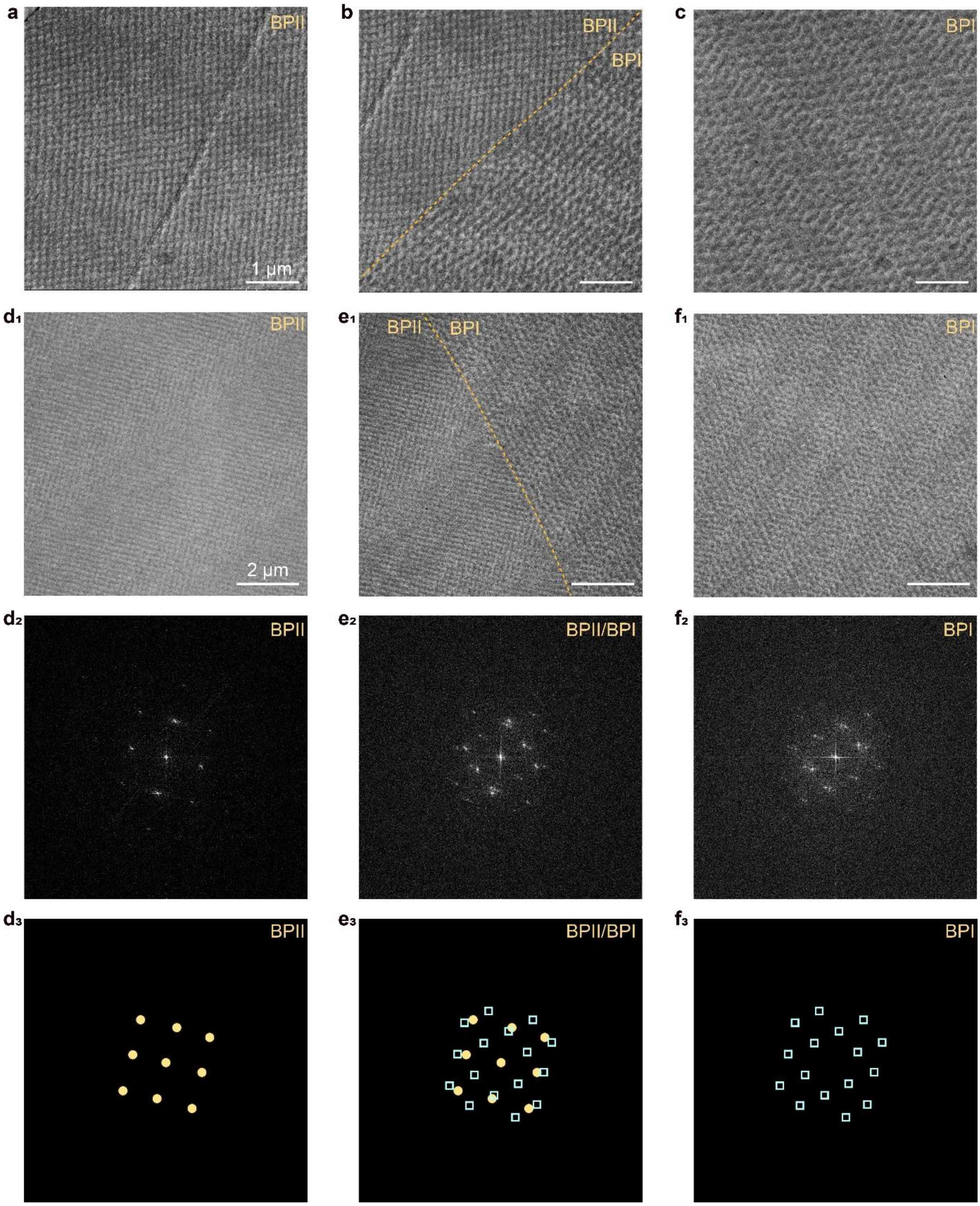

Supplementary Fig. 36 TEM images and the corresponding FFT patterns of the interface between $\mathbf{BPII_{\{100\}}}$ and $\mathbf{BPI_{\{110\}}}$. (**a, d_1, d_2, d_3**) $BPII_{\{100\}}$, (**b, e_1, e_2, e_3**) the interface of $BPI_{\{110\}}$/$BPII_{\{100\}}$, and (**c, f_1, f_2, f_3**) $BPI_{\{110\}}$. **d_2, e_2, f_2** FFT patterns were transferred from the areas in (**d_1, e_1, f_1**), respectively. (**d_3, e_3, f_3**) Schematic diffraction patterns highlight the same position of the speckles of experiment results in (**d_2, e_2, f_2**). **d_3** Diffraction speckles of $BPII_{\{100\}}$ labeled by yellow solid circles; **e_3** simple addition of schematic diffraction patterns of monophasic $BPII_{\{100\}}$ from (d_3) and $BPI_{\{110\}}$ from (f_3). where the yellow solid circles represent diffraction speckles of $BPII_{\{100\}}$ and blue hollow squares represent diffraction speckles of $BPI_{\{110\}}$; **f_3** diffraction speckles of $BPI_{\{110\}}$ labeled by blue hollow squares;

Especially, FFT patterns of the interface between $BPI_{\{110\}}$ and $BPII_{\{100\}}$ (e_2, e_3) possess two sets of sharp diffraction speckles, which can be regarded as a simple addition of diffraction patterns of monophasic $BPII_{\{100\}}$ (d_2, d_3) and $BPI_{\{110\}}$ (f_2, f_3). It is confirmed by FFT analysis that the size and position of diffraction spots near the interface (e_2, e_3) are the same as those in the bulk for $BPII_{\{100\}}$ (d_2) highlighted by yellow solid circles (d_3) and $BPI_{\{110\}}$ (e_2) highlighted by blue hollow squares (e_3), proving there is just a diffusionless reconfiguration of DTCs (equally to no transitional region near the interface).

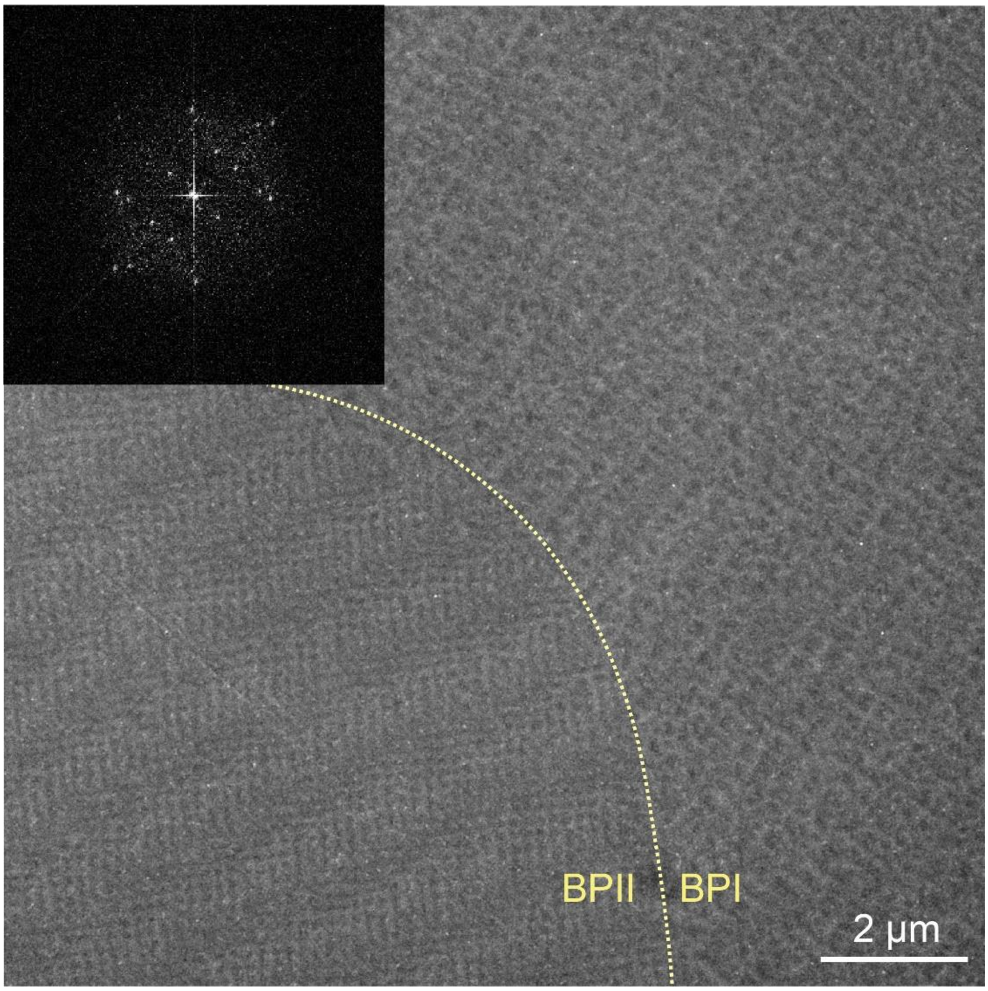

Supplementary Fig. 37 TEM image of the interface between BPII and BPI in real-space. The inserted image is the FFT pattern transferred from the TEM image. The interface is highlighted by a yellow dotted line.

Herein, the BPII has the [100] orientation out-of-plane while the BPI has the orientation other than [110] direction out-of-plane.

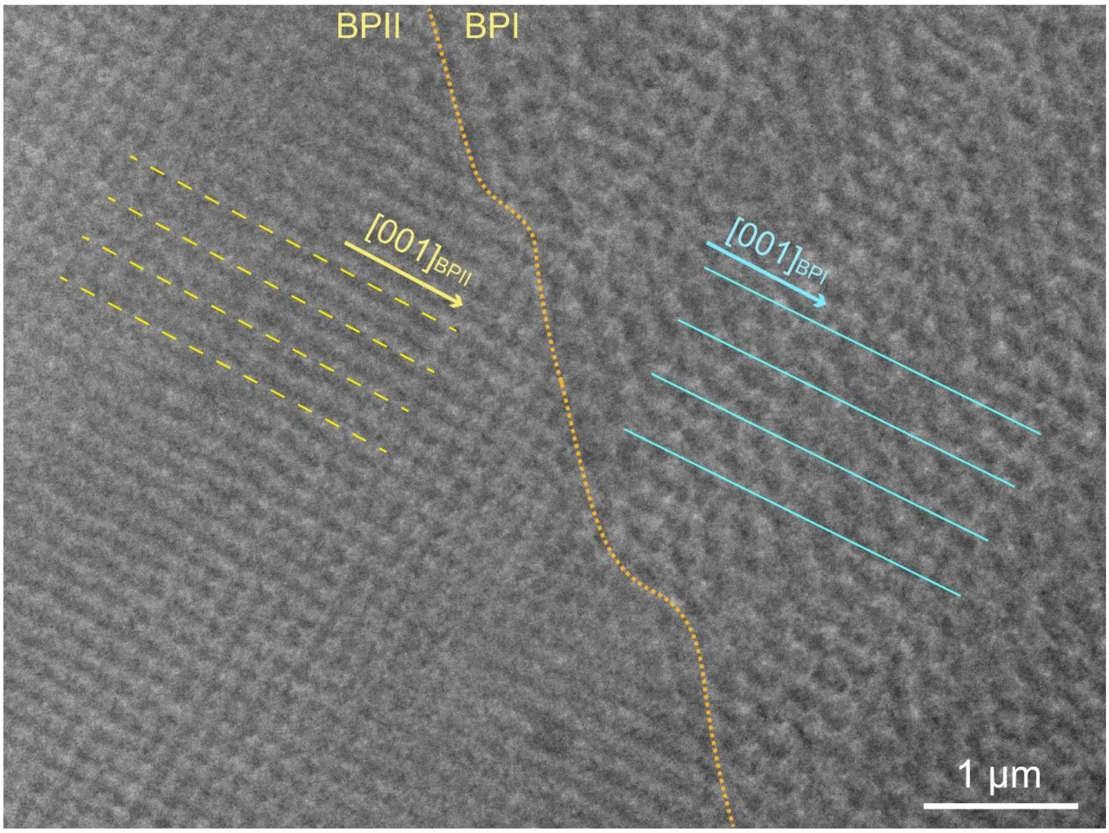

Supplementary Fig. 38 TEM image of the interface between BPII$_{\{100\}}$ and BPI$_{\{110\}}$. The orange dotted line represents the interface. The yellow dash line parallel to the [001] orientation of BPII$_{\{100\}}$ and blue solid lines represent the [001] orientation of BPI$_{\{110\}}$.

More specifically, the orientation of BPI is influenced by BPII and the crystal lattice of BPI and BPII is coherent on the interface.

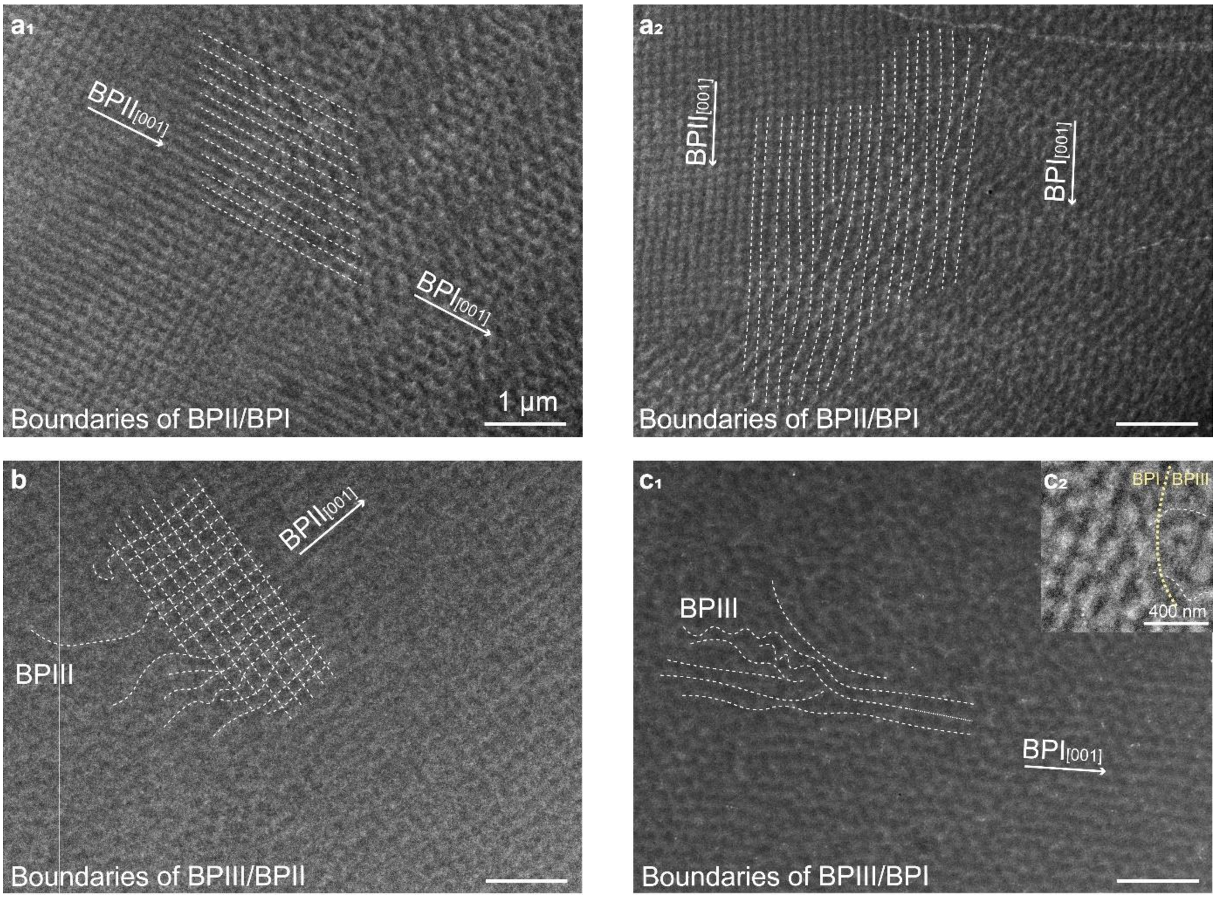

Supplementary Fig. 39 TEM observation of the interfaces among BPIII, BPII, and BPI. a_1-a_2 Coherent crystal lattices between the interfaces of $BPII_{\{100\}}/BPI_{\{110\}}$. DTCs arrangement of the well-matched parts is highlighted by dotted white lines. **b** The interface between BPIII and $BPII_{\{100\}}$, in which a DTC can simultaneously belongs to crystal lattices of BPIII and $BPII_{\{100\}}$. **c_1** The interface between BPIII and $BPII_{\{100\}}$, where curved lines highlight the DTCs that coexist in both $BPII_{\{100\}}$ and BPIII unit cells. **c_2** Magnified TEM image of the interface of $BPI_{\{110\}}$/BPIII, where the dotted lines highlight the DTCs observed across the interface between BPIII and $BPI_{\{110\}}$.

The position of DTCs and the coherent crystal lattices are investigated by TEM at the interfaces of BPII/BPI, BPIII/BPII, and BPIII/BPI. As for phase transformation between BPI and BPII, the interfaces are coherent (a_1, a_2), in which a DTC simultaneously belongs to BPII unit cells and BPI unit cells. These coherent lattices provide evidence for the thermoelastic martensitic transformation between BPII and BPI.

Thanks to the clear interfaces observed by TEM between BPIII and $BPII_{\{100\}}$ (b), the speckles with four-fold symmetric highlighted by the dotted grid belong to $BPII_{\{100\}}$ while the curved lines besides the grid highlight the DTCs simultaneously exist in both $BPII_{\{100\}}$ and BPIII, confirming no transitional area between $BPII_{\{100\}}$ and BPIII. Thus, during the phase

transformation between BPIII and BPII, the DTCs reconfiguration will directly form BPIII phase rather than a process of diffusion first and then reorganize.

In terms of the phase transformation between BPIII and BPI (c), a phenomenon was observed similarly to that of BPII and BPIII. Therefore, the phase transformation between BPIII and BPI is diffusionless.

In a word, by direct observation of the arrangement of DTCs by TEM, several pieces of evidence were obtained for the confirmation of the DLPT among BPIII, BPII, and BPI.

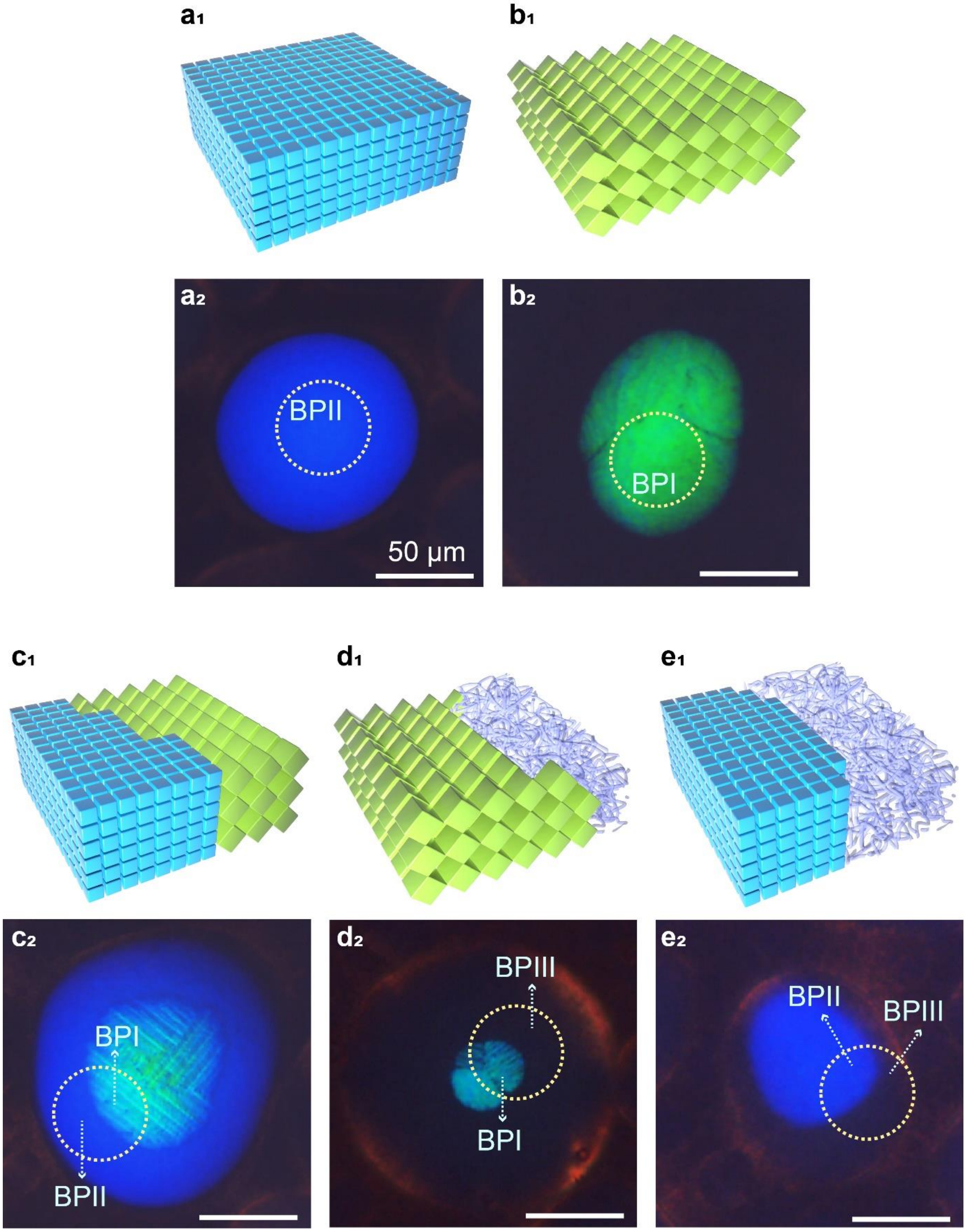

Supplementary Fig. 40 Interfaces of core-shell configuration at Stage III. a_1-e_1 Schematic illustration of the arrangement of unit cells in the monophasic BPII and BPI or the interfaces between BPIII, BPII, and BPI. **a_2-e_2** The textures taken on a crossed POM under reflection mode. The yellow dotted circle highlights the interface or monophasic at Stage III. **a_2** The polymer-stabilized monophasic BPII$_{\{100\}}$. **b_2** The polymer-stabilized monophasic BPI$_{\{110\}}$. **c_2** The polymer-stabilized interface between BPII$_{\{100\}}$ and BPI$_{\{110\}}$. **d_2** The polymer-stabilized interface between BPI$_{\{110\}}$ and BPIII. **e_2** The polymer-stabilized interface between BPII$_{\{100\}}$ and BPIII.

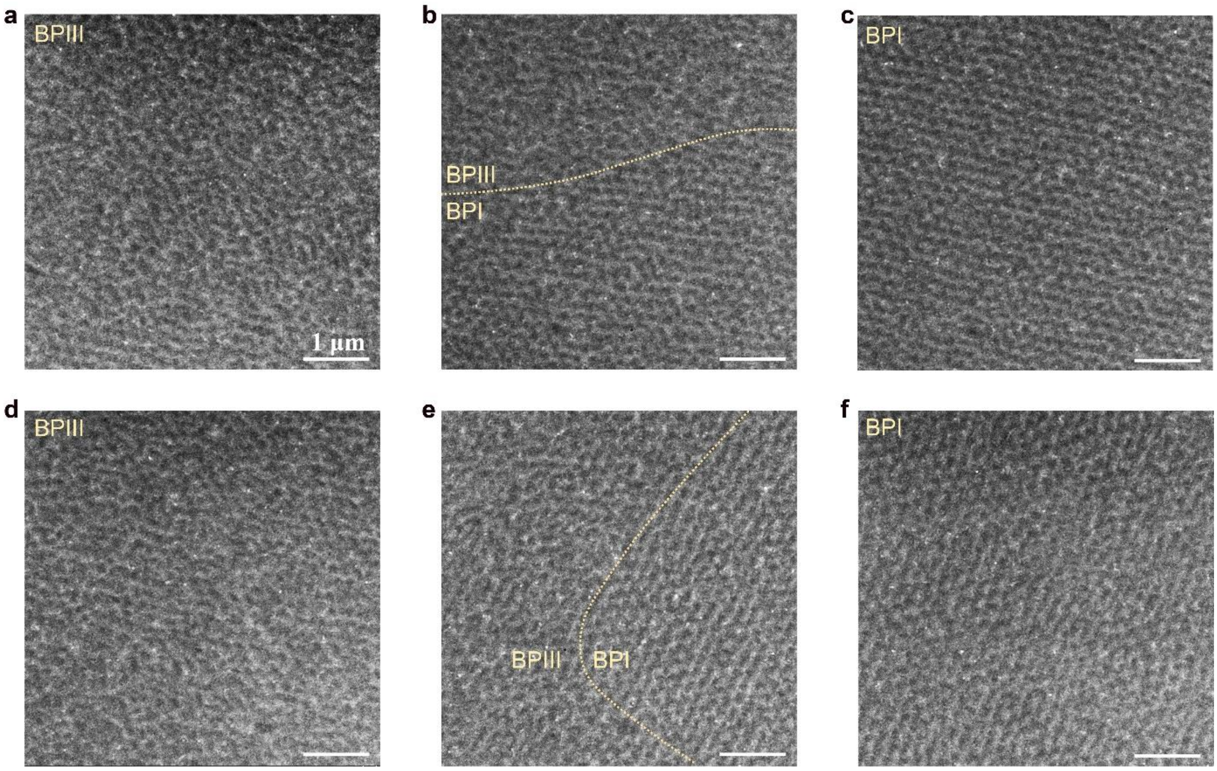

Supplementary Fig. 41 TEM images of the interfaces between BPI$_{\{110\}}$ and BPIII were observed in real-space. a, d BPIII. **b, e** the interface of BPIII and BPI$_{\{100\}}$ (yellow line highlights the interface). **c, f** BPI$_{\{110\}}$.

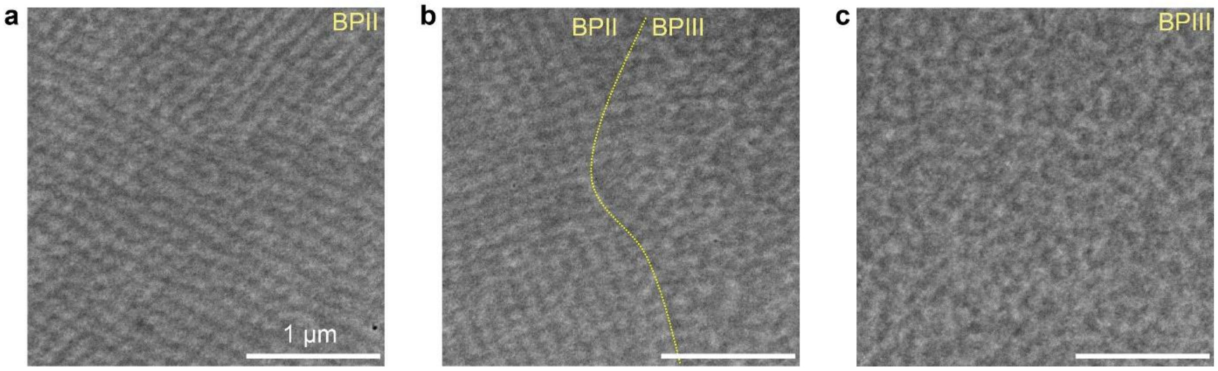

Supplementary Fig. 42 TEM images of the interface between BPII and BPIII in real-space. a BPII$_{\{100\}}$. **b** The interface between BPII$_{\{100\}}$ and BPIII (yellow line highlights the interface). **c** BPIII.

The structures of BPII$_{\{100\}}$ either near the interface or in the bulk are highly ordered. Therefore, the phase transformation occurs within the size range of a unit cell of BPII$_{\{100\}}$ in (b) and without a transition region which may be attributed to that the DTCs diffusion first and then reorganization when phase changes.

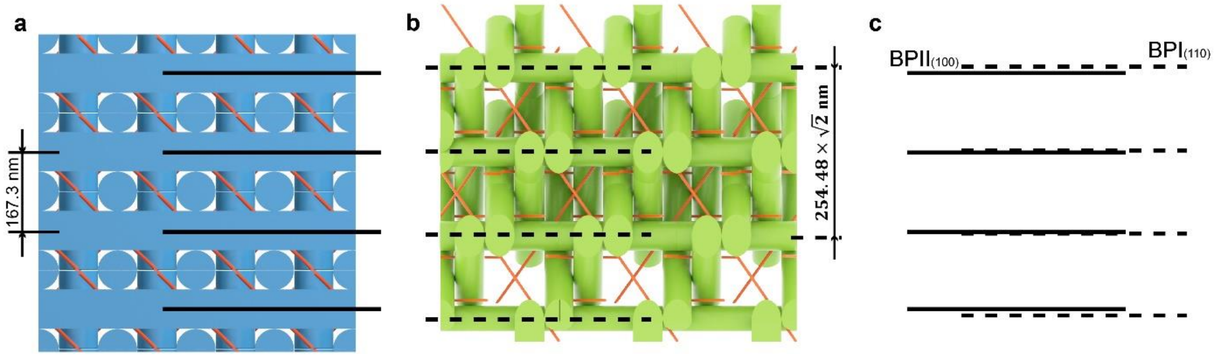

Supplementary Fig. 43 a Model of BPII unit cells with the [100] orientation out-of-plane. The axis of horizontal DTCs is highlighted by black solid lines. **b** Model of BPI unit cells with the [110] orientation out-of-plane. The axis of horizontal DTCs is highlighted by black dash lines. **c** The horizontal oriented DTCs of $BPII_{\{100\}}$ are parallel to that of $BPI_{\{110\}}$. The interval of horizontal DTCs between $BPI_{\{110\}}$ and $BPII_{\{100\}}$ are similar.

Clearly, nearly one-third of the DTCs in $BPI_{\{110\}}$ [horizontal DTCs of $BPI_{\{110\}}$ in (b)] transfer from the horizontal DTCs of $BPII_{\{100\}}$ (a). Since the space between adjacent horizontal DTCs in $BPII_{\{100\}}$ are similar to that of $BPI_{\{110\}}$ (c), predicting the coherent lattice formation between $BPI_{\{110\}}$ and $BPII_{\{100\}}$. Supplementary Figs. 28c_1, c_4, 38b, and 39 show the clear interfaces measured by TEM and the theoretically predicted arrangement of DTCs near the interface between $BPII_{\{100\}}$ and $BPI_{\{110\}}$, showing coherent lattice between $BPII_{\{100\}}$ and $BPI_{\{110\}}$. Furthermore, the 2-Theta values measured by syn-SAXS corresponding to $\{110\}_{BPII}$ and $\{211\}_{BPI}$ are similar (Supplementary Figs. 46c_3, e_3, 47c_1-d_1, 48, 56d_1-d_4), proving coherent of crystal lattice between $\{110\}_{BPII}$ and $\{211\}_{BPI}$ which is an evidence for the thermoelastic martensitic transformation.

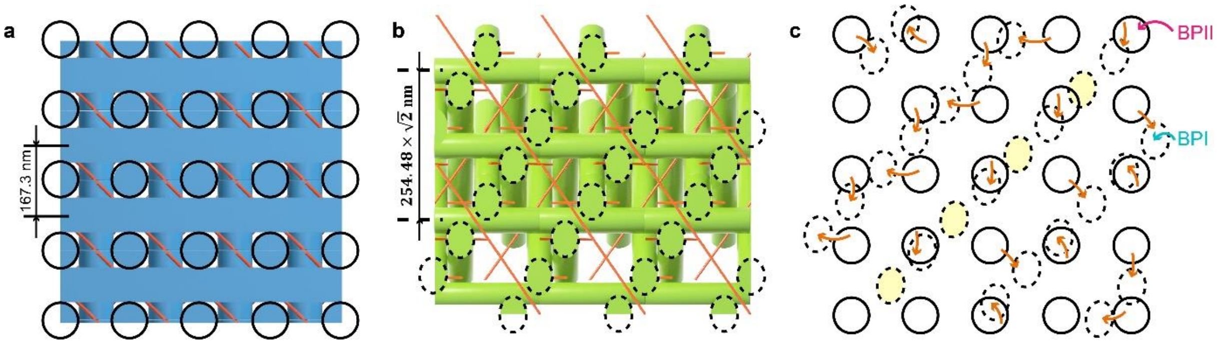

Supplementary Fig. 44 Schematic image of the collective motion of DTCs during the phase transformation from BPII to BPI. a BPII unit cells with the [100] orientation out-of-plane. The solid black circles highlight the position of DTCs with vertical orientation. **b** BPI unit cells with the [110] orientation out-of-plane. The dash black ellipses highlight the position of DTCs with tilt orientation. **c** The relationship between the vertical DTCs of $BPI_{\{110\}}$ and inclined DTCs of $BPII_{\{100\}}$ is evaluated by the quantity and position of DTCs. The orange arrows highlight the predicted tendency of the rearrangement of DTCs during the phase transformation of BPII/BPI.

As shown in the projected models, the vertical DTCs (solid circles) in $BPII_{\{100\}}$ (a) tend to transfer to the inclined DTCs (dash circles) in $BPI_{\{110\}}$ (b). The orange arrows in (c) point out the probable tendency of rearrangement of DTCs during the phase transformation from BPII to BPI. However, the total number of inclined DTCs in $BPI_{\{110\}}$ is more than the vertical DTCs of $BPII_{\{100\}}$ and the extra yellow ellipses shown in (c) may transfer from the horizontal DTCs of $BPII_{\{100\}}$.

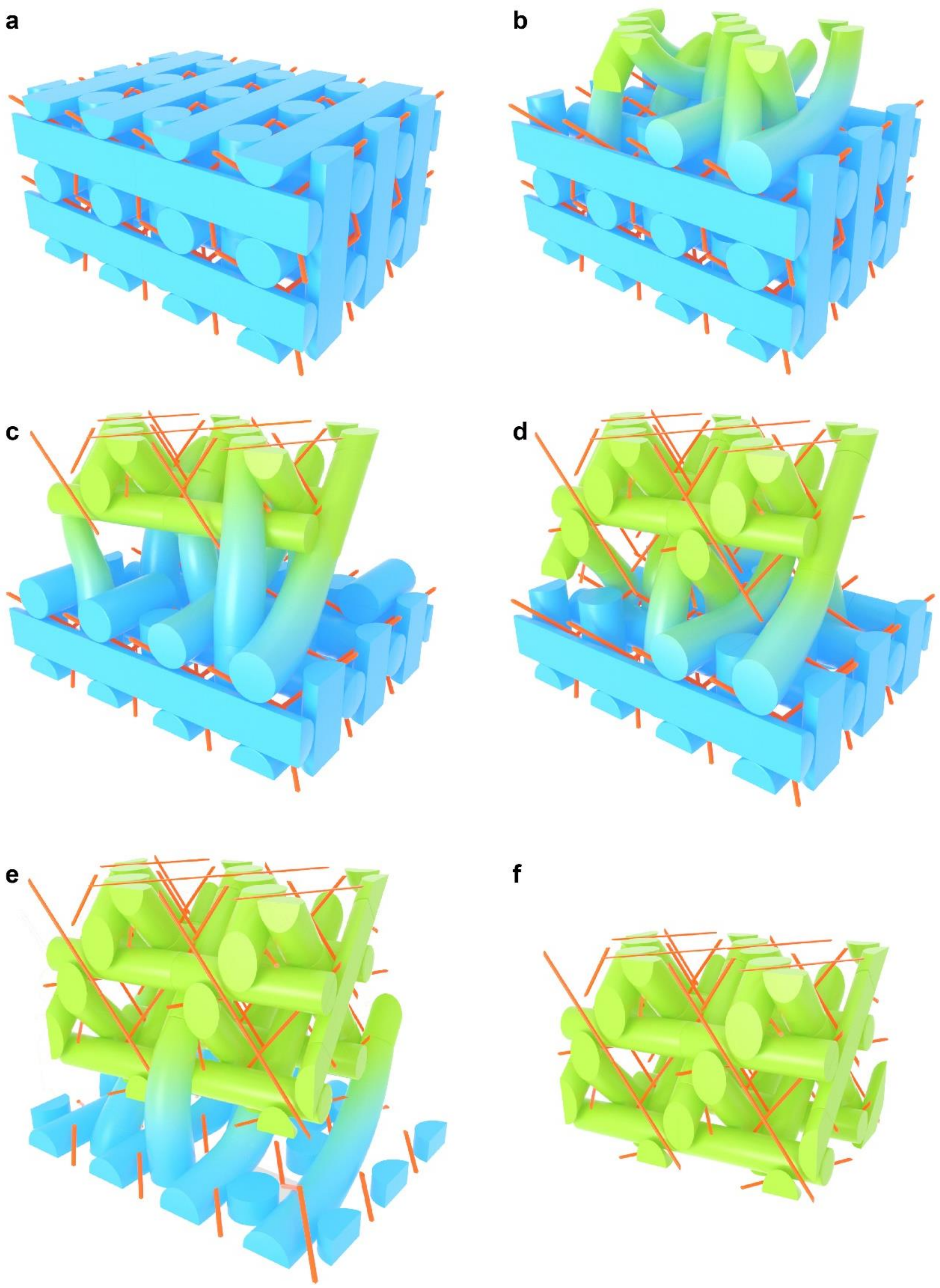

Supplementary Fig. 45 Models highlight the proposed mechanism of the collective move of DTCs during the thermoelastic martensitic transformation from $BPII_{\{100\}}$ with 16 unit cells of (2 × 2× 4) to $BPI_{\{110\}}$,−9.74°. **a** $BPII_{\{100\}}$. **b-e** The process of thermoelastic martensitic transformation from $BPII_{(100)}$ to $BPI_{(110)}$. **f** $BPI_{(110)}$.

Based on the experiment results and analysis shown in Supplemental Figs. 33 and 52-53, the total process of rearrangement of DTCs during the thermoelastic martensitic transformation from BPII$_{\{100\}}$ with 16 unit cells of $(2 \times 2\times 4)$ to BPI$_{\{110\}}$ is shown in Figure 6 (in the main text). Several behavior of the rearrangement of DTCs are confirmed during the thermoelastic martensitic transformation of BPII/BPI: (i) the parent lattice of BPII needs to rotate 9.74°. (ii) nearly one-third of the horizontal DTCs of BPII$_{\{100\}}$ tend to transfer to the horizontal DTCs of BPI$_{\{110\}}$, resulting in the coherent lattice near the interface of BPII/BPI. (iii) the vertical DTCs in BPII$_{\{100\}}$ tend to transfer to the tilt DTCs in BPI$_{\{110\}}$. The total number of tilt DTCs in BPI$_{\{110\}}$ is more than the vertical DTCs of BPII$_{\{100\}}$ .The extra tilt DTCs of BPI$_{\{110\}}$ may transfer from the horizontal DTCs of BPII$_{\{100\}}$.

Supplementary Note 5 Crystal analyses through syn-SAXS characterization of DLPT based on PS-BPLCs

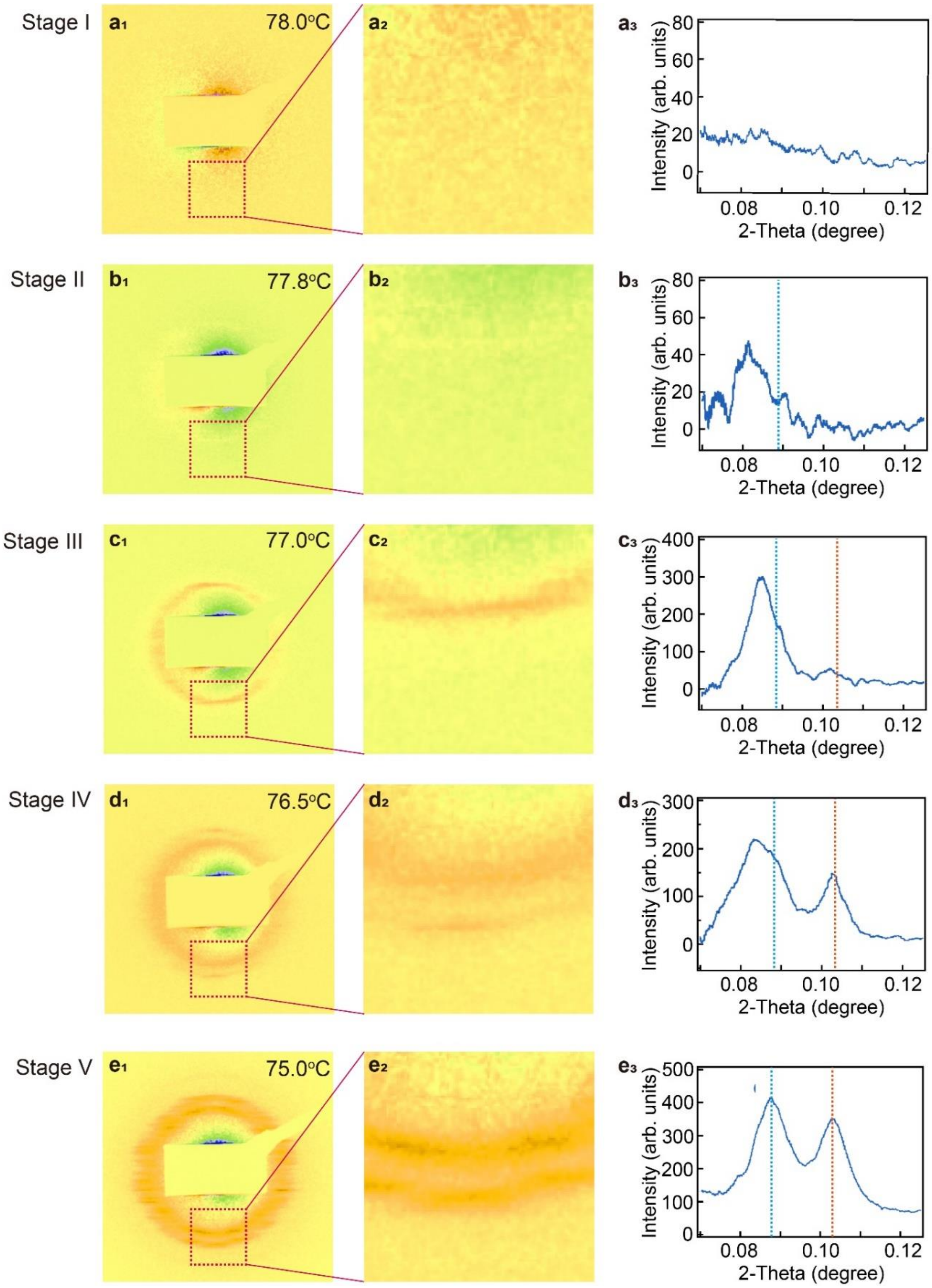

Supplementary Fig. 46 Crystal-crystal transformation during the DLPT measured from polymer-stabilized polydomain BPLCs at distinct stages. a_1-e_1 2D-SAXS patterns with background subtracted. **a_2-e_2** Enlarged images of the area highlighted by red squares in (**a_1-e_1**). **a_3-e_3** 1D-SAXS curves obtained by 90° azimuthal integral from (**a_1-e_1**).

Polydomain PS-BPLCs are characterized by syn-SAXS at each stage which provides a macroscopic view on the crystal-crystal transformation. The 2D-SAXS patterns and corresponded 1D-SAXS curve are shown here. Stage I (a_1-a_3) is BPIII embedded in an isotropic background without diffraction speckle (a_1) or peak in the 1D-SAXS curve (a_3) owing to BPIII has similar symmetry to the isotropic state[9]. Stage II (b_1-b_3) consists of BPIII domains and BPIII/BPII core-shell configurations, giving a weak diffraction ring of BPII in the 2D-SAXS pattern (b_2), and an obvious peak is detected at 2θ=0.0812° in the 1D-SAXS curve (b_3). Stages III (c_1-c_3) composes of the BPIII domains and core-shell configurations of BPIII/BPII, BPIII/BPI, and BPIII/BPII/BPI. In this case, more residual BPIII transfers to BPII which attributes to the intensity increase of BPII at 2θ=0.0847°. Due to the small amount of BPI nucleates in the center of BPII and BPIII domains, a diffraction peak of $\{310\}_{BPI}$ can be observed around 2θ=0.1020° (c_3). Stage IV (d_1-d_3) only composes of BPIII/BPII/BPI core-shell configurations. In this stage, all of BPIII/BPI core-shell configurations have transferred to BPIII/BPII/BPI core-shell configurations where more BPIII transferred to BPII or BPI. Therefore, both diffraction rings of BPII and BPI become stronger (d_2). In the 1D-SAXS curve, the peak belonging to $\{310\}_{BPI}$ is located at 2θ=0.1028° whereas the peak at 2θ=0.085° belongs to the overlapped two peaks of $\{110\}_{BPII}$ and $\{211\}_{BPI}$. The peaks of $\{110\}_{BPII}$ and $\{211\}_{BPI}$ are almost overlapped where the details will be discussed in the following context (Supplementary Figs. 47-48). Stage V indicates a complete transition of the sample from BPIII, BPII to BPI (e_1-e_3), and two diffraction rings can be observed in 2D-SAXS, in which the inner ring originates from $\{211\}_{BPI}$ and the outer ring originates from $\{310\}_{BPI}$. In the 1D-SAXS curve, the peak of $\{310\}_{BPI}$ is located at 2θ=0.1030° whereas the peak located at 2θ=0.0874° belongs to$\{110\}_{BPII}$. The blue and orange dotted lines shown in (a_3-e_3) highlights the center wavelength of two diffraction peaks. Clearly, both peaks of BPI and BPII shift to large angles which represent the shrinkage of the crystal lattice during the phase transition process from Stage I to V. With the emergence of BPI, a newly appeared diffraction ring can be observed in

(c_2) and (d_2). However, when BPII completely transfers to BPI (Stage V), two rings of $\{211\}_{BPI}$ and $\{310\}_{BPI}$ (e_2) are presented in monophasic BPI, proving the overlapping of the diffraction ring of $\{211\}_{BPI}$ and $\{110\}_{BPII}$ which will be further investigated in Supplementary Figures 47-48.

During the phase transition process, the sharp diffraction rings of both BPI and BPII without diffuse scattering are observed, indicating that a sudden reconfiguration without transitional region exists near the interface between BPII and BPI.

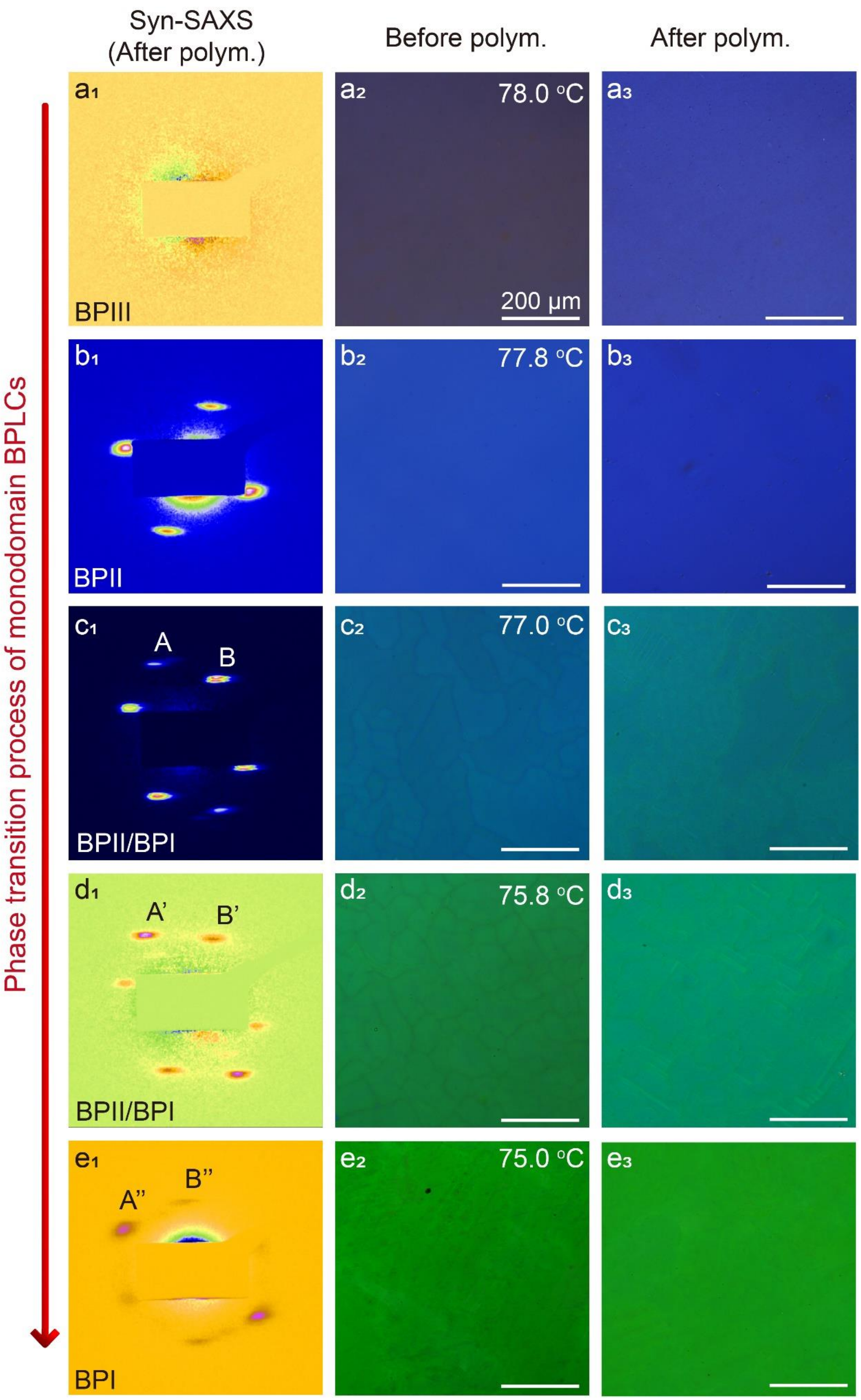

Supplementary Fig. 47 a_1-e_1 Syn-SAXS patterns and POM characterization of monodomain BPLCs (**a_2-e_2**) before and (**a_3-e_3**) after polymer-stabilization.

To further index the diffraction rings during the phase transition process of polydomain BPLCs (Supplementary Fig. 46), syn-SAXS and temperature-dependent POM are performed on monodomain BPLCs (Supplementary Movie 3). During the phase transition process, no diffraction pattern is observed for polymer-stabilized BPIII (a_1-a_3). Four-fold symmetry diffraction speckles are obtained in polymer-stabilized BPII (b_1-b_3). The diffraction speckles labeled with A belongs to the BPI in the hybrid phases of BPI/BPII, which is polymerized at 77.0 ℃ (c_1-c_3). Speckle labeled with A’ is enhanced while speckle labeled with B’ is weakened, owing to the growth of BPI and the gradual disappearance of BPII in a hybrid phase of BPI/BPII polymerized at 75.8 ℃ (d_1-d_3). Speckles labeled with A’’ and B’’ are observed in monodomain BPI polymerized at 75.0 ℃ (e), in which all the BPII in (c-d) has transferred to BPI, indicating the speckles of BPII labeled with B and B’ are overlapped by those of BPI labeled with B’’. Thus, a similar q values and fixed azimuthal angle between BPII and BPI are confirmed according to the gradual fading from B, B’ to B’’ and enhancement from A, A’ to A’’.

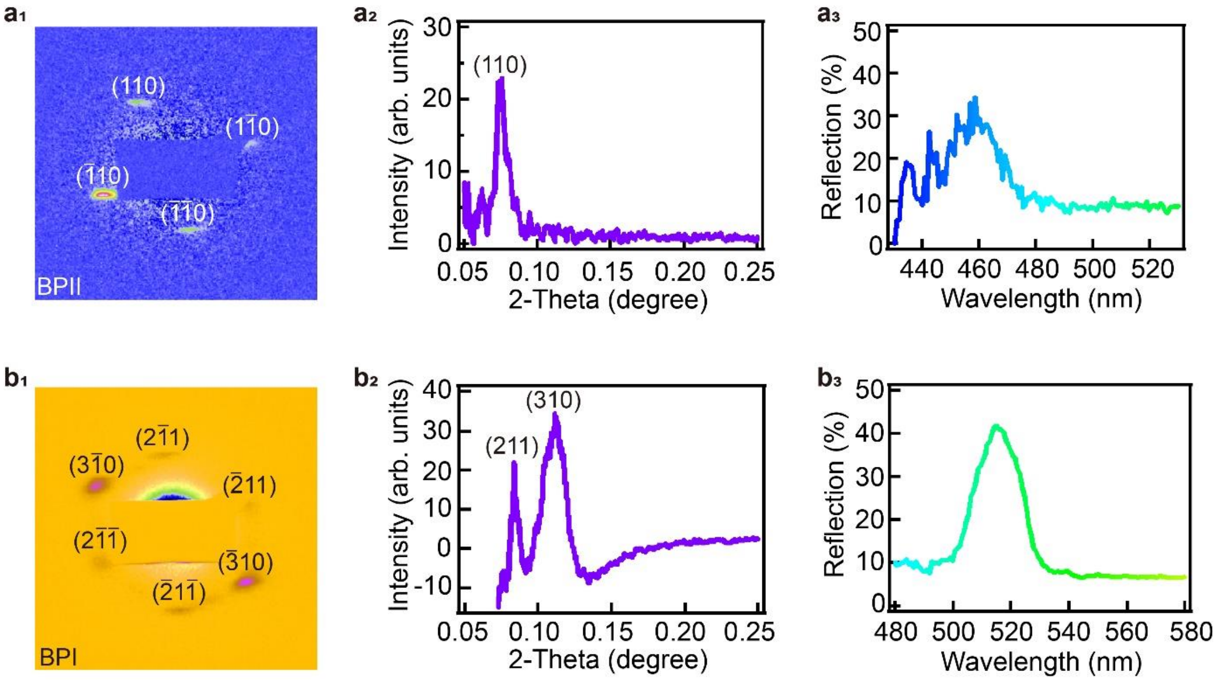

Supplementary Fig. 48 Syn-SAXS results and the corresponding reflection spectra of (a) BPII and (b) BPI. a_1, b_1 2D-SAXS patterns with background subtracted of $BPII_{\{100\}}$ (**a_1**) and $BPI_{\{110\}}$ (**b_1**). **a_2, b_2** 1D-SAXS curves obtained by 90° azimuthal integral from 2D-SAXS patterns of $BPII_{\{100\}}$ (**a_1**) and $BPI_{\{110\}}$ (**b_1**). **a_3, b_3** *In situ* reflection spectra of the same area as the samples used to measure 2D-SAXS patterns of (**a_1**) $BPII_{\{100\}}$ and (**b_1**) $BPI_{\{110\}}$.

Syn-SAXS results and the corresponding reflection spectra of monodomain $BPII_{\{100\}}$ and $BPI_{\{110\}}$ have been measured along the normal direction. Four diffraction spots of $\{110\}_{BPII}$ are observed in (a_1). Systematic extinctions of BPII are observed on $\{h00\}$ when the obtained indexed pattern corresponds to the selection rule: $h = \mathrm{n}$ (n is an odd number). One diffraction peak located at 2θ=0.0746° (a_2) is obtained by 90° azimuthal integral from 2D-SAXS patterns of BPII (a_1). The lattice content of BPII is 167.33 nm calculated by Bragg's equation: $\lambda = 2d_{(hkl)} sin\theta$ (λ: wavelength of synchrotron radiation X-ray which is 1.54 Å; $d_{(hkl)}$: interplanar distance calculated by $d_{(hkl)} = \frac{a}{\sqrt{h^2+k^2+l^2}}$; θ: diffraction angle). The corresponding λ_c of $BPII_{\{110\}}$ is located at 459 nm obtained by *in situ* reflection spectrum (a_3). Six diffraction spots belonging to $\{211\}_{BPI}$ and $\{310\}_{BPI}$ are observed in (b_1). Systematic extinctions of BPI observed on the obtained indexed pattern correspond to the selection rule: $h + k + l = \mathrm{n}$ (n is an odd number) for $\{hkl\}$ and $k + l = \mathrm{n}$ (n is an odd number) for $\{0kl\}$. Two diffraction

peaks are obtained by 90° azimuthal integral from 2D-SAXS patterns of $BPI_{\{110\}}$ (b_1). The diffraction peak belongs to $\{211\}_{BPI}$ is located at 2θ=0.0835° and $\{310\}_{BPI}$ located at 2θ=0.1096° (b_2). The lattice content of BPI calculated from $\{211\}_{BPI}$ (2θ=0.0835°) is 258.87 nm while the crystal lattice calculated from $\{310\}_{BPI}$ (2θ=0.1096°) is 254.48 nm. Owing to lattice constant calculated from $\{310\}_{BPI}$ is located at a higher angle in the 1D-SAXS curve which has a smaller systematical error than that of $\{211\}_{BPI}$, the crystal lattice calculated from $\{310\}_{BPI}$ is more precise. The corresponding *in situ* reflection spectrum of $BPI_{\{110\}}$ indicates the λ_c is located at 518 nm.

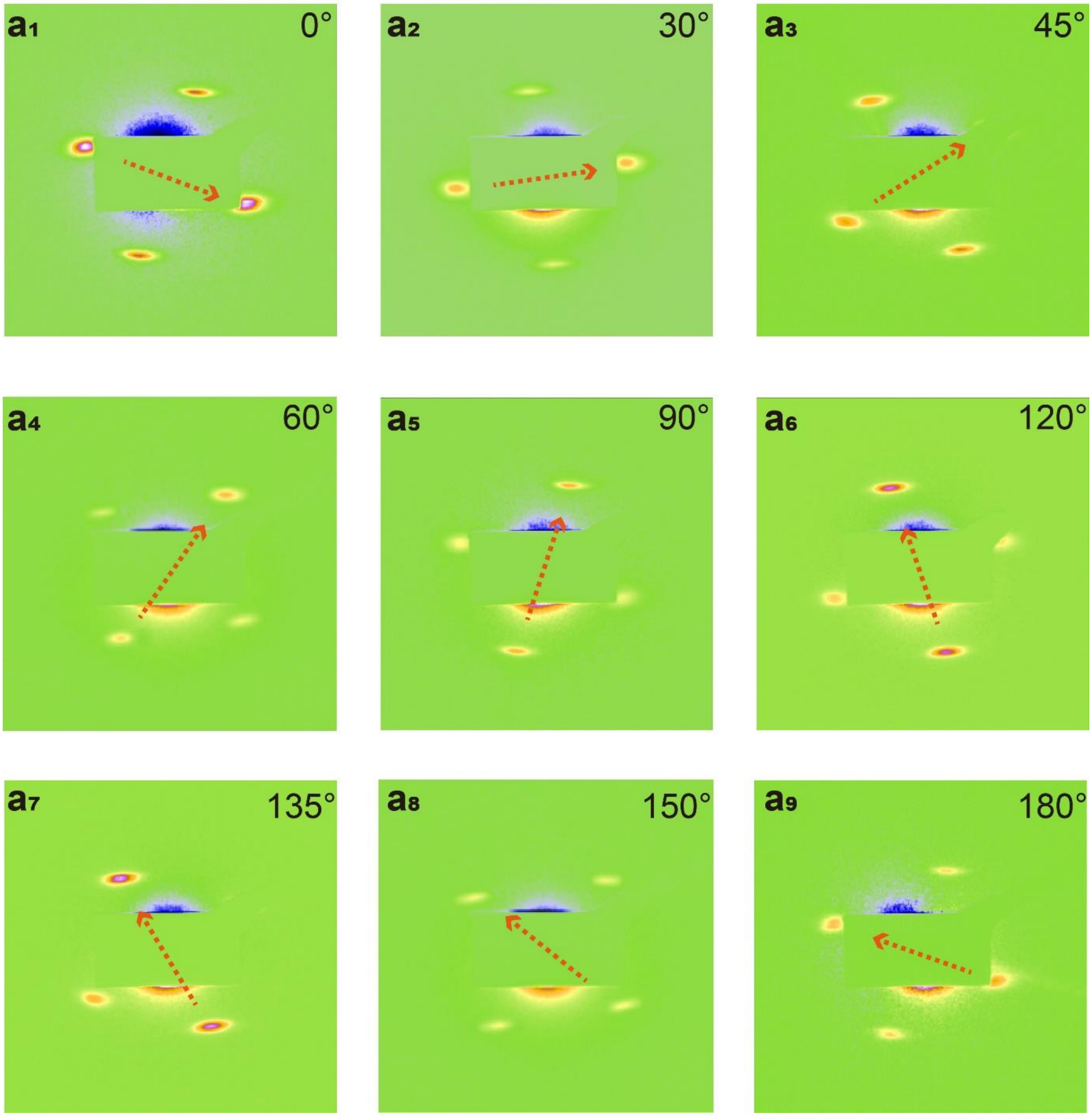

Supplementary Fig. 49 2D-SAXS patterns with background subtracted of monodomain polymer-stabilized BPII. a_1-a_9 The film is rotated from 0° to 180° in {100} crystal plane of BPII. The dotted arrows highlight the angle during rotation.

To further verify the monodomain $BPII_{\{100\}}$ is a single crystal, the angle-dependent syn-SAXS patterns are measured by rotating the sample in $\{100\}_{BPII}$ from 0° to 180°. The four clear diffraction spots of $\{110\}_{BPII}$ rotate with the sample which confirms $BPII_{\{100\}}$ a single crystal.

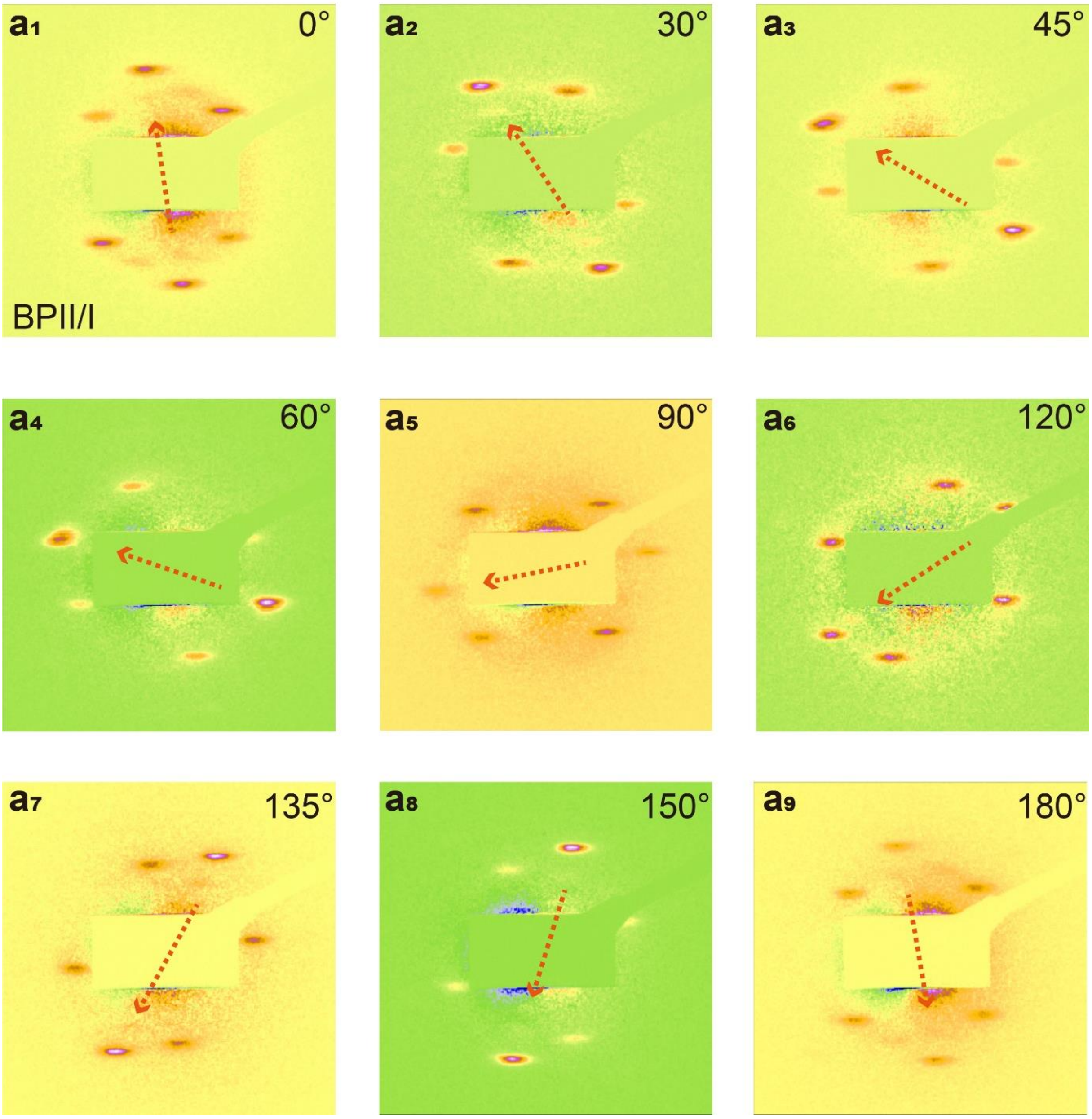

Supplementary Fig. 50 2D-SAXS patterns with background subtracted of hybrid monodomain BPI/BPII. a_1-a_9 The film is rotated from 0° to 180° in $\{110\}_{BPI}$ and $\{100\}_{BPII}$. The dotted arrows highlight the angle during rotation.

To further verify the monodomain BPLCs with hybrid phases of $BPI_{\{110\}}$ and $BPII_{\{100\}}$ obtained during the phase transition process) is a single crystal. The angle-dependent syn-SAXS patterns are measured by rotating the sample in the normal direction from 0° to 180°. The diffraction spots rotate with the film, which confirms both $BPI_{\{110\}}$ and $BPII_{\{100\}}$ in a hybrid phase are single crystals. Due to the temperature gradient along thickness direction in an LC cell on the heat stage, the single crystal of $BPI_{\{110\}}$ and $BPII_{\{100\}}$ mixture should exhibit a layered structure where $BPI_{\{110\}}$ at the bottom is covered by $BPII_{\{100\}}$.

Supplementary Note 6 ARM characterization of phase transition of BPLCs

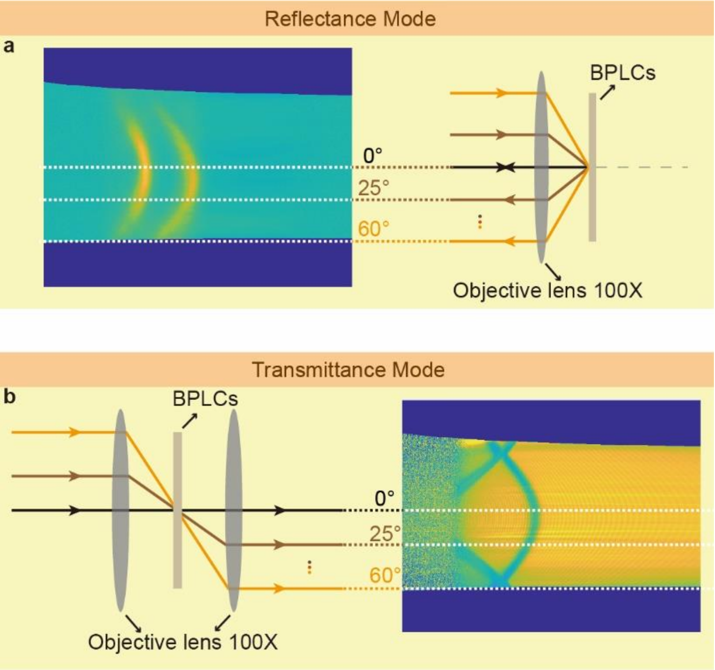

Supplementary Fig. 51 Experiment setup of ARM under the inverted optical microscope in (a) transmission or (b) reflection mode. Schematic views of light paths and the examples of ARM results are shown.

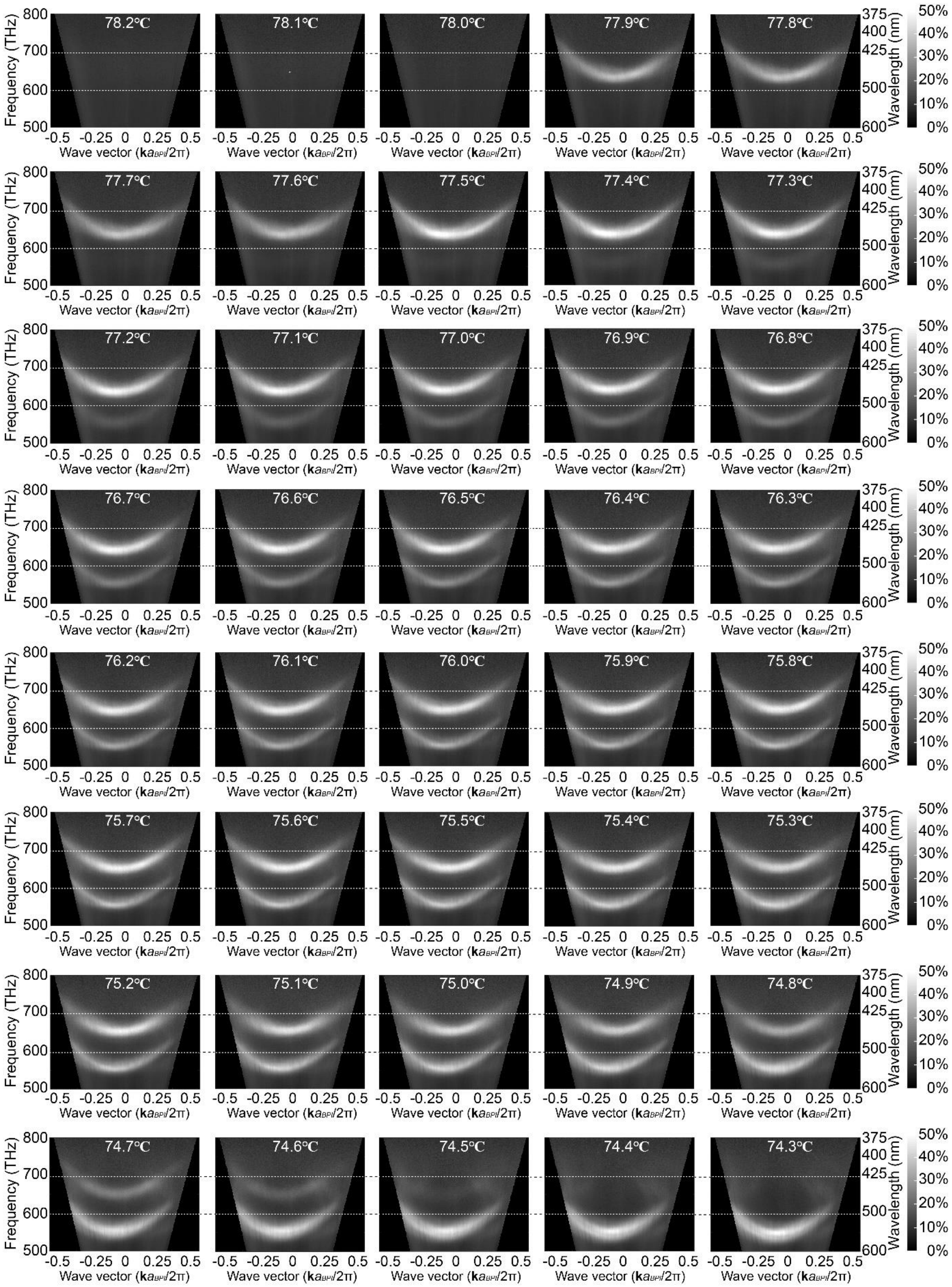

Supplementary Fig. 52 *In situ* observation of the measured reflectance of polydomain BPLCs during the temperature reduction process from Stage I to V. The color scale indicates the reflectivity of the measured area. The colour scale on the right side of each raw is also available for the five spectra in which raw.

Here, *in situ* dynamic track of the polydomain BPLCs is carried out by angle-resolved microspectroscopy (ARM). During the temperature reduction from a clear point to 78.0 ℃, no streak is observed at Stage I (BPIII nuclei in the isotropic background) owing to BPIII is an amorphous structure without bandgaps. When temperature reduces to 77.9 ℃, a bright reflection streak appears around 637 THz (463 nm, normalized frequency of peak near the normal incident angle (f_{nor}) of 0.36), representing the emergence of $BPII_{\{100\}}$ at Stage II. Another streak around 554 THz (541 nm, f_{nor}=0.47) at 77.6 ℃ belongs to $BPI_{\{110\}}$. The coexisting of two streaks represent the coexistence of $BPI_{\{110\}}$ and $BPII_{\{100\}}$. In this case, the center of streak located around 554 (637) THz originates from the [110] ([100]) direction of BPI (BPII). It is confirmed that $\{110\}_{BPI}$ is parallel to $\{100\}_{BPII}$ within the temperature range of 77.3 to 75.6 ℃ owing to the matched center of two streaks, indicating that the orientation of $BPI_{\{110\}}$ crystal lattice can be guided by the orientation of $BPII_{\{100\}}$ crystal lattice. With the further growth of $BPI_{\{110\}}$, the anticrossings of $BPI_{\{110\}}$ become more clear from 74.8 ℃. During the cooling process, the centers of two streaks have a slight mismatch within the temperature range of 75.5 to 75.2 ℃, which is caused by the slight rotation of $\{100\}_{BPII}$ while the orientation of $\{110\}_{BPI}$ remains unchanged. Finally, the streak of $BPII_{\{100\}}$ vanishes at 74.5 ℃ and only one streak of $BPI_{\{110\}}$ is observed at 74.4 ℃ which indicates BPII transfers to BPI completely.

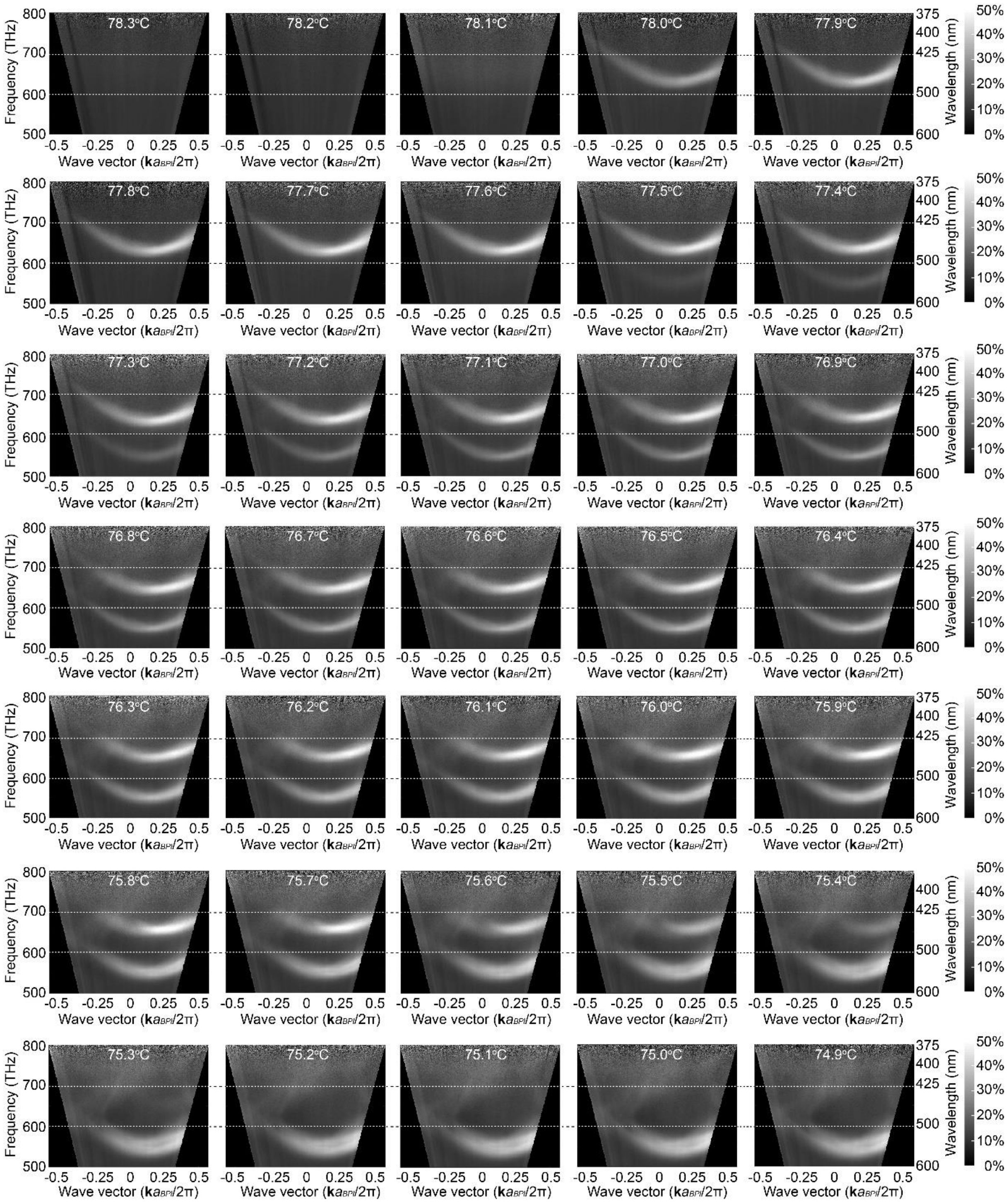

Supplementary Fig. 53 *In situ* observation of the measured reflectance of polydomain BPLCs during the temperature reduction process from Stage I to V, which is similar to Supplementary Fig. 52. The color scale indicates the reflectivity of the measured area. The colour scale on the right side of each raw is also available for the five spectra in which raw.

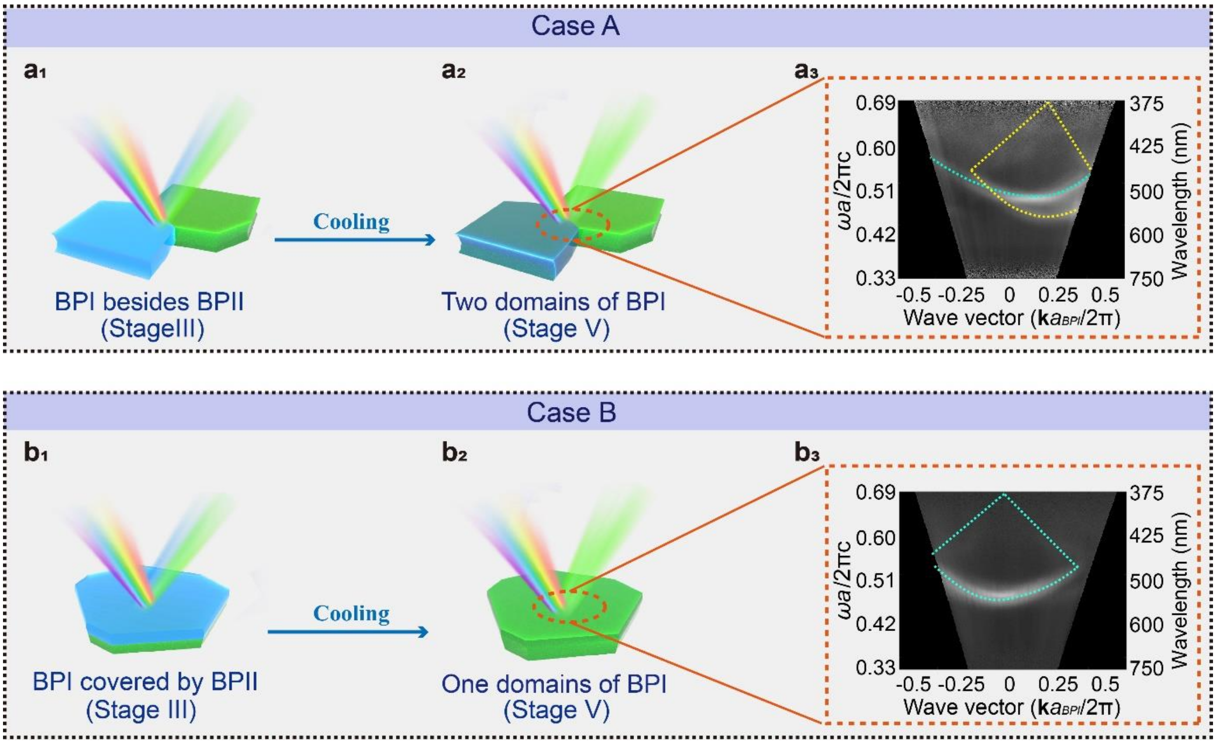

Supplementary Fig. 54 Determination of core-shell configuration during the phase transition process. **a_1-a_3** Case A for prediction of nucleation point of BPI$_{\{110\}}$ domain next to BPII$_{\{100\}}$ domain at Stage III (**a_1**), Stage V (**a_2**) obtained by cooling from (**a_1**), and the contour plot of reflection spectra (**a_3**) obtained from the interface between the two domains of BPI with distinct orientation. **b_1-b_3** Case B for prediction of nucleation point of BPI$_{\{110\}}$ covered by BPII$_{\{100\}}$ at Stage III (**b_1**), Stage V (**b_2**) obtained by cooling from (**b_1**), and the resulted contour plot of reflection spectra (**b_3**).

According to *in situ* measurement results of ARM (reflectance captured at 77.4 ℃ to 74.6 ℃ in Supplementary Fig. 52 and 77.5 ℃ to 75.1 ℃ in Supplementary Fig. 53), two streaks are observed spontaneously indicating that BPI$_{\{110\}}$ and BPII$_{\{100\}}$ coexist in the measured region and the $\{110\}_{BPI}$ is observed approximate parallel to $\{100\}_{BPII}$ during the phase transition process. However, the nucleation and growth sites of BPI are still unclear.

Here, two possible existing cases are presented for the nucleation and growth site of BPI and BPII: side-by-side (Case A) or core-shell (Case B) configuration. Case A represents that BPI and BPII are located side-by-side at Stage III (a_1). To obtain a similar contour plot with two streaks of BPI and BPII (Supplementary Fig. 55) which is obtained by *in situ* ARM (Supplementary Fig. 52), the incident light is required to be at the interface between BPI and BPII (a_1) according to the ARM test principle. When Stage III (a_1) is cooled to Stage V (a_2), BPII domain will transfer to BPI and form a new BPI domain with distinctive orientation, which

is always distinct from the existing BPI domain in (a_1). The position of incident light is kept unchanged during the phase transition process, and the interface of two BPI domains with distinct orientations (a_2) should be measured. Owing to the distinctive reflection streak for particular crystal orientation, two sets of curves overlapped will be predicted (a_3). However, this case is inconsistent with the experimental results (reflectance captured at 74.4 ℃ to 74.3 ℃ in Supplementary Fig. 52 and 75.0 ℃ to 74.9 ℃ in Supplementary Fig. 53), suggesting case A is non-existing in the experiment.

In Case B, the phase transition of BPLCs is supposed to be a core-shell configuration (Case B) where the BPI domain is covered by the BPII domain, and BPI nucleates in the center of BPII. In such a case, to obtain a rational contour plot with two streaks of BPI and BPII (Supplementary Fig. 55) which is obtained by in situ ARM (Supplementary Fig. 52), the incident light can be in the intermediate regions of the core-shell configuration (a_1). After Stage III (b_1) is cooled to Stage V (b_2), BPII will transfers to BPI and forms only one BPI domain, achieveing the same orientation as the BPI domain existed in Stage III (b_1). Since the position of incident light is maintained at the same point in (b_1), only one BPI domain can be measured, exhibiting only one sharp streak observed at Stage V (b_3). Due to the agreement between Case B and experiment results in core-shell configuration (b_3) (reflectance captured at 74.4 ℃ to 74.3 ℃ in Supplementary Fig. 52 and 75.0 ℃ to 74.9 ℃ in Supplementary Fig. 53), the core-shell configuration of BPI covered by BPII can be confirmed by ARM measurement during *in-situ* dynamic track of the phase transition process.

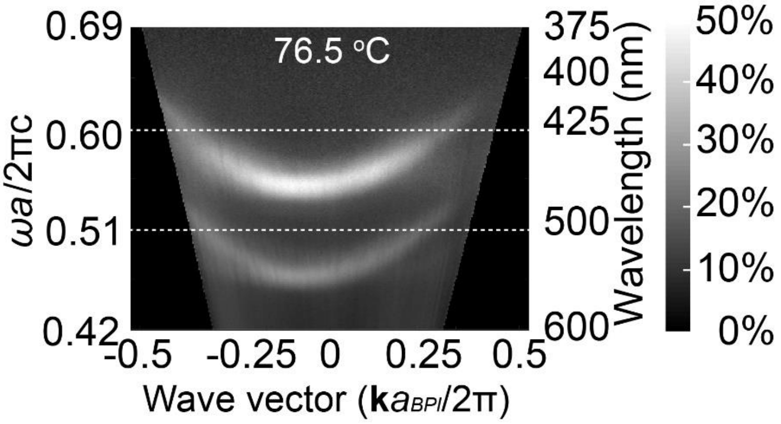

Supplementary Fig. 55 Contour plots of measured reflectance of Case B at Stage III of Supplementary Fig. 54. The color scale indicates the reflectivity of the measured area.

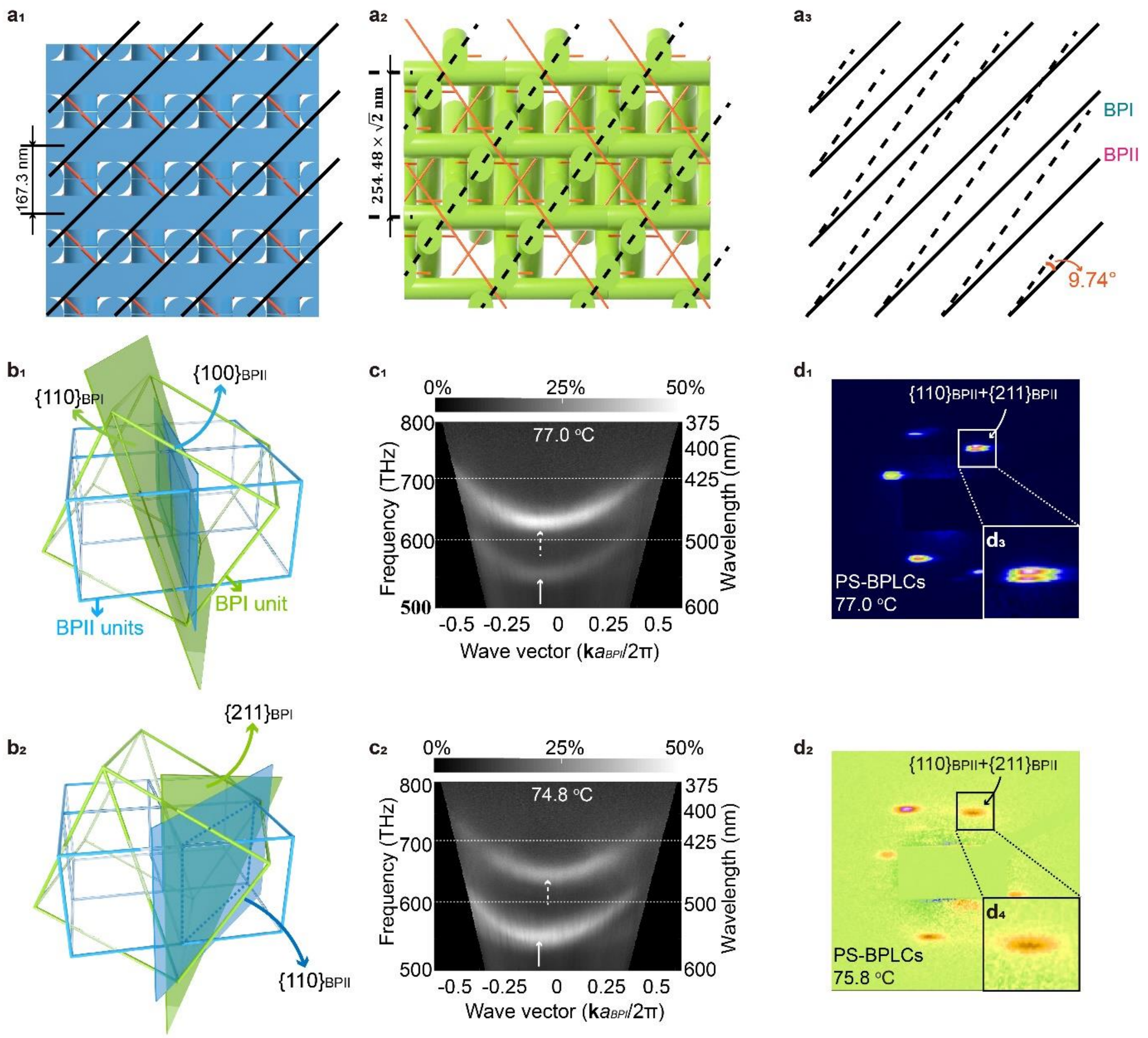

Supplementary Fig. 56 Lattice orientation relationships between BPI and BPII experienced a thermoelastic martensitic transformation. $\mathbf{a_1}$ BPII unit cells with the [100] orientation out-of-plane. The black solid lines connect the centers of DTCs with vertical orientation in BPII. $\mathbf{a_2}$ BPI unit cells with the [110] orientation out-of-plane. The black dash lines connect the centers of DTCs with tilt orientation in BPI. (a_3) Schematic of the angle between the solid lines of BPII and the dash lines of BPI which is 9.74°. $\mathbf{b_1}$ {100} crystal plane of BPII ($\{100\}_{BPII}$) is approximately parallel to {110} crystal plane of BPI ($\{110\}_{BPI}$) while there is a small angle between them. $\mathbf{b_2}$ Schematic illustration of the orientation relationship between BPI and BPII where $\{110\}_{BPII}$ is parallel to $\{211\}_{BPI}$. $\mathbf{c_1}$ ARM result of BPLCs captured at 77.0 °C (Stage III) exhibit a well-matched dip between the $\{110\}_{BPI}$ streak with $\{100\}_{BPII}$ streak. The arrows highlight the centers of the streaks. The color scale indicates the reflectivity of the measured area. $\mathbf{c_2}$ ARM result of BPLCs captured at 74.8 °C (Stage IV) exhibits a slightly mismatched center of the $\{110\}_{BPI}$ streak in relation to $\{100\}_{BPII}$. As the center of $\{110\}_{BPI}$ streak does not shift, the mismatch may be caused by the rotation of $\{100\}_{BPII}$, resulting in a small angle between the approximately paralleled $\{100\}_{BPII}$ and $\{110\}_{BPI}$. The arrows highlight the centers of the streaks. $\mathbf{d_1}$ Syn-SAXS pattern measured from the BPLCs with $BPI_{\{110\}}$ and $BPII_{\{100\}}$ in coexistence, which is polymer-stabilized at 77.0 °C. $\mathbf{d_2}$ Syn-SAXS pattern measured from the BPLCs with $BPI_{(110)}$ and $BPII_{(100)}$ in coexistence, which is polymer-stabilized at 75.8 °C. $\mathbf{d_3}$ The speckles of $BPI_{\{211\}}$ and $BPII_{\{110\}}$ are relatively close but do not overlap, indicating that the $\{110\}_{BPII}$ is approximately parallel to $\{211\}_{BPI}$ with a small angle at an early stage during the phase transition process. $\mathbf{d_4}$ The speckles of $\{211\}_{BPI}$ and $\{110\}_{BPII}$ are well overlapped, indicating that $\{110\}_{BPII}$ and $\{211\}_{BPI}$ are parallel.

As shown in (a_1-a_2), the black solid lines connect the center of DTCs with vertical orientation in $BPII_{\{100\}}$ while the black dash lines connect the center of DTCs with tilt orientation in $BPI_{\{110\}}$. There is an angle of 9.74° (=arccos$\frac{\sqrt{3}}{3}$-45°) between the black solid lines highlighted in $BPII_{\{100\}}$ (a_1) and the black dash lines shown in $BPI_{\{110\}}$ (a_2) is observed in the projected models (a_3), predicting that an angle of 9.74° between $BPI_{\{110\}}$ and $BPII_{\{100\}}$ is required during the thermoelastic martensitic transformation. It is hypothesized that the vertical DTCs in $BPII_{\{100\}}$ tend to form the vertical DTCs in $BPI_{\{110\}}$ during the cooling process. Since the molecules are relatively free to move, the diffusionless fuse and merge of DTCs[2] rearrange through local molecular twist and reorientation. When the vertical DTCs in $BPII_{\{100\}}$ transfer to the tilted DTCs in $BPI_{\{110\}}$, the molecular of DTCs in $BPII_{\{100\}}$ can twist the smallest angle to form DTCs in $BPI_{\{110\}}$ which is an energy favor process. The rotation of the azimuthal angle is further proved by syn-SAXS and ARM results (c-d).

It is observed that the dips of $BPI_{\{110\}}$ and $BPII_{\{100\}}$ streaks are well-matched at an early stage [77.0 ℃ in (c_1)] of the phase transformation, proving that $\{110\}_{BPI}$ //$\{100\}_{BPII}$. When the phase transition is at the last stage [(74.8 ℃ in (c_2)], a small deviation of the dips is observed, resulting in a small angle of 9.74° appears between $\{110\}_{BPI}$ and $\{100\}_{BPII}$ (b_1). In this case, the $\{211\}_{BPI}$ //$\{110\}_{BPII}$ is predicted (b_2). It can be seen that the dip of $BPI_{\{110\}}$ does not change while that of $BPII_{\{100\}}$ has rotated which means the rotations of the parent lattice are required during the thermoelastic martensitic transformation.

Simultaneously, the rotation of the parent BPII phase and the resulted $\{211\}_{BPI}$ //$\{110\}_{BPII}$ can be confirmed by the syn-SAXS results. At an early stage of phase transition, just an approximately parallel relationship between $\{211\}_{BPI}$ and $\{110\}_{BPI}$ can be observed, corroborating by close but not overlapping speckles shown in (d_1 and d_3). However, the $BPII_{\{100\}}$ rotates during the phase transformation, resulting in $\{110\}_{BPII}$ parallels with $\{211\}_{BPI}$ (d_2 and d_4) which is confirmed by the well overlapping of speckles originated from $\{211\}_{BPI}$ and

$\{110\}_{BPII}$ in a monodomain PS-BPLCs hybrid with $BPI_{\{110\}}$ and $BPII_{\{100\}}$. Besides, the 2-Theta values corresponding to $\{110\}_{BPII}$ and $\{211\}_{BPI}$ are similar (Supplemental Figs. 46e_3-f_3, 47c_1-d_1, and 48), providing the coherent of crystal lattices between $\{110\}_{BPII}$ and $\{211\}_{BPI}$ which is an evidence for the thermoelastic martensitic transformation.

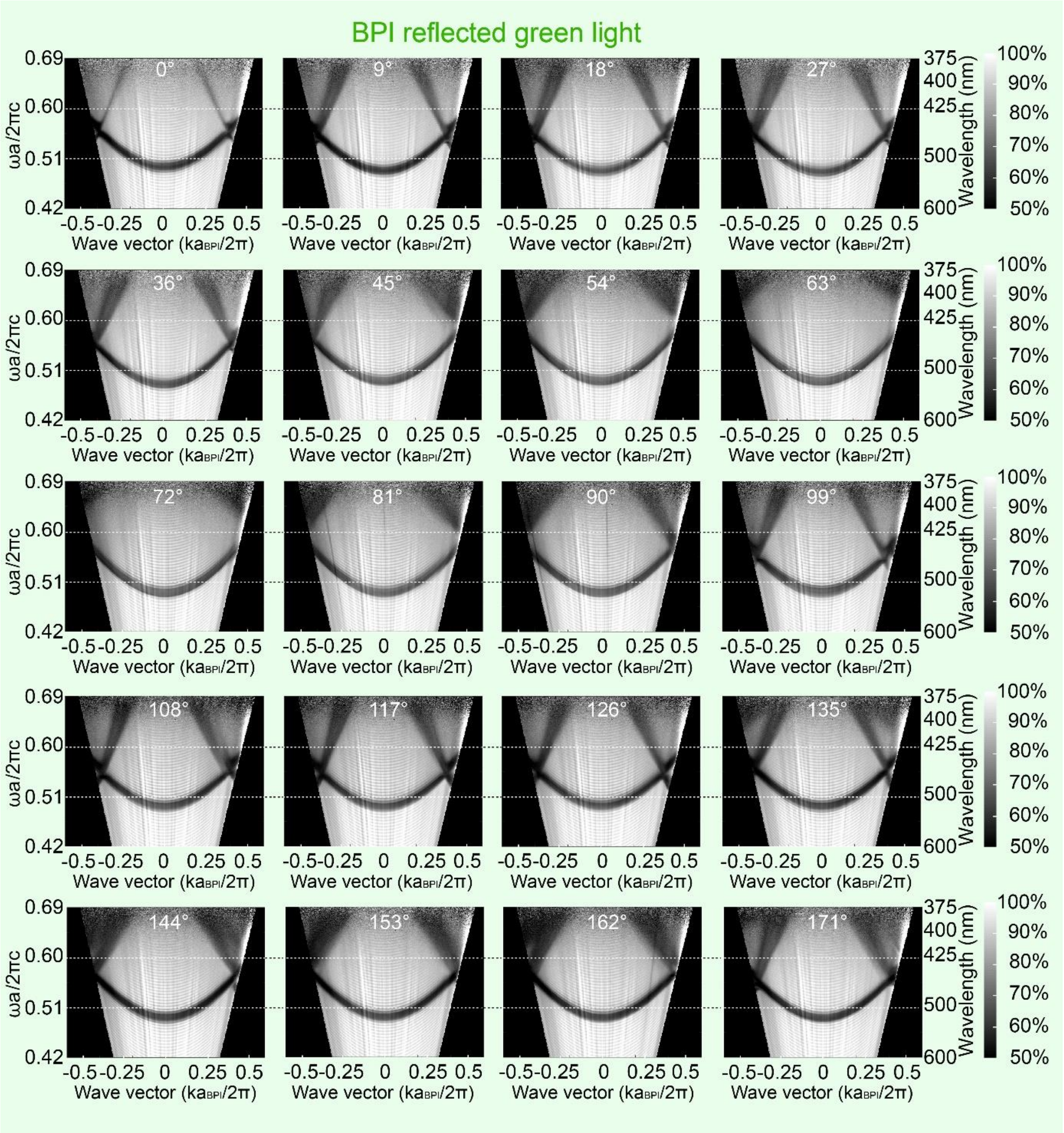

Supplementary Fig. 57 Contour plots of measured transmittance by rotating the monodomain $BPI_{\{110\}}$ (reflected green light) film from 0° to 171° along [110] direction. The color scale indicates the transmissivity of the measured area. The colour scale on the right side of each raw is also available for the four spectra in which raw.

It is found that the anticrossings of streaks moving inward and outward periodically on the spectra when we rotate the azimuthal incident angle of the light irradiated onto BPI which reflected green light. The shapes of the streaks indicate that the normal incident angle corresponds to $\overline{\Gamma \mathrm{N}}$ ([110]) direction which is confirmed by TEM (Supplementary Fig. 31). When the rotation angle of the sample is 126°, a minimum incident angle is observed where anticrossings appear in normalized wave vector $\frac{\mathbf{k}a}{2\pi} = Approx.\ \pm 0.41\ \frac{2\pi}{a_{\mathrm{BPI}}}$ and the

corresponding incident angle is the non-high-symmetry-point $L_{\perp}$ (the perpendicular foot of Γ on $\overline{\mathrm{HP}}$). When sample is rotated to 162°, one maximum is observed where anticrossings appear in normalized wave vector $\frac{\mathbf{k}a}{2\pi} = Approx \pm 0.50 \frac{2\pi}{a_{\mathrm{BPI}}}$ with the azimuthal angle shall correspond to $\overline{\mathrm{NP}}$. The other maximum of the anticrossing angle exceeds the observable range when the sample is rotated to 72°, but we can still determine that azimuthal angle as the $\overline{\mathrm{NH}}$ direction.

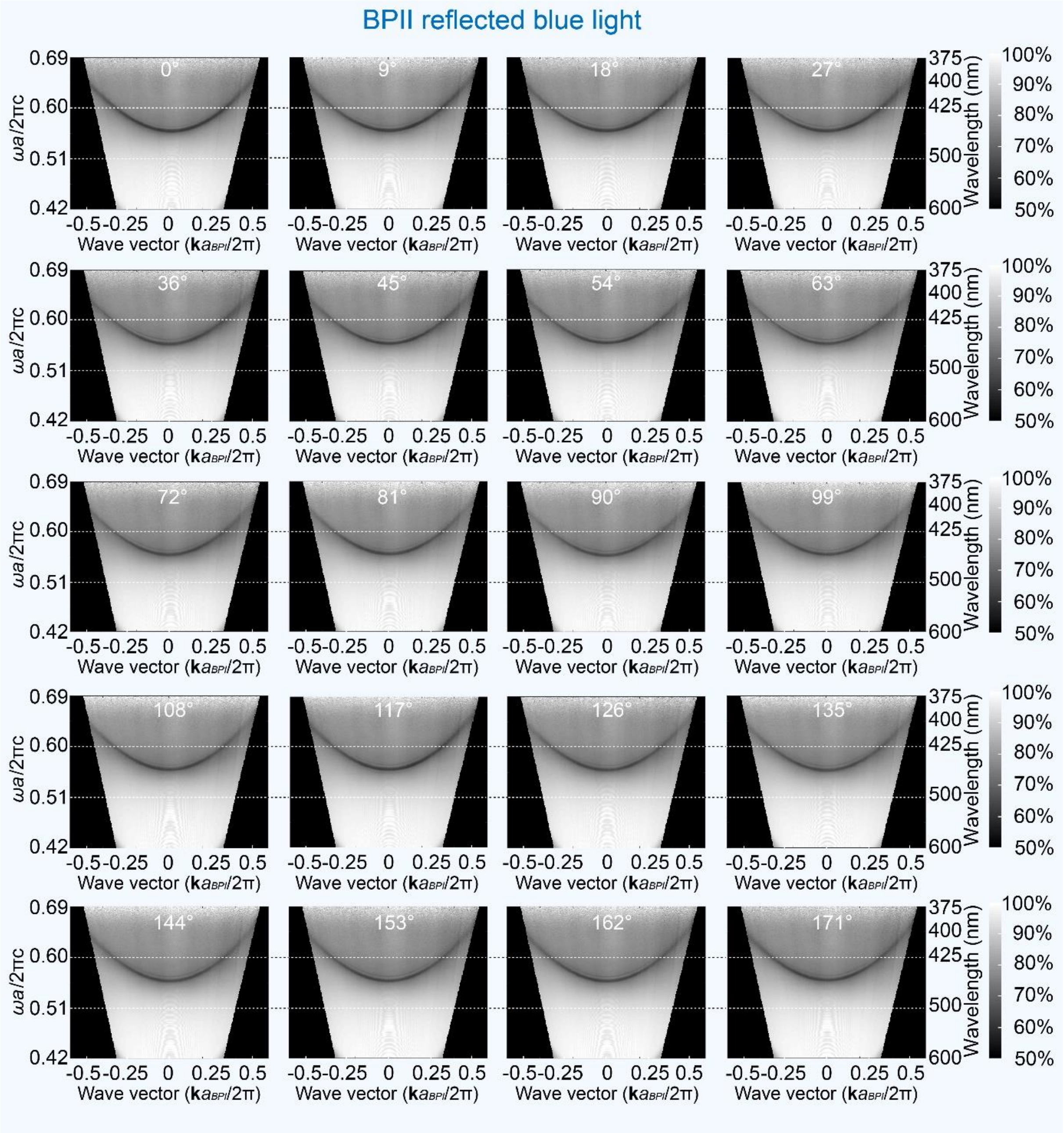

Supplementary Fig. 58 Contour plots of measured transmittance by rotating the monodomain BPII$_{\{100\}}$ (reflected blue color) film from 0° to 171° along [100] direction. The color scale indicates the transmissivity of the measured area. The colour scale on the right side of each raw is also available for the four spectra in which raw.

The normal angle of single-domain polymer-stabilized BPII$_{\{100\}}$ shall be the $\overline{\Gamma X}$ ([100]) direction which is confirmed by TEM (Supplementary Fig. 27). Because of the limited measurement range (Supplementary Fig. 64), the shapes of the streak in transmittance spectra are almost the same during varying the azimuthal angles. Thus, the crystallographical orientations of BPII$_{\{100\}}$ cannot be recognized through ARM.

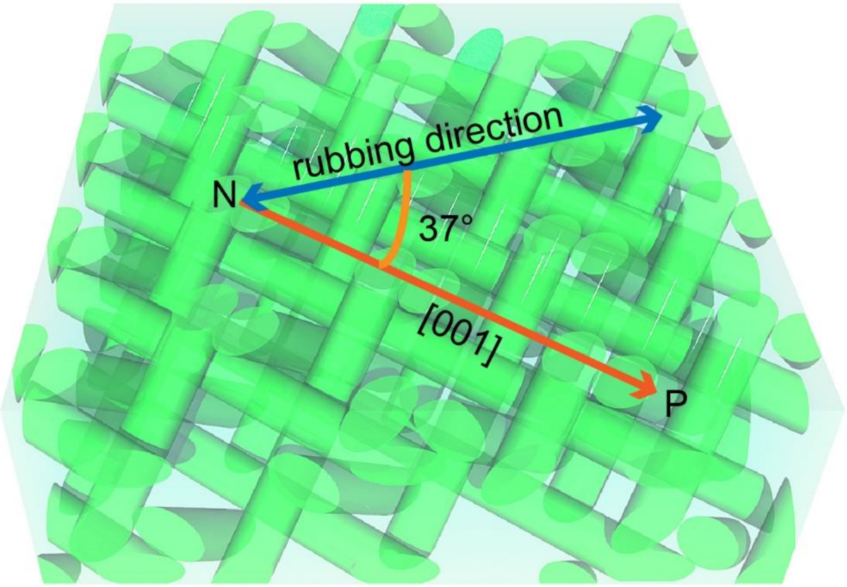

Supplementary Fig. 59 Schematic illustration of the angle between $\overline{\mathrm{NP}}$ direction and rubbing direction.

The angle between rubbing direction and $\overline{\mathrm{NP}}$ direction ([001] of BPI) is measured as 37°. Thus, the azimuthal angle and orientation of BPI can be determined by rubbing direction. The vector shown in the schematic image approximately coincides with the [001] in-plane orientation of the single-crystal $\mathrm{BPI}_{\{110\}}$ domain.

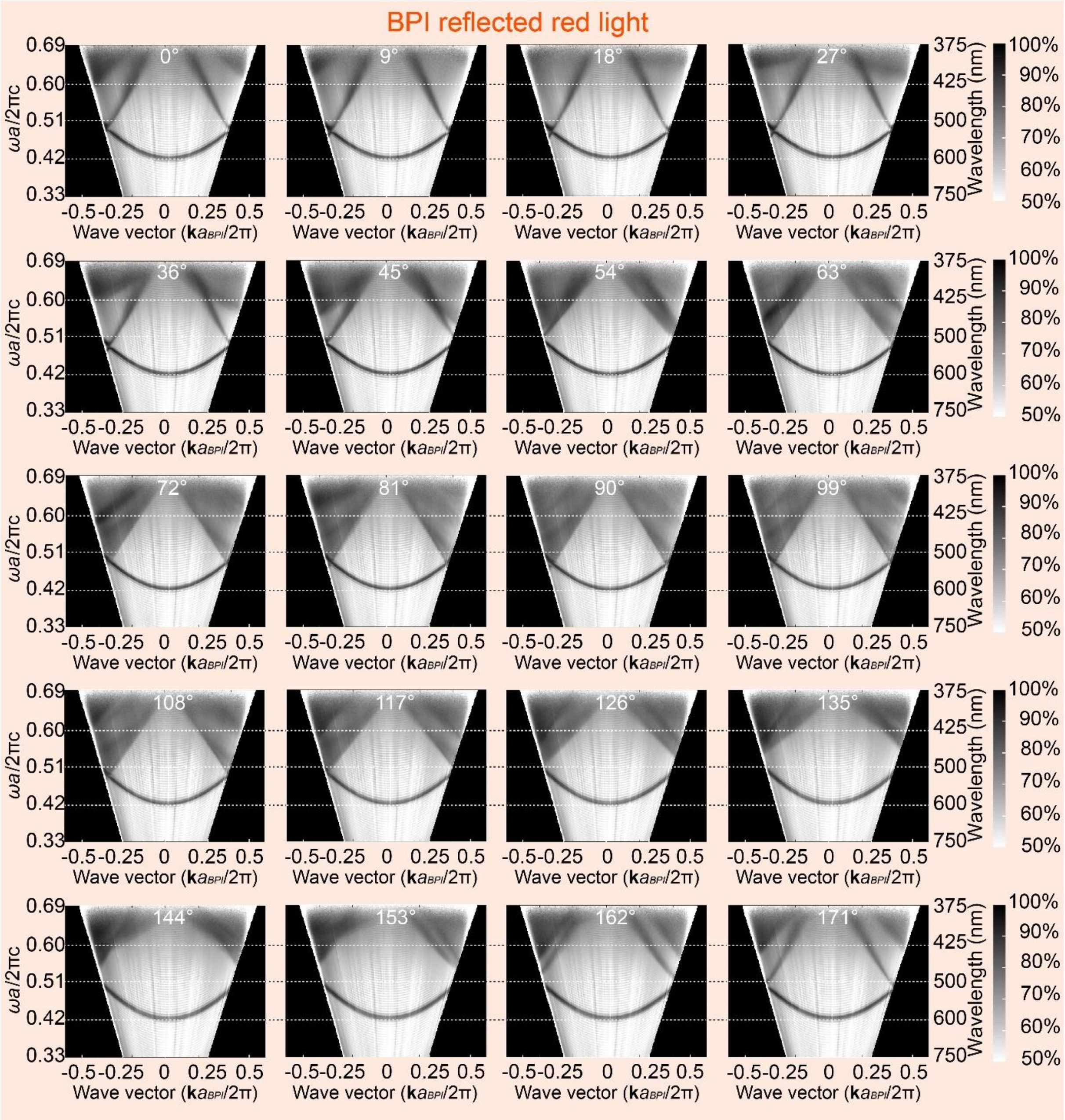

Supplementary Fig. 60 Contour plots of measured transmittance by rotating the monodomain BPI$_{\{110\}}$ (reflected red color) film from 0° to 171° along [110] direction. The color scale indicates the transmissivity of the measured area. The colour scale on the right side of each raw is also available for the four spectra in which raw.

Similar to BPI$_{\{110\}}$ reflected green light (Supplementary Fig. 57), the anticrossings of streaks moving inward and outward periodically on the spectra when the azimuthal incident angle of the light irradiated onto BPI rotated from 0° to 171°. The shape of the streak indicates that the normal incident angle corresponds to the $\overline{\Gamma N}$ ([110] direction). When the rotation angle of the sample is close to 90°, the minimum incident angle is observed where anticrossings

appear in normalized wave vector $\frac{\mathbf{k}a}{2\pi}Approx\pm0.35$ and the corresponding incident angle is the non-high-symmetry-point $L_{\perp}$ (the perpendicular foot of $\mathbf{\Gamma}$ on $\overline{\mathrm{HP}}$). When the sample is rotated to 54°, one maximum angle is observed where the azimuthal angle shall correspond to $\overline{\mathrm{NP}}$. The other maximum of the anticrossing angle also exceeds the observable range when the sample is rotated to 144°, but we can still determine that azimuthal angle as the $\overline{\mathrm{NH}}$ direction.

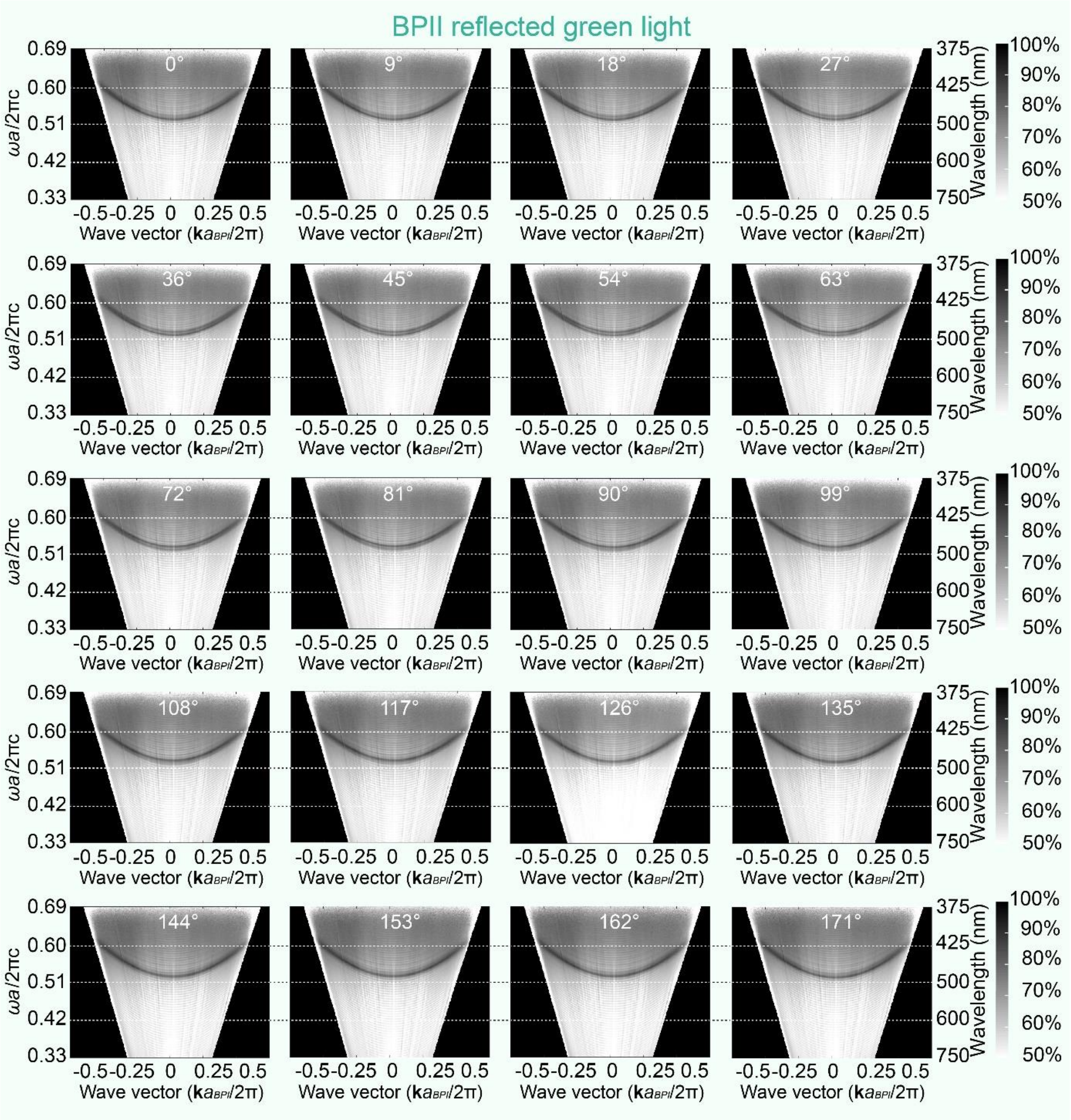

Supplementary Fig. 61 Contour plots of measured transmittance by rotating the monodomain BPII$_{\{100\}}$ (reflected green color) film from 0° to 171° along [100] direction. The color scale indicates the transmissivity of the measured area. The colour scale on the right side of each raw is also available for the four spectra in which raw.

BPII$_{\{100\}}$ film reflects green light was measured when the azimuthal incident angle of the light irradiated onto BPI is rotated from 0° to 171°. The photonic bandgap is located at 490.8 nm. Due to the limited measurement range, the transmittance spectra are all of the same among distinct azimuthal angles similar to BPII$_{\{100\}}$ reflected blue light (Supplementary Fig. 58). Thus, the crystallographical orientations of BPII$_{\{100\}}$ cannot be recognized through ARM.

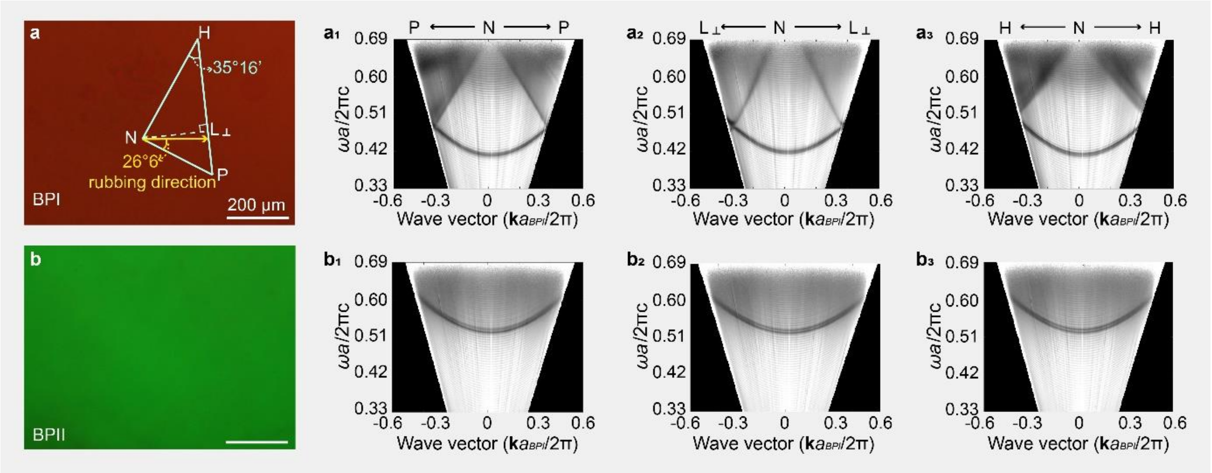

Supplementary Fig. 62 The relationship between rubbing direction and crystal orientation. a POM image of a typical texture of monodomain BPI$_{\{110\}}$ (reflected red light). The light blue line represents the detect direction. **a1, a2, a3** Contour plots of measured transmittance of BPI along the $\overline{NP}$, $\overline{NL_{\perp}}$ and $\overline{NH}$ orientations. **b** is a POM image of a typical texture of monodomain BPII$_{\{100\}}$ (reflected green light). **b1, b2, b3** Contour plots of measured transmittance of BPII$_{\{100\}}$.

Experimental observation confirms that both the orientation of crystal planes and azimuthal orientations of cubic can be controlled by the rubbing direction of PI alignment layers and detected by the ARM measurement. Due to only one typical band structure is detected for crystal lattice with a particular azimuthal orientation, these results suggest that the role of the rubbed polyimide layers goes beyond merely forming single-crystal BPLCs: azimuthal orientation of BCC (BPI) can be determined by the rubbing direction of alignment layers.

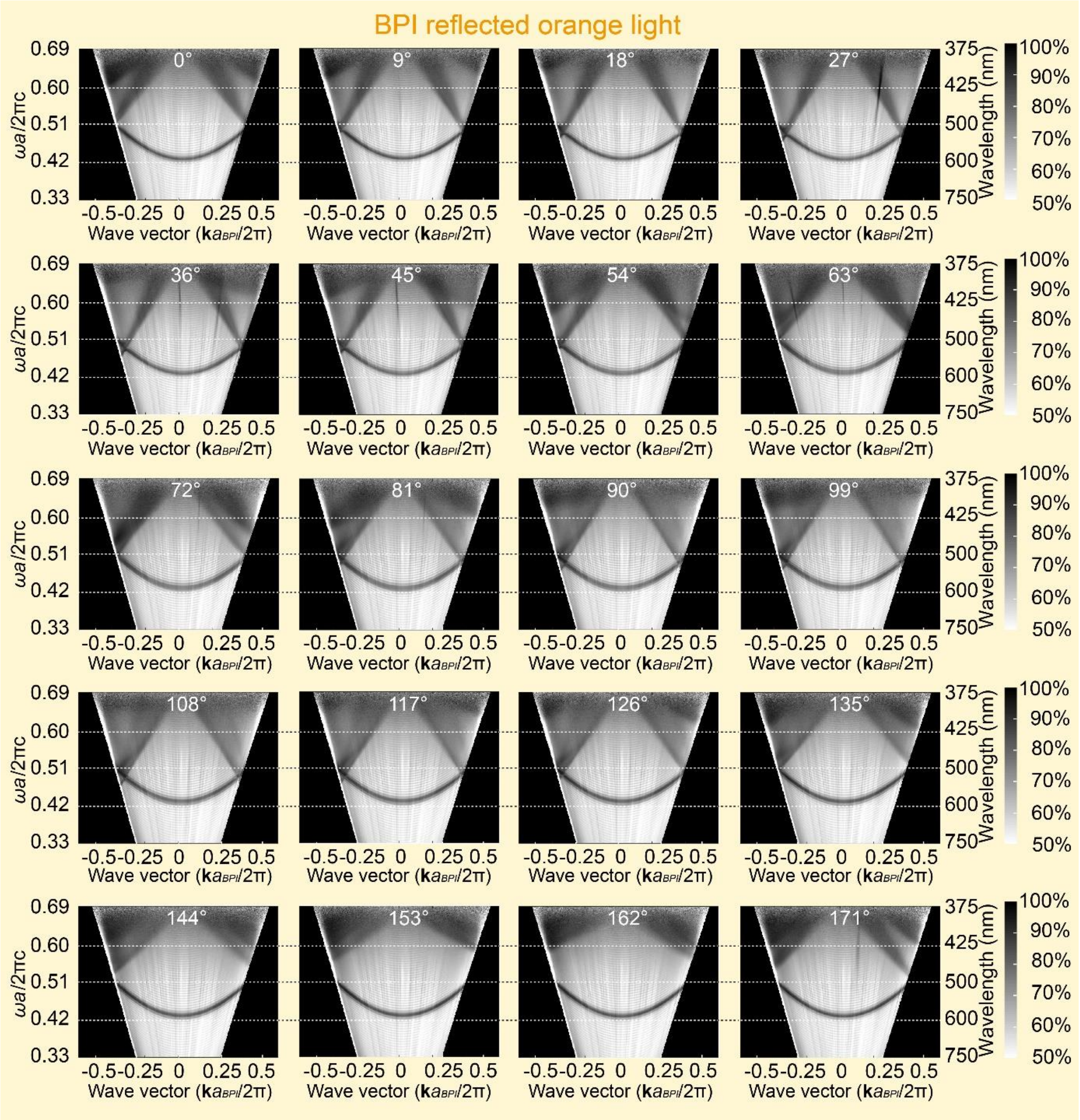

Supplementary Fig. 63 Contour plots of measured 2D transmittance by rotating the monodomain $BPI_{\{110\}}$ film (reflected orange light) from 0° to 171° along [110] direction. The color scale indicates the transmissivity of the measured area. The colour scale on the right side of each raw is also available for the four spectra in which raw.

ARM is performed on monodomain $BPI_{\{110\}}$ film which is reflected orange light. The anticrossings move inward and outward periodically on the streak patterns when the azimuthal incident angle of the light irradiated onto BPI is rotated from 0° to 171°. The shape of the streak indicates that the normal direction corresponds to $\overline{\Gamma N}$ ([110] direction). When the rotation angle of the sample is close to 117°, the minimum incident angle is observed of anticrossings appeared in normalized wave vector $\frac{\mathbf{k}a}{2\pi} approx.{\pm}0.38\frac{2\pi}{a_{\mathrm{BPI}}}$ and the corresponding ncident

angle is the non-high-symmetry-point $L_{\perp}$ (the perpendicular foot of Γ on $\overline{\mathrm{HP}}$). When the sample is rotated close to 63°, one maximum is observed is considered as $\overline{\mathrm{NP}}$ direction. The other maximum of the anticrossing angle also exceeds the observable range when the sample is rotated close to 153°, but we can still determine that the azimuthal angle as the $\overline{\mathrm{NH}}$ direction.

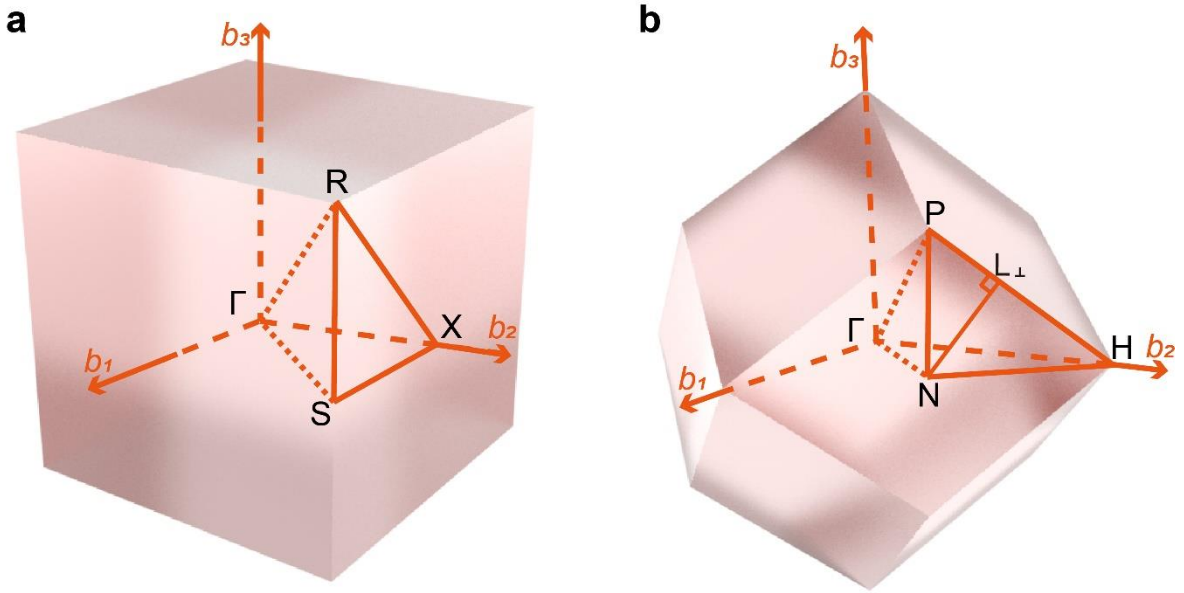

Supplementary Fig. 64 Irreducible BZs with symmetry points of (**a**) BPII and (**b**) BPI.

Irreducible BZ of BPII is a cubic (a), and its high symmetry points are labeled by $\mathbf{\Gamma}$, *S*, *R*, and *X*. The coordinates of the points are Γ: $(0,0,0)\frac{2\pi}{a_{\mathrm{BPII}}}$, *S*: $(\frac{1}{2},\frac{1}{2},0)\frac{2\pi}{a_{\mathrm{BPII}}}$, *R*: $(\frac{1}{2},\frac{1}{2},\frac{1}{2})\frac{2\pi}{a_{\mathrm{BPII}}}$ and *X*: $(0,\frac{1}{2},0)\frac{2\pi}{a_{\mathrm{BPII}}}$ (a_{BPII} is the lattice constant of BPII of 167.33 nm, Supplementary Fig. 48). The distance among high symmetry points of BPII in *k*-space are calculated and further normalized by $k_0=\frac{2\pi}{a_{\mathrm{BPII}}}=0.0375\ \mathrm{nm}^{-1}$. The distance between *X* and *S* points XS=0.0188 nm^{-1} (normalized wave vector of $0.5002\frac{2\pi}{a_{\mathrm{BPII}}}$) and distance between *X* and *R* points XR=0.0265 nm^{-1} (normalized wave vector of $0.7072\frac{2\pi}{a_{\mathrm{BPII}}}$). Compared with Figure 3C$_2$ in the main text showing the observable range of normalized wave vector is around $\pm 0.33\frac{2\pi}{a_{\mathrm{BPII}}}$, the distance between each BPII's BZ edge and the normal incident angle is beyond the observable range. As for BPI, the shape of irreducible BZ of BPI is a regular dodecahedron and its high symmetry points are labeled by $\mathbf{\Gamma}$, *N*, *P*, and *H* where a non-high-symmetry-point $L_{\perp}$ represents the perpendicular foot of *N* on $\overline{HP}$. The coordinates of the points are Γ: $(0,0,0)\frac{4\pi}{a_{\mathrm{BPI}}}$, *N*: $(\frac{1}{4},\frac{1}{4},0)\frac{4\pi}{a_{\mathrm{BPI}}}$, *P*: $(\frac{1}{4},\frac{1}{4},\frac{1}{4})\frac{4\pi}{a_{\mathrm{BPI}}}$ and *H*: $(0,\frac{1}{2},0)\frac{4\pi}{a_{\mathrm{BPI}}}$ (a_{BPI} is the lattice constant of BPI of 254.48 nm, Supplementary Fig. 48). The

distance among high symmetry points of BPII in k-space are calculated and further normalized by k_0=$\frac{2\pi}{a_{\mathrm{BPI}}}$=0.0247 nm^{-1}. The distance between *N* and *P* points NP=0.0123 nm^{-1} (normalized wave vector of 0.4984$\frac{2\pi}{a_{\mathrm{BPI}}}$ agrees well with Figure 3B_2 in the main text). The distance between *N* and $L_\perp$ points is NL$_\perp$ =0.0100 nm^{-1} (normalized wave vector of 0.4065$\frac{2\pi}{a_{\mathrm{BPI}}}$ agrees well with Figure 3B_3, in the main text). The distance between *N* and H points NH=0.0173 nm^{-1} (normalized wave vector of 0.7027$\frac{2\pi}{a_{\mathrm{BPI}}}$, indicating this anticrossings are beyond the observable range (around ±0.63$\frac{2\pi}{a_{\mathrm{BPI}}}$ in Figure 3B_4 in the main text))

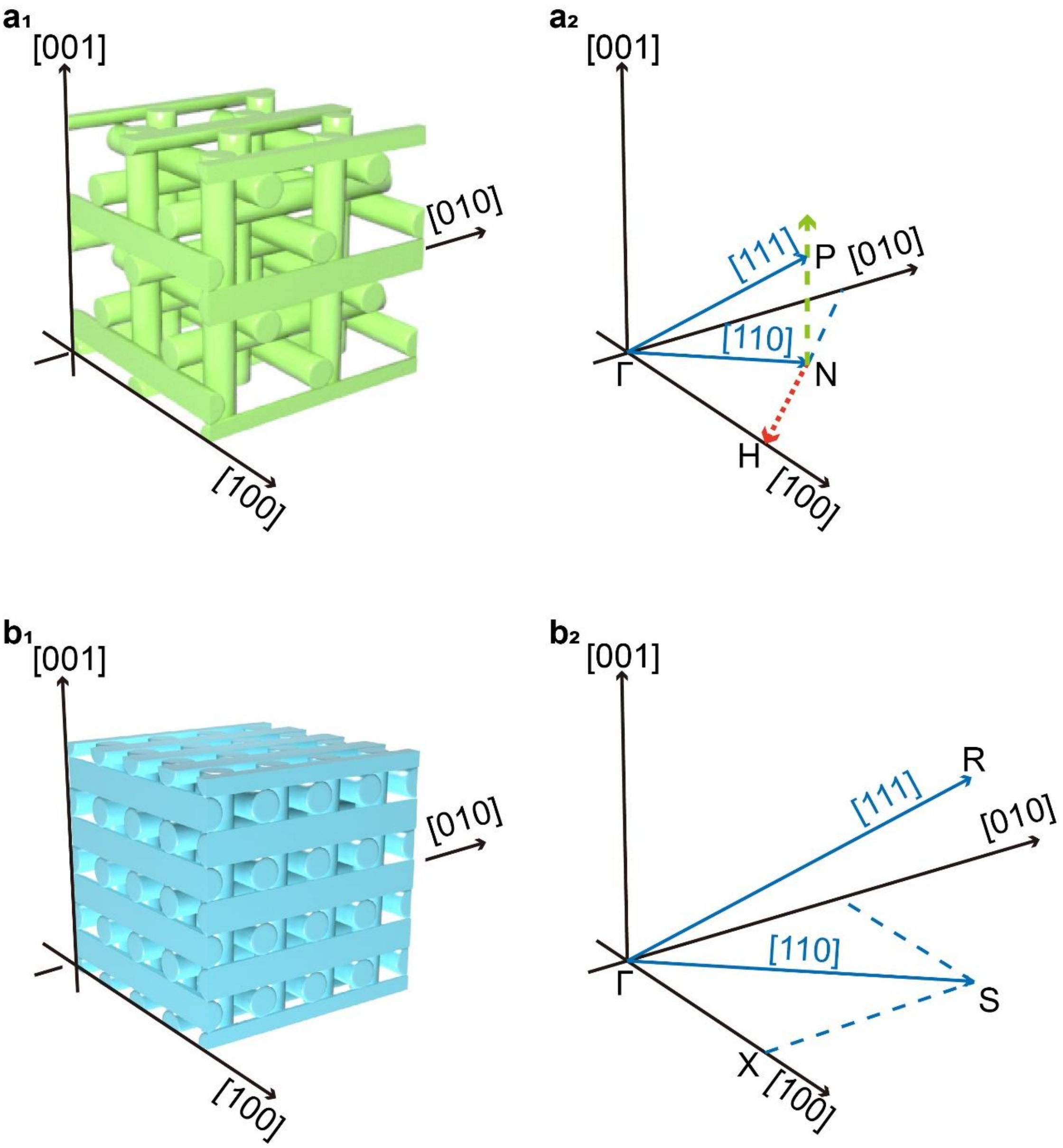

Supplementary Fig. 65 Direction of measured transmittance and endpoints of BZ are highlighted in the real-space model. a_1 Model of 8 unit cells (2×2×2) of BPI. **a_2** Direction of $\overline{NP}$ highlighted by green dash arrow and $\overline{NH}$ highlighted by a red dotted arrow in a coordinate which is the same as (**a_1**). **b_1** Model of 64 unit cells (4×4×4) of BPII. **b_2** Endpoints of BZ are labeled *X*, *M*, and *R* in a coordinate that is the same as (**b_1**). The labels of *P*, *N*, *H*, and **Γ** (*R*, *M*, *X*, and **Γ**) represent the high-symmetry directions for BPI (BPII).

The high symmetry points in reciprocal space of *P*, *N*, *H*, and **Γ** (a_1) are labeled in real-space BPI model (a_2) and *R*, *M*, *X*, and **Γ** (b_1) are labeled in real-space BPII model (b_2). The coordinates used here are the same.

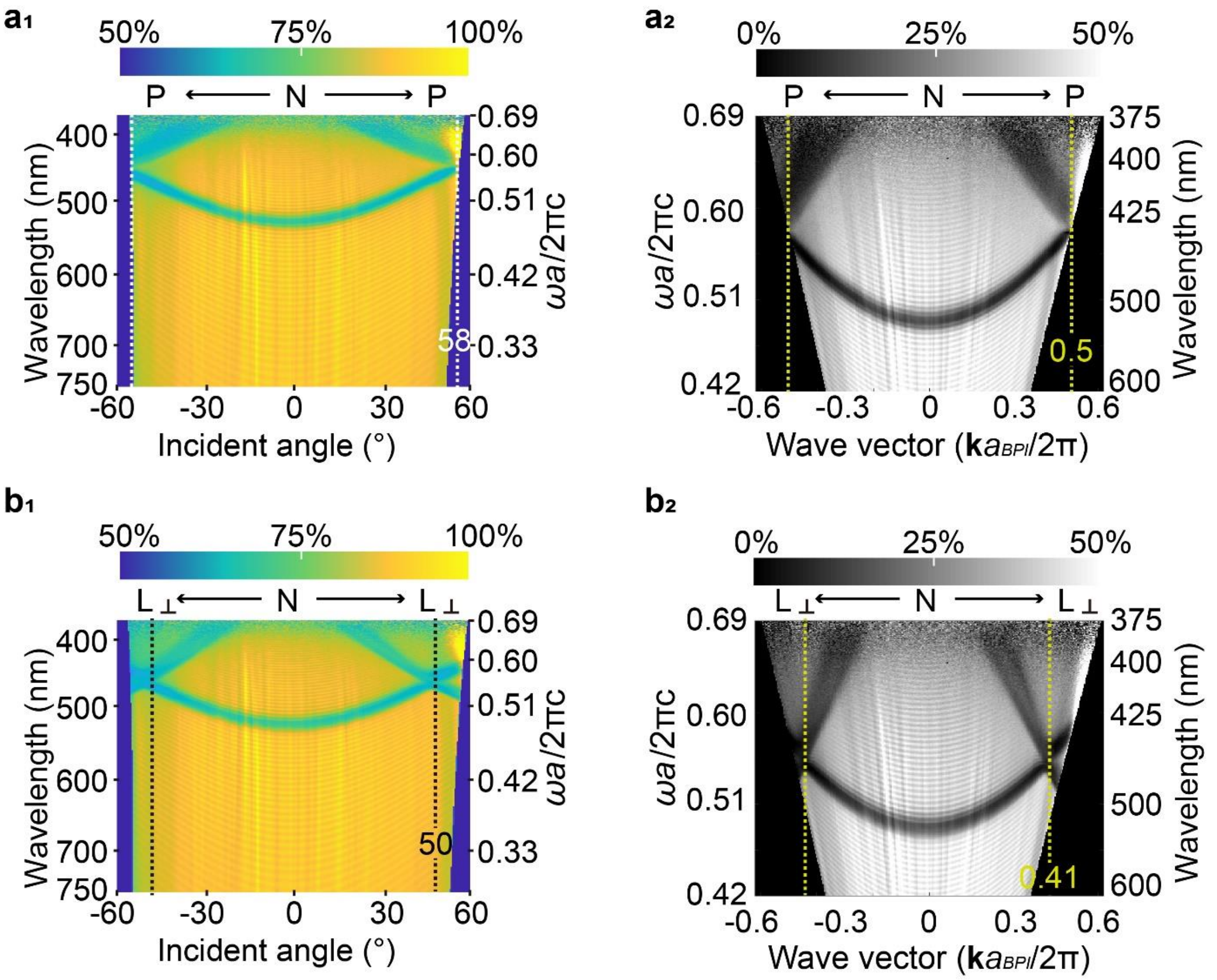

Supplementary Fig. 66 Contour plots of measured transmittance of monodomain polymer-stabilized BPI$_{\{110\}}$ along (**a$_1$, a$_2$**) $N \rightarrow P$, (**b$_1$, b$_2$**) $N \rightarrow L_\perp$ and direction. The color scale indicates the reflectivity.

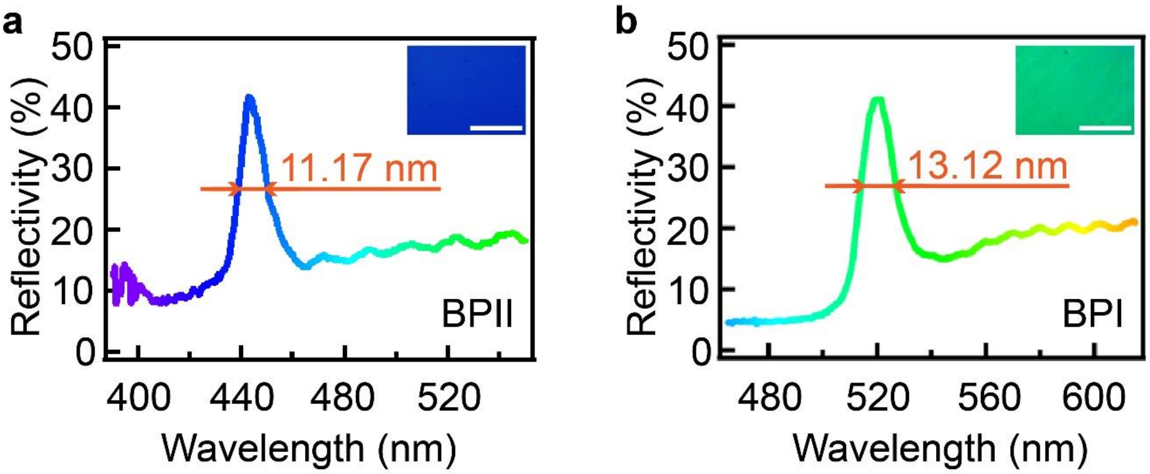

Supplementary Fig. 67 Reflection spectra of (**a**) monodomain $BPII_{\{100\}}$ and (**b**) $BPI_{\{110\}}$. Inserted images is POM images of monodomain $BPII_{\{100\}}$ and $BPI_{\{110\}}$. The scale bar is 400 nm.

The FWHM of reflection spectra of $BPI_{\{110\}}$ and $BPII_{\{100\}}$ reflect the width of the photonic bandgap to a certain extent. In this case, the FWHM of $BPII_{\{100\}}$ of 11.17 nm is narrower than that of $BPI_{\{110\}}$ (13.12 nm), which agrees with the calculated band structures (see Figures $1b_5$, $1c_3$ in the main text).

In addition, the crystal lattices of monodomain BPLCs have higher quality than polydomain BPLCs. The FWHM of monodomain BPI (BPII) is 13.12 (11.17) nm which is slightly narrower than the polydomain BPI (BPII) of 13.61 (13.67) nm (see Figure. $1E_4$ in the main text).

Supplementary Note 7 Lasing properties of the DLPT of BPLCs

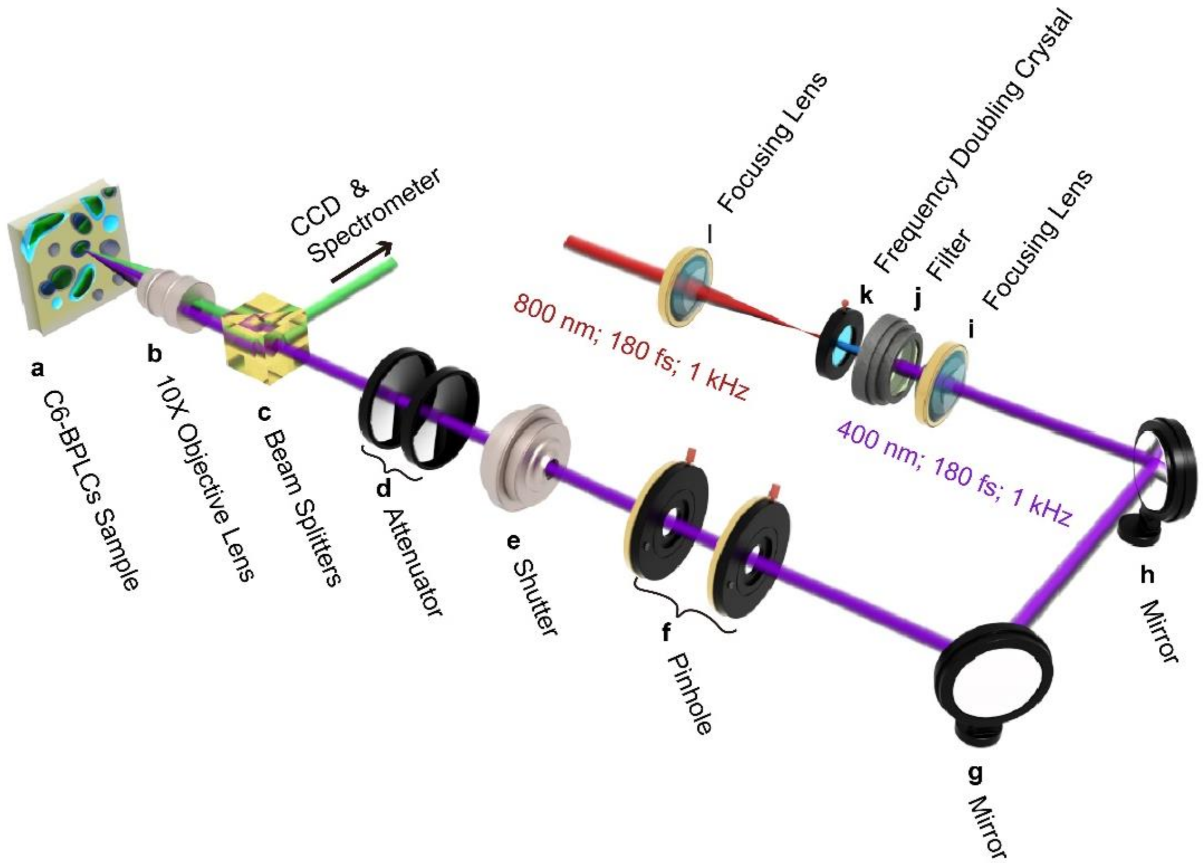

Supplementary Fig. 68 Configuration of laser experiment for measuring the fluorescence and lasing spectra of the C6-BPLCs. a: C6 dopped self-standing PS-BPLCs sample in each stage; **b**: 10X objective lens (NA=0.3); **c**: beam splitters; **d**: attenuator; **e**: shutter; **f**: pinhole; **g**: mirror; **h**: mirror; **i**: focusing lens; **j**: filter **k**: frequency doubling crystal; **l**: focusing lens; CCD is the abbreviation of ‘Charge-coupled Device’

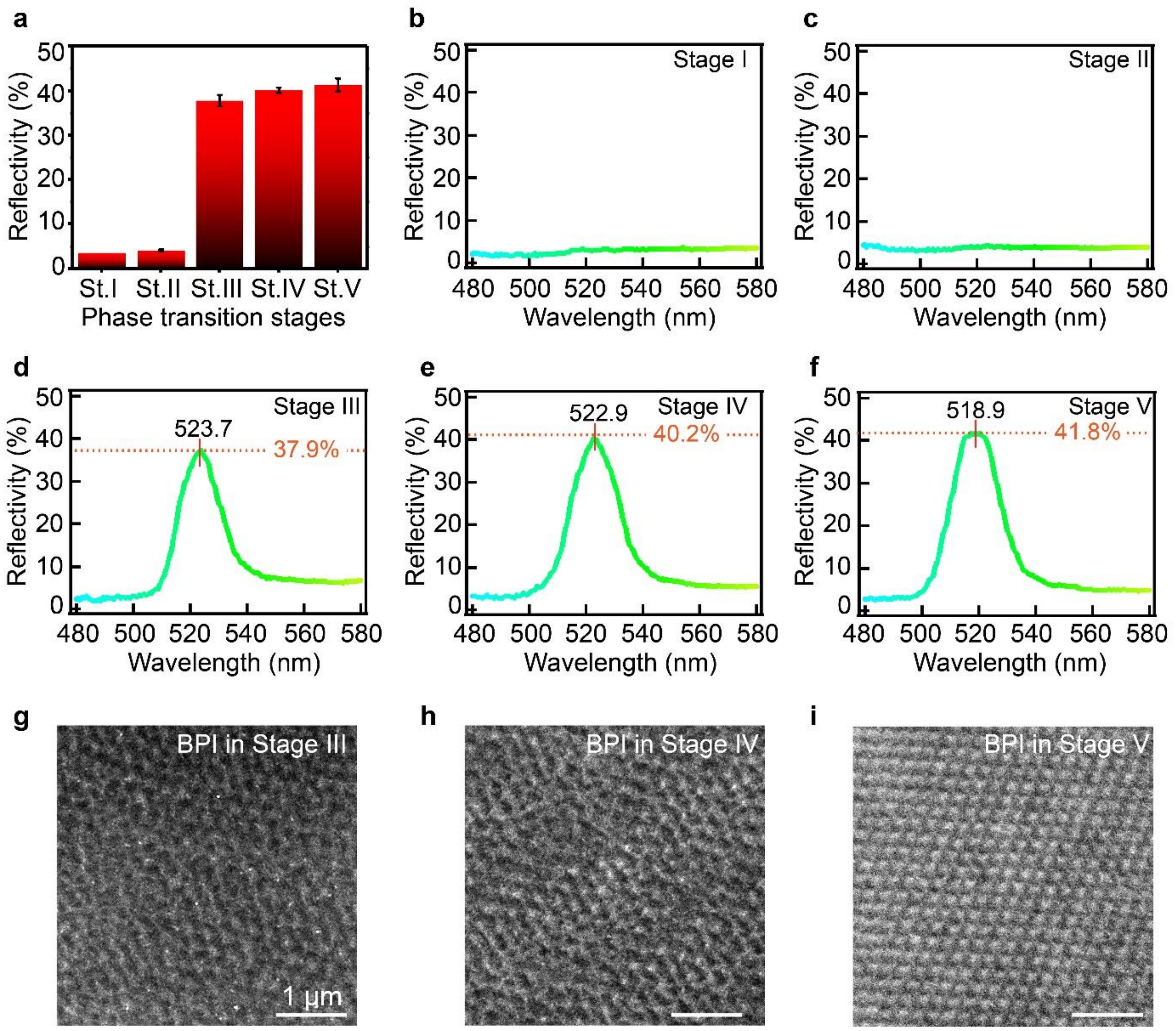

Supplementary Fig. 69 *In situ* reflection spectra of C6-BPLCs utilized for measurement of lasing properties of Figure 5 in the main text. a Overview of reflectivity in distinct stages ‘St. I’ to ‘St. V’ refer to ‘Stage I’ to ‘Stage V’. Reflection spectra of (**b**) Stage I, (**c**) Stage II, (**d**) Stage III, (**e**) Stage IV, and (**f**) Stage V. TEM images of BPI in (**g**) Stage III, (**h**) Stage IV, and (**i**) Stage V.

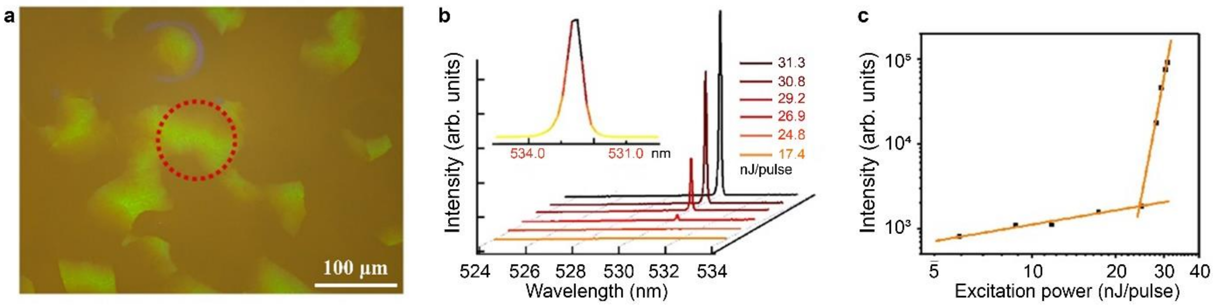

Supplementary Fig. 70 Lasing properties of C6-BPLCs film at Stage IV polymerized at 76.2 °C (Stage IV$_{76.2}$). a Textures captured by an inverted optical microscope in reflection mode. The red dotted circle highlights the excited blue phase platelet. **b** The emission spectra plotted relative to the low-energy photonic bandedge, where the inset is the lasing peak on a magnified wavelength scale. **c** Typical input-output curve. The solid lines represent the best fit to the data below and above the excitation threshold.

Lasing properties in Stage IV of C6-BPLCs polymerized at 76.2 °C (Stage IV$_{76.2}$) are studied. Single-mode lasing is realized at 530.70 nm with an FWHM of 0.107 nm and Q factor of 4960. It exhibits a clear threshold of 24.50 nJ/pulse as a function of pump energy.

Supplementary References

1. Gandhi, S. S. & Chien, L. C., Unraveling the Mystery of the Blue Fog: Structure, Properties, and Applications of Amorphous Blue Phase III. *Adv. Mater.* **2017,** *29* (47), 1704296-1704309 (2017).
2. Chiang, I. H., Long, C. J., Lin, H. C., Chuang, W. T., Lee, J. J. & Lin, H. C., Broad ranges and fast responses of single-component blue-phase liquid crystals containing banana-shaped 1,3,4-oxadiazole cores. *ACS Appl. Mater. Interfaces* **2014,** *6* (1), 228-235 (2014).
3. Kim, M. S. & Chien, L. C., Topology-mediated electro-optical behaviour of a wide-temperature liquid crystalline amorphous blue phase. *Soft Matter.* **2015,** *11* (40), 8013-8018 (2015).
4. Henrich, O., Stratford, K., Marenduzzo, D & Cates, M. E., Ordering dynamics of blue phases entails kinetic stabilization of amorphous networks. *Proc. Natl. Acad. Sci. U. S. A.* **2010,** *107* (30), 13212-13215 (2010).
5. Yang, J. J., Liu, J., Guan, B., He, W. L., Yang, Z., Wang, J. X., Ikeda, T & Jiang, L., Fabrication and photonic applications of large-domain blue phase films. *J. Mater. Chem. C* **2019,** *7* (31), 9460-9466 (2019).
6. Jo, S. Y., Jeon, S. W., Kim, B. C., Bae, J. H., Araoka, F. & Choi, S. W., Polymer Stabilization of Liquid-Crystal Blue Phase II toward Photonic Crystals. *ACS Appl. Mater. Interfaces* **2017,** *9* (10), 8941-8947 (2017).
7. Castles, F., Day, F. V., Morris, S. M., Ko, D. H., Gardiner, D. J., Qasim, M. M., Nosheen, S., Hands, P. J., Choi, S. S., Friend, R. H. & Coles, H. J., Blue-phase templated fabrication of three-dimensional nanostructures for photonic applications. *Nat. Mater.* **2012,** *11* (7), 599-603 (2012).
8. Tanaka, S., Yoshida, H., Kawata, Y., Kuwahara, R., Nishi, R. & Ozaki, M., Double-twist cylinders in liquid crystalline cholesteric blue phases observed by transmission electron microscopy. *Sci. Rep.* **2015, 5**, 16180-16189 (2015).
9. Sala-Tefelska, M. M., Orzechowski, K., Sierakowski, M., Siarkowska, A., Woliński, T. R., Strzeżysz, O & Kula, P., Influence of cylindrical geometry and alignment layers on the growth process and selective reflection of blue phase domains. *Opt. Mater.* **2018,** *75*, 211-215 (2018).
