# Supplementary material for: Diffusionless transformation of soft cubic superstructure from amorphous to simple cubic and body-centered cubic phases
Source: Nat Commun. 2021 Jun 9;12:3477. doi: 10.1038/s41467-021-23631-w (PMC8190294; doi:10.1038/s41467-021-23631-w)
Supplement: Supplementary file 3 — Description of Additional Supplementary Files [file 41467_2021_23631_MOESM3_ESM.pdf]

Description of Additional Supplementary Files

Title: Supplementary Movie 1

Description: In situ POM observation is performed on phase transformation of polydomain BPLCs before polymer-stabilization when the sample was cooled from 79.4 ℃ at 0.05 ℃/min.

Title: Supplementary Movie 2

Description: First 15 circles were recorded by in situ POM to investigate the speed and reversibility of thermoelastic DLPT between BPI and BPII upon cold air flowing.

Title: Supplementary Movie 3

Description: In situ POM characterization on phase transformation of monodomain BPLCs before polymer-stabilization when the sample was cooled from 79.4 ℃ at 0.05 ℃/min.
